# Comparison of Acupuncture vs Sham Acupuncture or Waiting List Control in the Treatment of Aromatase Inhibitor–Related Joint Pain: A Randomized Clinical Trial

**DOI:** 10.1001/jamanetworkopen.2022.41720

**Published:** 2022-11-11

**Authors:** Dawn L. Hershman, Joseph M. Unger, Heather Greenlee, Jillian Capodice, Danika L. Lew, Amy Darke, Lori M. Minasian, Michael J. Fisch, N. Lynn Henry, Katherine D. Crew

**Affiliations:** 1 Columbia University Irving Medical Center, New York, New York; 2 Fred Hutchinson Cancer Center, Seattle, Washington; 3 SWOG Statistics and Data Management Center, Seattle, Washington; 4 Department of Urology, Mount Sinai Hospital, New York, New York; 5 Division of Cancer Prevention, National Cancer Institute, Bethesda, Maryland; 6 AIM Specialty Health, Chicago, Illinois; 7 Department of Medicine, University of Michigan, Ann Arbor

## Abstract

**Importance:**

Aromatase inhibitors (AIs) have proven efficacy for the treatment of hormone-sensitive breast cancer; however, arthralgias (pain and stiffness) contribute to nonadherence with therapy for more than 50% of patients.

**Objective:**

To examine the effect of acupuncture in reducing AI-related joint pain through 52 weeks.

**Design, Setting, and Participants:**

A randomized clinical trial was conducted at 11 sites in the US from May 1, 2012, to February 29, 2016, with a scheduled final date of follow-up of September 5, 2017, to compare true acupuncture (TA) with sham acupuncture (SA) or waiting list control (WC). Women with early-stage breast cancer were eligible if they were taking an AI and scored 3 or higher on the Brief Pain Inventory Worst Pain (BPI-WP) item (score range, 0-10; higher scores indicate greater pain). Analysis was conducted for data received through May 3, 2021.

**Interventions:**

Participants were randomized 2:1:1 to the TA (n = 110), SA (n = 59), or WC (n = 57) group. The TA and SA protocols were composed of 6 weeks of intervention at 2 sessions per week (12 sessions overall), followed by 6 additional weeks of intervention with 1 session per week. Participants randomized to WC received no intervention. All participants were offered 10 acupuncture sessions to be used between weeks 24 and 52.

**Main Outcomes and Measures:**

In this long-term evaluation, the primary end point was the 52-week BPI-WP score, compared by study group using linear regression, adjusted for baseline pain and stratification factors.

**Results:**

Among 226 randomized women (mean [SD] age, 60.7 [8.6] years; 87.7% White; mean [SD] baseline BPI-WP score, 6.7 [1.5]), 191 (84.5%) completed the trial. In a linear regression, 52-week mean BPI-WP scores were 1.08 (95% CI, 0.24-1.91) points lower in the TA compared with the SA group (*P* = .01) and were 0.99 (95% CI, 0.12-1.86) points lower in the TA compared with the WC group (*P* = .03). In addition, 52-week BPI pain interference scores were statistically significantly lower in the TA compared with the SA group (difference, 0.58; 95% CI, 0.00-1.16; *P* = .05). Between 24 and 52 weeks, 12 (13.2%) of TA, 6 (11.3%) of SA, and 5 (10.6%) of WC patients reported receipt of acupuncture.

**Conclusions and Relevance:**

In this randomized clinical trial, women with AI-related joint pain receiving 12 weeks of TA had reduced pain at 52 weeks compared with controls, suggesting long-term benefits of this therapy.

**Trial Registration:**

ClinicalTrials.gov Identifier: NCT01535066

## Key Points

**Question:**

Does short-term acupuncture confer long-term reduction of joint pain related to aromatase inhibitors among women with breast cancer?

**Findings:**

In this multicenter randomized clinical trial of 226 women with early-stage breast cancer, patients in the true acupuncture group who received 12 weeks of acupuncture, compared with those in the sham acupuncture group or the waiting list control group, had statistically significant reductions in joint pain scores at 52 weeks.

**Meaning:**

Acupuncture was associated with a statistically significant decrease in aromatase inhibitor–related joint pain that persisted at 40 weeks after discontinuation of the intervention, suggesting long-term benefits of this therapy.

## Teaser

This randomized clinical trial of women with breast cancer assesses whether short-term acupuncture reduces long-term aromatase inhibitor–related joint pain compared with sham acupuncture or a waiting list control.

## Introduction

Aromatase inhibitors (AIs) have proven efficacy for the treatment of hormone-sensitive breast cancer.^1^ For more than 50% of patients, however, arthralgias (pain and stiffness) contribute to nonadherence with therapy.^2^ A previous report^3^ of results from Southwest Oncology Group (SWOG) S1200, a multicenter, blinded sham acupuncture (SA) and waiting list control (WC) randomized clinical trial that was conducted to evaluate the effect of true acupuncture (TA) on joint pain related to AIs among women with early-stage breast cancer, found that TA compared with SA or WC resulted in a statistically significant reduction in joint pain at 6 weeks, the primary end point, and at 12 weeks. Subsequent systemic reviews and meta-analyses^4,5^ have confirmed this effect.

The duration of persistent benefit of acupuncture after a course of treatment is uncertain. A meta-analysis^5^ of acupuncture trials with longer-term follow-up suggested that treatment effects persist for up to 12 months. Specifically, the authors reported only a 10% reduction in the long-term beneficial effect of TA when compared with usual care and a 50% reduction in effect when compared with SA.^5^ The trials were heterogeneous in terms of design, condition, and duration of treatment. To address the sustained benefit of acupuncture among women with AI-induced arthralgias, we now report the 52-week results from SWOG S1200.

## Methods

### Eligibility and Study Conduct

Details of the study have been previously reported.^3,6^ In brief, eligible study participants were postmenopausal women with stage 1 to 3 breast cancer taking a third-generation AI for 30 days or more before registration. Race and ethnicity, as reported by the study team, are provided to interpret the generalizability of the results. Inclusion criteria included a score of 3 or higher (range of 0-10, with higher scores indicating greater pain) on the Brief Pain Inventory Worst Pain (BPI-WP) item. From May 1, 2012, to February 29, 2016, a total of 226 patients were randomly assigned to TA (n = 110), SA (n = 59), or WC (n = 57), with a final date of follow-up of September 5, 2017 (eFigure in Supplement 2). The study was conducted at 11 academic and community sites within the National Cancer Institute Community Oncology Research Program. Sites were required to have 2 trained acupuncturists for the duration of the trial. The trial protocol can be found in Supplement 1. The study was approved by the local institutional review boards for the study sites, and participants were informed of the investigational nature of the study and provided written informed consent. This study followed the Consolidated Standards of Reporting Trials (CONSORT) reporting guideline.

### Study Intervention

Study participants were randomized 2:1:1 to TA vs SA vs WC, with randomization dynamically balanced by study site. Both TA and SA consisted of twelve 30- to 45-minute sessions administered during 6 weeks (2 sessions per week) followed by 1 session per week for 6 more weeks. For TA, stainless steel, single-use, sterile, and disposable needles were used and inserted at traditional depths and angles. The SA protocol consisted of a core standardized prescription of minimally invasive, shallow needle insertion using thin and short needles at nonacupuncture points. The SA protocol also included joint-specific treatments and an auricular sham based on the application of adhesives to nonacupuncture points on the ear. The WC group received no acupuncture during the initial 24 weeks of study participation. At 24 weeks, all patients received vouchers for 10 TA sessions to be used before the 52-week visit.

### Outcomes

The original protocol-specified primary end point was the BPI-WP score at 6 weeks.^7^ The short-form version of the BPI was administered at 6, 12, 16, 20, 24, and 52 weeks. For this long-term analysis, the primary end point was the 52-week assessment of BPI-WP (which was not previously reported), examined using multivariable linear regression. Secondary end points for this long-term evaluation included the BPI average pain, pain interference, pain severity, and worst stiffness scores at 52 weeks. All BPI scores range from no symptoms to worst on a 0- to 10-point scale (with higher scores indicating worse symptoms). In addition, we evaluated pain using the PROMIS Pain Interference–Short Form (PROMIS PI-SF), which has scores ranging from 6 to 30.^8^ This instrument was also administered at 6, 12, 24, and 52 weeks and has 5 response levels (low scores [not at all] to high scores [very much]), with higher scores reflecting worse symptoms.

We examined 2 functional measures: grip strength and Timed Get Up and Go test. Grip strength was measured with a digital hand grip strength dynamometer (DHS 88, Detecto) in kilograms.^9^ Patients were asked to make 3 maximal voluntary contractions with 1 minute between each. The maximum contraction was used. The Timed Get Up and Go test is a physical function assessment tool of speed that is an estimate of impairments in balance and gait.^10^

### Statistical Analysis

The sample size determination for the protocol-specified primary end point at 12 weeks was previously described.^3^ In brief, 208 eligible patients provided 82% power to compare TA with SA and, separately, with WC with 2-sided α = .025 tests to account for 2 independent comparisons. For this long-term evaluation, the primary hypothesis was that TA would decrease joint pain associated with the use of AIs compared with SA or WC at 52 weeks. No adjustments for multiple comparisons were made for all secondary or post hoc analyses, which are considered exploratory. Under intention-to-treat, all evaluable 52-week assessments were used, even if control group patients received TA after 24 weeks. The 52-week BPI-WP scores were compared by group using multivariable linear regression adjusting for the baseline score and indicator variables for study sites, with 2 indicator variables to represent the different intervention groups. In addition, we examined BPI-WP scores at 6, 12, 16, 20, and 24 weeks using multivariable linear regression. Other BPI domains and the PROMIS PI-SF scores were evaluated at individual assessment times using linear regression. Longitudinal analyses using all assessments for each patient-reported outcome domain through week 52 were conducted using linear mixed models, with individuals considered random effects; assessment time (as both a linear and quadratic function) and its potential interaction with treatment were considered fixed effects. Regression analyses included covariate adjustment for the baseline score, indicator variables for study sites, and 2 indicator variables for intervention group. The proportion of patients by group who discontinued use of AIs or who used any pain medications (including acetaminophen, ibuprofen, other nonsteroidal anti-inflammatory drugs, or narcotics) after the initial on-study assessment was tested using a χ^2^ test. Two-sided α = .05 tests were deemed statistically significant. Statistical analyses were conducted using SAS software, version 9.4 (SAS Institute Inc).^11^

## Results

A total of 226 women were randomized (mean [SD] age, 60.7 [8.6] years; 15 [6.8%] Asian, 10 [4.5%] Black, 1 [0.5%] American Indian, 1 [0.5%] Pacific Islander, and 193 [87.7%] White; 21 [9.3%] Hispanic and 204 [90.7%] non-Hispanic; mean [SD] baseline BPI-WP score, 6.7 [1.5]), of whom 205 (90.7%) completed the trial. A total of 111 patients (49.1%) had received prior chemotherapy, and the median time receiving AI therapy was 1.1 years (range, 0.1-9.0 years). Patient characteristics were well balanced by group (Table).

**Table.** Patient Characteristics^a^

Characteristic	Overall (N = 226)	Intervention arm		
		True acupuncture (n = 110)	Sham acupuncture (n = 59)	Waiting list control (n = 57)
Age, median (range), y	60.7 (27.0-80.6)	60.7 (34.1-80.6)	57.0 (40.5-77.5)	60.6 (27.0-76.0)
Hispanic				
Yes	21 (9.7)	11 (10.0)	7 (12.1)	3 (5.3)
No	204 (90.3)	99 (90.0)	51 (87.9)	54 (94.7)
Unknown	1	0	1	0
Race				
Asian	15 (6.8)	11 (10.4)	2 (3.4)	2 (3.6)
Black	10 (4.5)	6 (5.7)	2 (3.4)	2 (3.6)
American Indian	1 (0.5)	1 (0.9)	0	0
Pacific Islander	1 (0.5)	0	0	1 (1.8)
White	193 (87.7)	88 (83.0)	54 (93.1)	51 (91.1)
Unknown	6	4	1	1
Breast cancer stage				
I	97 (43.7)	41 (38.7)	28 (47.5)	28 (49.1)
II	99 (44.6)	53 (50.0)	23 (39.0)	23 (40.4)
III	26 (11.7)	12 (11.3)	8 (13.6)	6 (10.5)
Unknown	4	4	0	0
Prior chemotherapy	111 (49.1)	56 (50.9)	31 (52.5)	24 (42.1)
Prior tamoxifen	43 (19.0)	18 (16.4)	15 (25.4)	10 (17.5)
Current or prior AI therapy^b^				
Anastrozole	164 (72.6)	80 (72.7)	44 (74.6)	40 (70.2)
Letrozole	70 (31.0)	36 (32.7)	17 (28.8)	17 (29.8)
Exemestane	41 (18.1)	21 (19.1)	10 (16.9)	10 (17.5)
Time receiving AI therapy, median (range), y	1.1 (0.1-9.0)	1.0 (0.1-8.0)	1.1 (0.1-9.0)	1.1 (0.1-3.1)
Prior acupuncture	44 (19.5)	19 (17.3)	13 (22.0)	12 (21.1)

Abbreviation: AI, aromatase inhibitor.

^a^ Data are presented as number (percentage) of patients unless otherwise indicated. Percentages are calculated among those with known data.

^b^ Number answering yes (>1 type allowed).

### BPI-WP Scores 

**Figure 1.**
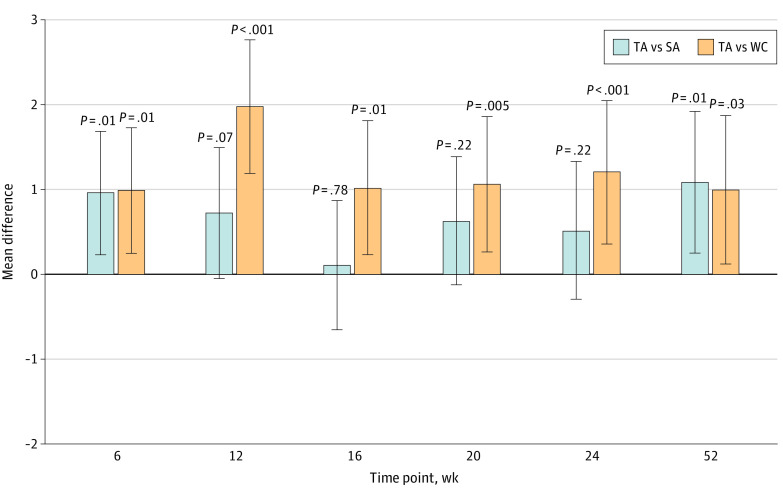
Adjusted Mean Group Difference in Brief Pain Inventory Worst Pain Scores in True Acupuncture (TA) vs Sham Acupuncture (SA) and TA vs Waiting List Control (WC) at 6, 12, 16, 20, 24, and 52 Weeks The vertical lines indicate the 95% CIs. For these individual time point–specific analyses, linear regression was used.

In total, 91 of the 110 patients (82.7%) assigned to TA, 53 of the 59 (89.8%) assigned to SA, and 47 of the 57 (82.5%) assigned to WC had both baseline and 52-week BPI-WP scores available for analysis. For patients with 52-week scores, the mean (SD) baseline BPI-WP was 6.77 (1.51) in the TA group, 6.38 (1.55) in the SA group, and 6.40 (1.54) in the WC group. Compared with baseline, the observed BPI-WP was 2.72 points lower (reduced pain) at 52 weeks in the TA group, 1.46 points lower in the SA group, and 1.55 points lower in the WC group, with differences in adjusted 52-week mean BPI-WP scores of 1.08 points (95% CI, 0.24-1.91 points) between the TA and SA groups (*P* = .01) and 0.99 points (95% CI, 0.12-1.86 points) between the TA and WC groups (*P* = .03) (Figure 1; eTable 1 in Supplement 2). Of note, although the difference in mean scores between the TA and SA groups was maximized at 52 weeks, the difference at 52 weeks between the TA and WC groups was approximately half of the maximum difference observed at 12 weeks (1.97 points; 95% CI, 1.19-2.75; *P* < .001) (eTable 1 in Supplement 2). Adjusted mean differences for TA compared with SA were less than 1.0 and not statistically significant for weeks 12, 16, 20, and 24; in contrast, adjusted mean differences for TA compared with WC were all greater than 1.0 and statistically significant at these interim assessment times (Figure 1; eTable 1 in Supplement 2). There was no statistically significant difference in BPI-WP scores between WC and SA at 52 weeks (adjusted mean difference, –0.09; 95% CI, –1.05 to 0.87; *P* = .85).

**Figure 2.**
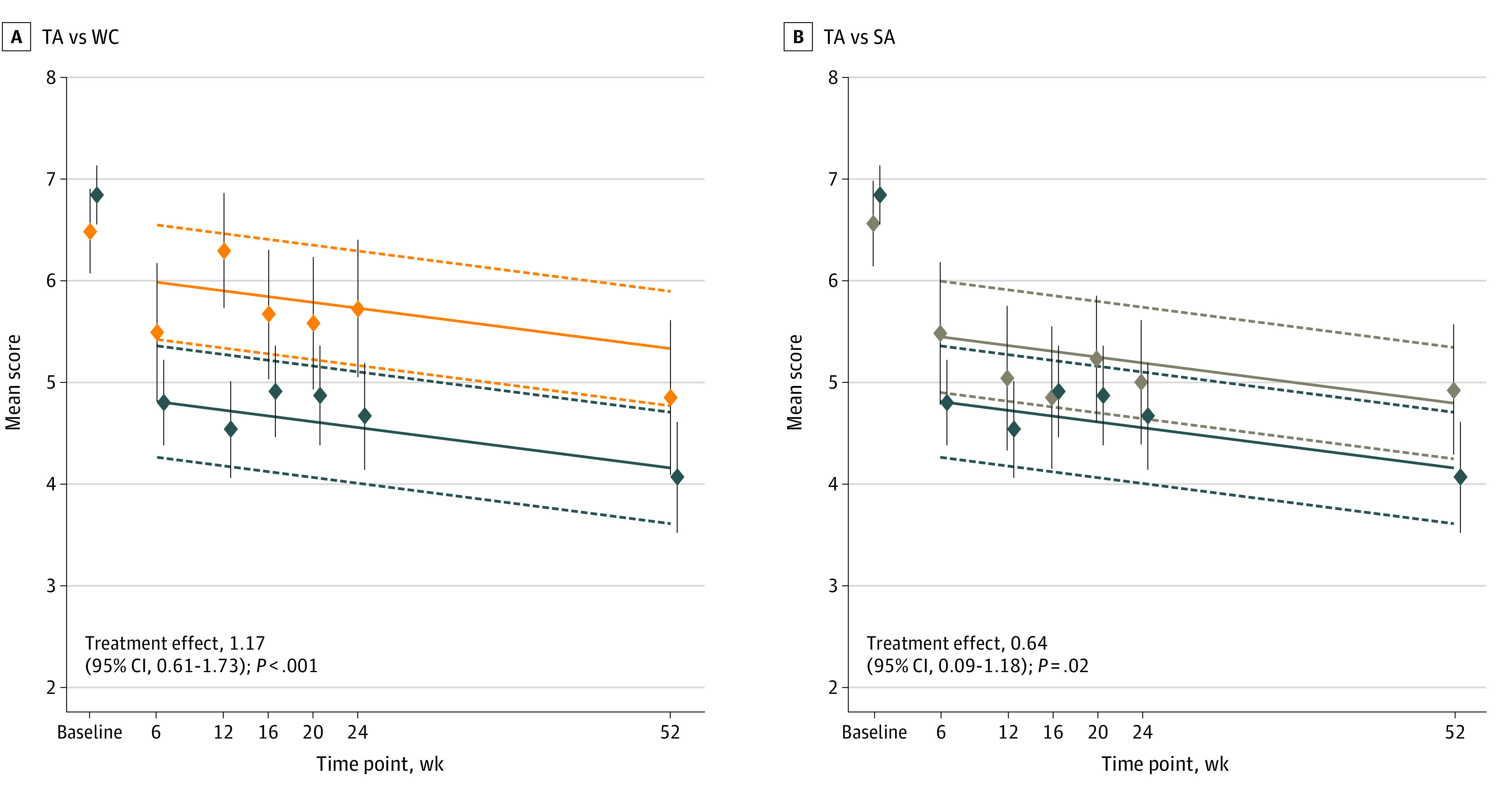
Mean Difference in Brief Pain Inventory Worst Pain Scores Over Time in True Acupuncture (TA) vs Waiting List Control (WC) and Separately in TA vs Sham Acupuncture (SA) Lower scores indicate less pain. For this analysis, linear mixed models were used. Dark blue lines indicate TA; orange lines, WC; and tan lines, SA. The dashed lines represent the lower and upper 95% CIs for the fitted lines. The diamonds indicate the mean observed BPI-WP scores at each assessment time; the vertical lines through each diamond indicate the 95% CIs for the means.

In longitudinal analysis, there was no evidence of an interaction of assessment time and intervention assignment, and the best model included assessment time as a linear variable (eTable 2 in Supplement 2). After adjusting for the baseline scores, the longitudinal analysis showed that the intervention resulted in an initial separation between groups at 6 weeks, which was maintained as a statistically significant, consistent effect by group throughout follow-up with no interaction between intervention assignment and time. Thus, across all assessment times through 52 weeks, mean BPI-WP scores were 1.17 points lower (reduced pain) for TA compared with WC (95% CI, 0.61-1.73 points; *P* < .001) and 0.64 points lower for TA compared with SA (95% CI, 0.09-1.18 points; *P* = .02) (Figure 2). There was no statistically significant difference in mean BPI-WP scores between WC and SA in longitudinal analysis (adjusted mean difference, 0.54 points; 95% CI, –0.10 to 1.17 points; *P* = .10).

Between 24 and 52 weeks, 12 (13.2%) of TA, 6 (11.3%) of SA, and 5 (10.6%) of WC patients reported receipt of acupuncture. Among all enrolled patients, there was no difference by group in completion of intervention (TA, 99/110 [90.0%]; SA, 53/59 [89.8%]; and WC, 50/57 [87.7%]; *P* = .89). The main reason for noncompletion was patient refusal (TA, 9 [8.2%]; SA, 6 [10.2%]; and WC, 6 [10.5%]; *P* = .05). Fifty-two–week BPI pain interference scores were statistically significantly lower in the TA compared with the SA group (difference, 0.58; 95% CI, 0.00-1.16; *P* = .05). In addition, compared with SA, adjusted PROMIS PI-SF scores for those in the TA arm were statistically significantly lower (less pain interference) at 52 weeks (difference, 2.35 points; 95% CI, 0.07-4.63 points; *P* = .04). No other statistically significant differences between arms at the 52-week assessment for any other pain or quality-of-life domains were observed (eTable 1 in Supplement 2).

**Figure 3.**
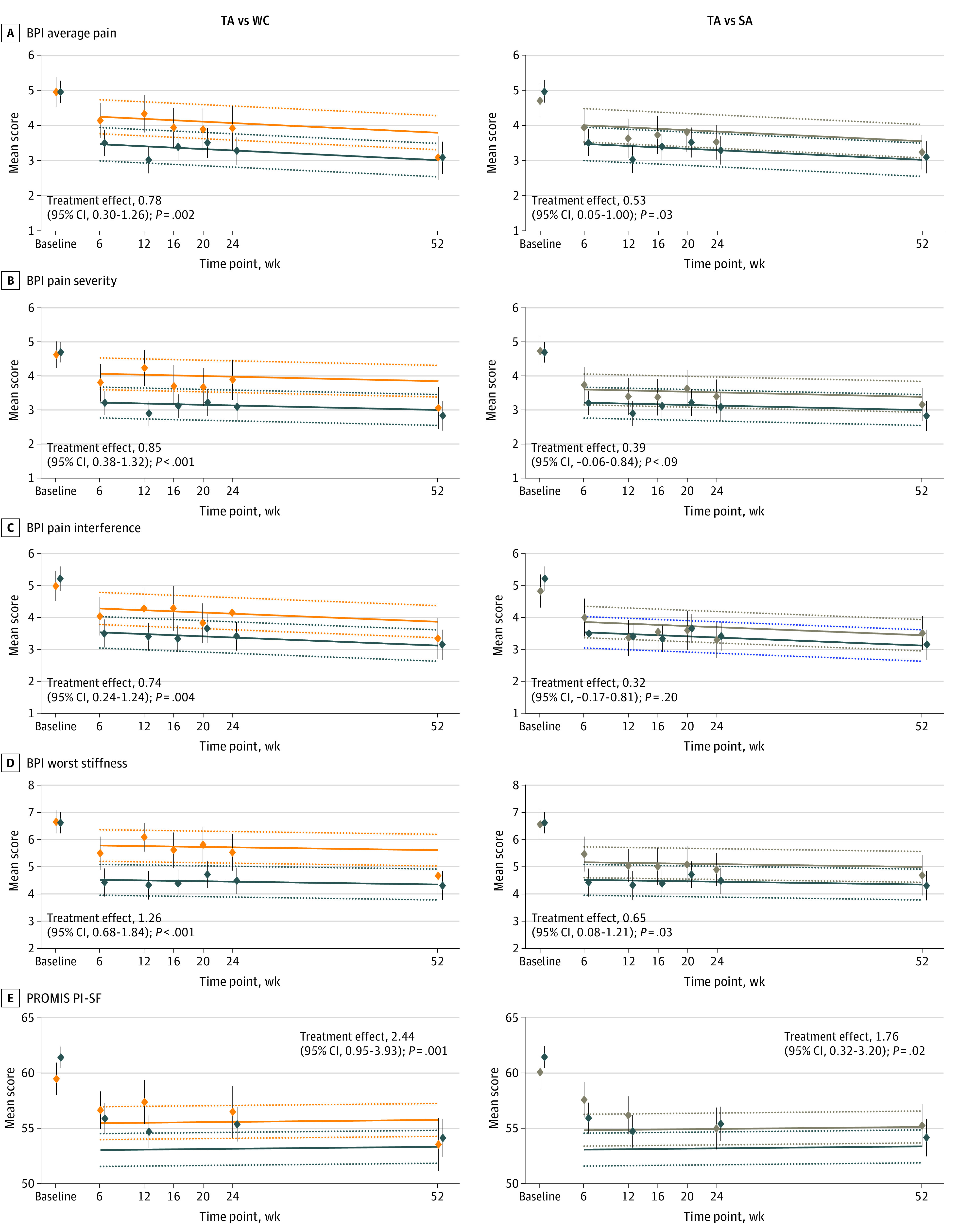
Mean Difference in Brief Pain Inventory (BPI) Scores Over Time for Average Pain, Pain Severity, Pain Interference, Worst Stiffness, and PROMIS Pain Impact–Short Form (PROMIS PI-SF) in True Acupuncture (TA) vs Waiting List Control (WC) and Separately in TA and Sham Acupuncture (SA) Lower scores indicate improvements in the different domains. Linear mixed models were used. Dark blue lines indicate TA; orange lines, WC; and tan lines, SA. The dotted lines represent the lower and upper 95% CIs for the fitted lines. The diamonds indicate the mean observed scores at each assessment time; the vertical lines through each diamond show the 95% CIs for the means.

In longitudinal analysis, there was no evidence of an interaction between assessment time and intervention assignment for any of the secondary pain or quality-of-life domains, and in each case, the best model included assessment time as a linear variable (eTable 2 in Supplement 2). Throughout the entire 52 weeks, the use of TA compared with WC resulted in statistically significant improvements in BPI average pain, pain severity, pain interference, worst stiffness, and PROMIS PI-SF scores, and the use of TA compared with SA results in statistically significant improvements in BPI average pain, worst stiffness, and PROMIS PI-SF scores (Figure 3).

The overall AI discontinuation rate within the 52 weeks of follow-up was 12.1% and did not differ by intervention group. Pain medication use at baseline was common for patients in each of the assigned intervention groups (TA, 66 [60.0%]; SA, 36 [61.0%]; and WC, 33 [57.9%]). To examine incident use of pain medications during the study, we also evaluated patterns among patients who did not report pain medication use at baseline (n = 91 [40.3%]). Among these patients, new use of pain medications during the study occurred in 20 of 44 patients (45.5%) in the TA group, 16 of 23 (69.6%) in the SA group (*P* = .06), and 16 of 24 (66.7%) in the WC group (*P* = .09). Taken together, pain medication use was less likely for patients in the TA compared with the SA or WC groups combined (20 of 44 [45.5%] vs 32 of 47 [68.1%], *P* = .03). No differences were observed between groups in assessments of grip strength (eTables 3 and 4 in Supplement 2) or Timed Get Up and Go (eTables 5 and 6 in Supplement 2) for any assessment time compared with baseline.

## Discussion

In this multicenter, sham- and waiting list–controlled randomized clinical trial of 12 weeks of TA (6 weeks twice weekly followed by 6 weeks weekly) for patients with early-stage breast cancer and AI-related joint pain, statistically significant improvements in pain scores were seen at 52 weeks compared with SA and WC. The maximal adjusted between-group difference between TA and WC at 12 weeks (at the end of the study intervention) was 1.97 points; at 52 weeks, the mean (SD) difference remained at 0.99 points and was also statistically significant. The maximal adjusted mean between-group difference between the TA and SA groups occurred at 52 weeks and was 1.08 points. This difference was relatively constant during the follow-up period. Although there is some uncertainty about the clinical meaning of this between-group difference,^12,13^ the observed difference in this study is consistent with other positive randomized studies^14,15^ of pain control using other interventions, which have reported between-group mean differences ranging from 0.7 to 1.0 points. Furthermore, our results are consistent with a recently reported meta-analysis of 20 827 patients from 39 trials, in which acupuncture was superior to sham as well as no acupuncture control for each pain condition,^16^ as well as a meta-analysis that found that the treatment effects persist for up to 12 months.^5^ Finally, approximately 10% of patients in each of the groups received acupuncture between 24 and 52 weeks, which may have diminished the observed differences between the groups.

Both pharmacologic (duloxetine,^17^ testosterone,^18^ ω3 fatty acids,^19^ and vitamin D^20^) and nonpharmacologic interventions (exercise^21^) have been studied to treat AI-associated arthralgias with mixed results. Most of these trials did not assess or did not find long-term benefits. In addition, the 4 prior studies of acupuncture for AI arthralgias did not evaluate the maintenance or durability of effect.^22,23,24,25^ Our results support findings from prior studies of sustained effects of acupuncture. Mechanisms that may underlie these effects have been proposed, including the suggestion that acupuncture works by stimulating the vagal-adrenal axis and reducing inflammation.^26^ A meta-analysis of acupuncture trials for other painful conditions with longer-term follow-up suggested that treatment effects persist after completion of the intervention.^5^ Acupuncture is appealing to some patients because the adverse effects are generally limited compared with medications; however, this treatment is not covered by many insurance plans and is currently only covered for Medicare beneficiaries with low back pain. Ongoing trials are evaluating personalized approaches to acupuncture protocols.^27,28^

### Limitations

Interventions to assess strategies for controlling pain can be challenging to perform and interpret. First, pain is subjective and can vary depending on multiple factors other than the AI medication and the study intervention. Second, there is no accepted single definition of meaningful improvement in pain despite considerable deliberations by experts in the field.^12,13,29^ Third, all studies are methodologically challenged by the placebo effect. The placebo effect is a patient’s response to participation in a therapeutic encounter, including effects related to the clinician and the treatment environment itself.^30^ By using a WC group as well as SA, some of the prior methodologic challenges were accounted for; however, we found a reduction in symptoms in all groups that persisted over time. Although we did not find a difference between the groups in improvement of the functional measures evaluated (Timed Get Up and Go and grip strength), there are also limited data on the effect of AI arthralgias on these specific measures, and most patients did not have functional impairments at baseline.

## Conclusions

In this randomized clinical trial, we found that among postmenopausal women with early breast cancer who experienced AI-related arthralgias, a 12-week intervention of TA compared with SA or WC resulted in statistically significant sustained reduction in joint pain at 52 weeks. This study highlights the durability of the acupuncture response through 1 year, as well as the importance of having both SA and WC groups to fully evaluate the effect of the acupuncture intervention.

## Notes

Supplement 1.
Trial Protocol

Supplement 2. **eFigure.** CONSORT Diagram Showing the Disposition of Patients With Respect to the Examination of the Primary Endpoint, the 6-Week Breast Pain Index-Short Form (BPI-SF) Worst Pain Score **eTable 1.** Observed and Fitted Group Mean Results for Brief Pain Inventory and PROMIS Pain Intensity Scores at Weeks 6, 12, 24, and 52 in Each Group **eTable 2.** Determination of Best Model Fit for Longitudinal Data Analyses **eTable 3.** Grip Strength, Maximal Contraction Over Left and Right Hand, by Randomized Arm and Time Point **eTable 4.** Grip Strength, Change in Maximal Contraction Between Follow-up and Baseline by Randomized Arm and Time Point **eTable 5.** Timed Get Up and Go, by Randomized Arm and Time Point **eTable 6.** Timed Get Up and Go, Change Between Follow-up and Baseline, by Randomized Arm and Time Point

Supplement 3.
Data Sharing Statement

## References

[1] Burstein HJ, Lacchetti C, Anderson H, . Adjuvant endocrine therapy for women with hormone receptor-positive breast cancer: American Society of Clinical Oncology clinical practice guideline update on ovarian suppression. j Clin Oncol. 2016;34(14):1689-1701. doi:10.1200/JCO.2015.65.9573 26884586

[2] Hershman DL, Kushi LH, Shao T, . Early discontinuation and nonadherence to adjuvant hormonal therapy in a cohort of 8,769 early-stage breast cancer patients. J Clin Oncol. 2010;28(27):4120-4128. doi:10.1200/JCO.2009.25.9655 20585090 PMC2953970

[3] Hershman DL, Unger JM, Greenlee H, . Effect of acupuncture vs sham acupuncture or waitlist control on joint pain related to aromatase inhibitors among women with early-stage breast cancer: a randomized clinical trial. JAMA. 2018;320(2):167-176. doi:10.1001/jama.2018.8907 29998338 PMC6583520

[4] Liu X, Lu J, Wang G, . Acupuncture for arthralgia induced by aromatase inhibitors in patients with breast cancer: a systematic review and meta-analysis. Integr Cancer Ther. 2021;20:1534735420980811. doi:10.1177/1534735420980811 33586504 PMC7883140

[5] MacPherson H, Vertosick EA, Foster NE, ; Acupuncture Trialists’ Collaboration. The persistence of the effects of acupuncture after a course of treatment: a meta-analysis of patients with chronic pain. Pain. 2017;158(5):784-793. doi:10.1097/j.pain.0000000000000747 27764035 PMC5393924

[6] Greenlee H, Crew KD, Capodice J, . Randomized sham-controlled pilot trial of weekly electro-acupuncture for the prevention of taxane-induced peripheral neuropathy in women with early stage breast cancer. Breast Cancer Res Treat. 2016;156(3):453-464. doi:10.1007/s10549-016-3759-2 27013473 PMC4924571

[7] Daut RL, Cleeland CS, Flanery RC. Development of the Wisconsin Brief Pain Questionnaire to assess pain in cancer and other diseases. Pain. 1983;17(2):197-210. doi:10.1016/0304-3959(83)90143-4 6646795

[8] Garcia SF, Cella D, Clauser SB, . Standardizing patient-reported outcomes assessment in cancer clinical trials: a patient-reported outcomes measurement information system initiative. J Clin Oncol. 2007;25(32):5106-5112. doi:10.1200/JCO.2007.12.2341 17991929

[9] Fraser A, Vallow J, Preston A, Cooper RG. Predicting ‘normal’ grip strength for rheumatoid arthritis patients. Rheumatology (Oxford). 1999;38(6):521-528. doi:10.1093/rheumatology/38.6.521 10402072

[10] Mathias S, Nayak US, Isaacs B. Balance in elderly patients: the “get-up and go” test. Arch Phys Med Rehabil. 1986;67(6):387-389. 3487300

[11] SAS Institute Inc. SAS 9.4 System Options. 5th ed. SAS Institute Inc; 2016.

[12] Dworkin RH, Turk DC, McDermott MP, . Interpreting the clinical importance of group differences in chronic pain clinical trials: IMMPACT recommendations. Pain. 2009;146(3):238-244. doi:10.1016/j.pain.2009.08.019 19836888

[13] Dworkin RH, Turk DC, Wyrwich KW, . Interpreting the clinical importance of treatment outcomes in chronic pain clinical trials: IMMPACT recommendations. J Pain. 2008;9(2):105-121. doi:10.1016/j.jpain.2007.09.005 18055266

[14] Smith EM, Pang H, Cirrincione C, ; Alliance for Clinical Trials in Oncology. Effect of duloxetine on pain, function, and quality of life among patients with chemotherapy-induced painful peripheral neuropathy: a randomized clinical trial. JAMA. 2013;309(13):1359-1367. doi:10.1001/jama.2013.2813 23549581 PMC3912515

[15] Kroenke K, Theobald D, Wu J, . Effect of telecare management on pain and depression in patients with cancer: a randomized trial. JAMA. 2010;304(2):163-171. doi:10.1001/jama.2010.944 20628129 PMC3010214

[16] Vickers AJ, Vertosick EA, Lewith G, ; Acupuncture Trialists’ Collaboration. Acupuncture for chronic pain: update of an individual patient data meta-analysis. J Pain. 2018;19(5):455-474. doi:10.1016/j.jpain.2017.11.005 29198932 PMC5927830

[17] Henry NL, Unger JM, Schott AF, . Randomized, multicenter, placebo-controlled clinical trial of duloxetine versus placebo for aromatase inhibitor-associated arthralgias in early-stage breast cancer: SWOG S1202. J Clin Oncol.2017;36(4):326-332. doi:10.1200/JCO.2017.74.6651 29136387 PMC5805479

[18] Cathcart-Rake E, Novotny P, Leon-Ferre R, . A randomized, double-blind, placebo-controlled trial of testosterone for treatment of postmenopausal women with aromatase inhibitor-induced arthralgias: Alliance study A221102. Support Care Cancer. 2021;29(1):387-396. doi:10.1007/s00520-020-05473-2 32372176 PMC7644633

[19] Shen S, Unger JM, Crew KD, . Omega-3 fatty acid use for obese breast cancer patients with aromatase inhibitor-related arthralgia (SWOG S0927). Breast Cancer Res Treat. 2018;172(3):603-610. doi:10.1007/s10549-018-4946-0 30159789 PMC6681817

[20] Niravath P, Hilsenbeck SG, Wang T, . Randomized controlled trial of high-dose versus standard-dose vitamin D_3_ for prevention of aromatase inhibitor-induced arthralgia. Breast Cancer Res Treat. 2019;177(2):427-435. doi:10.1007/s10549-019-05319-4 31218477

[21] Irwin ML, Cartmel B, Gross CP, . Randomized exercise trial of aromatase inhibitor-induced arthralgia in breast cancer survivors. J Clin Oncol. 2015;33(10):1104-1111. doi:10.1200/JCO.2014.57.1547 25452437 PMC4372849

[22] Crew KD, Capodice JL, Greenlee H, . Randomized, blinded, sham-controlled trial of acupuncture for the management of aromatase inhibitor-associated joint symptoms in women with early-stage breast cancer. J Clin Oncol. 2010;28(7):1154-1160. doi:10.1200/JCO.2009.23.4708 20100963

[23] Mao JJ, Xie SX, Farrar JT, . A randomised trial of electro-acupuncture for arthralgia related to aromatase inhibitor use. Eur J Cancer. 2014;50(2):267-276. doi:10.1016/j.ejca.2013.09.022 24210070 PMC3972040

[24] Bao T, Cai L, Giles JT, . A dual-center randomized controlled double blind trial assessing the effect of acupuncture in reducing musculoskeletal symptoms in breast cancer patients taking aromatase inhibitors. Breast Cancer Res Treat. 2013;138(1):167-174. doi:10.1007/s10549-013-2427-z 23393007 PMC3594526

[25] Oh B, Kimble B, Costa DS, . Acupuncture for treatment of arthralgia secondary to aromatase inhibitor therapy in women with early breast cancer: pilot study. Acupunct Med. 2013;31(3):264-271. doi:10.1136/acupmed-2012-010309 23722951

[26] Liu S, Wang Z, Su Y, . A neuroanatomical basis for electroacupuncture to drive the vagal-adrenal axis. Nature. 2021;598(7882):641-645. doi:10.1038/s41586-021-04001-4 34646018 PMC9178665

[27] Liou KT, Baser R, Romero SAD, . Personalized electro-acupuncture versus auricular-acupuncture comparative effectiveness (PEACE): a protocol of a randomized controlled trial for chronic musculoskeletal pain in cancer survivors. Medicine (Baltimore). 2020;99(21):e20085. doi:10.1097/MD.0000000000020085 32481275 PMC7249872

[28] Mao JJ, Liou KT, Baser RE, . Effectiveness of electroacupuncture or auricular acupuncture vs usual care for chronic musculoskeletal pain among cancer survivors: the PEACE randomized clinical trial. JAMA Oncol. 2021;7(5):720-727. doi:10.1001/jamaoncol.2021.0310 33734288 PMC7974834

[29] Dworkin RH, Turk DC, Peirce-Sandner S, . Research design considerations for confirmatory chronic pain clinical trials: IMMPACT recommendations. Pain. 2010;149(2):177-193. doi:10.1016/j.pain.2010.02.018 20207481

[30] Finniss DG, Kaptchuk TJ, Miller F, Benedetti F. Biological, clinical, and ethical advances of placebo effects. Lancet. 2010;375(9715):686-695. doi:10.1016/S0140-6736(09)61706-2 20171404 PMC2832199

