# Supplementary material for: Comparison of Acupuncture vs Sham Acupuncture or Waiting List Control in the Treatment of Aromatase Inhibitor–Related Joint Pain: A Randomized Clinical Trial
Source: JAMA Netw Open. 2022 Nov 11;5(11):e2241720. doi: 10.1001/jamanetworkopen.2022.41720 (PMC9652759; doi:10.1001/jamanetworkopen.2022.41720)
Supplement: Supplement 1. — Trial Protocol [file jamanetwopen-e2241720-s001.pdf]

SWOG
Leading cancer research. Together.

GROUP CHAIR'S OFFICE
Laurence H. Baker, DO
CHAIR
24 Frank Lloyd Wright Dr
PO Box 483
Ann Arbor, MI 48106
734-998-7130
734-998-7118 FAX

OPERATIONS OFFICE
4201 Medical Dr
Suite 250
San Antonio, T X 78229
210-614-8808
210-614-0006 FAX

STATISTICAL CENTER
1730 Minor Ave
Suite 1900
Seattle, WA 98101
206-652-2267
206-342-1616 FAX

1100 Fairview Ave North
M3-C102
PO Box 19024
Seattle, WA 98109
206-667-4623
206-667-4408 FAX

swog.org

March 13, 2012

TO: DCP PROTOCOL AND INFORMATION OFFICE

FROM: Kimberly F. Kaberle, Protocol Coordinator

RE: **S1200,** "Randomized Blinded Sham- and Waitlist-Controlled Trial of Acupuncture for Joint Symptoms Related to Aromatase Inhibitors in Women with Early Stage Breast Cancer."

STATUS NOTICE

Study Coordinator: Dawn Hershman, M.D., M.S.
Phone: 212/305-1945
E-mail: dlh23@columbia.edu

ACTIVATION REVISION

Between DCP approval of the protocol referenced above and activation, the following editorial revisions have been made:

1. Section 7.3, Pages 19-20: The first sentence of the first paragraph has been updated to clarify that the Columbia CRA will maintain the acupuncturist-completed web-based study calendar and will document adherence on the **S1200** Adherence to Acupuncture Form (Form #6044). In the last sentence of the last paragraph, “and AI metabolites” has been removed as urine will only be collected during Week 6 and 12 for CTX-II.

2. Section 7.4, Page 21: The **S1200** Adherence to Acupuncture Form number has been corrected from #6944 to #6044. The last sentence in the first paragraph regarding the **S1200** Cover Sheet for Patient Completed Questionnaires has been removed.

3. Section 7.5, Page 21: The Dynamometer weight has been corrected from 176 to 88. Kilopascals (kPa) has been changed to kilograms (kg) throughout the first paragraph. In the second sentence “and the side of the breast surgery and lymph node dissection” has been removed.

4. Section 7.6a, Page 22: The last paragraph has been moved from Section 8.2.

5. Section 9.0, Page 24: Functional testing was added to Week 24 and Week 52 in order to be consistent with the protocol. The “π” symbol had been inadvertently removed and was added above Week 24. An X has been added under Prestudy for the **S1200** Cover Sheet for Patient-Completed Questionnaires and **S1200** BPI-SF. The X under Baseline for the **S1200** BPI-SF has been removed. The Xs have been removed from the Waitlist Control row. An X was added at Week 12 under Physical Exam.

SWOG Authorized Document

6. Section 11.4, Page 28: The interim analysis has been removed from the protocol.

7. Section 14.4, Page 31: The following has been added to the heading of this section: (WITHIN 24 HOURS AFTER OBTAINING SPECIMEN)

8. Section 14.5, Page 31: This section has been combined with Section 14.6. Subsequent sections have been renumbered accordingly.

9. Section 14.7j, Page 32: Data collection during Weeks 24 and 52 has been added for this form, therefore "[WEEK 6 AND 12]" has been removed.

10. Section 14.8, Page 32: The following has been added to the heading of this section: WITHIN 24 HOURS OF OBTAINING SPECIMENS

11. Section 14.9c, Page 32: The **S1200** Cover Sheet for Patient-Completed Questionnaires (Form #19445) has been added.

12. Sections 15.1c.1 and 15.1c.2, Page 33: Specified that blood will be collected in "10 mL" tubes.

13. Section 15.1c.3, Page 33: "till be aliquoted into five 1.8 mL cryovials, and" has been deleted and replaced with "will be". "Promptly frozen" has been replaced with "collected in a 20 ml tube and stored at 4°C for up to 24 hours".

14. Section 15.2b.5, Page 34: The last three sentences were added to include instructions regarding the scheduled telephone assessments.

15. Model Consent Form, Page 53: Under "Who is doing this study?", the following sentence was added: This particular study is also being supported by the National Center for Complementary and Alternative Medicine and by the Office for Research on Women's Health.

16. Model Consent Form, Page 54: Under "Before you begin the study…", the following bullet was added "You will be asked to take one questionnaire that will ask you to rate joint pain and stiffness." Under "Randomization", in the second paragraph it was clarified that patient will wait 24 weeks before "having the choice to receive" true acupuncture.

17. Model Consent Form, Page 57: The acupuncturist has been added to the second paragraph.

18. Model Consent Form, Page 55: In the fourth bullet, the amount of urine collected has been changed from 1 tablespoon to 1/3 of a cup. The following definition of "biomarker" has been added to the third bullet after "specific biomarker": biological molecule found in blood that is a sign of a normal or abnormal process, or of a condition or disease.

19. Model Consent Form, Page 56: In the second bullet, the amount of urine collected has been changed from 1 tablespoon to 1/3 of a cup. The following was added after the 4th sentence of the second bullet: "These results will not be made available to you or your study doctor."

20. Model Consent Form, Page 58: Under "Will my medical information be kept private?", the Office for Research on Women's Health (ORWH) has been added to the 2nd bullet.

SWOG Authorized Document

21. **S1200** Brief Pain Inventory Short Form (BPI-SF) (Form #40963): Week 16 and 20 assessments have been added.

22. **S1200** Aromatase Inhibitor Usage Form (Form #39879): On Page 1 under "Status", removed the note to "submit **S1200** Off Protocol Notice" if the patient has discontinued AI therapy.

23. **S1200** Adverse Event Summary Form (Form #5183): Data collection for Week 24 and Week 52 has been added.

24. **S1200** Supplemental Agents Reporting Form (Form #9413): Week 16 and 20 assessments have been added. On Page 2, changed "Has the patient used therapeutic massage since the last assessment?" to "Has the patient used therapeutic massage for joint symptoms since the last assessment?"; deleted the questions regarding complementary or alternative therapies, and added "Has the patient used any dietary supplement for joint symptoms since the last assessment?".

25. **S1200** Off Protocol Notice (Form #9707): Deleted the following from the "Off Protocol Reason" list: Discontinued aromatase inhibitor therapy.

26. Section 19.1, Pages 96-98: In the ninth sentence of the first paragraph on Page 96, "three" has been replaced with "four" and "joint areas" has been changed to "joints". In the second sentence of the second paragraph, 3 inch acupuncture needles have been added. In the second paragraph, the sixth sentence on accessing joint-specific areas that are not accessible in the supine position has been added. In the seventh sentence of the second paragraph, "with a minimum of two years experience treating cancer patients at each site" has been removed. SI-5 was moved from Page 97 under "Fingers" to Page 98 under "Wrist". In the fifth sentence of the first paragraph under "Description of Sham Acupuncture" on Page 98, "three" has been replaced with "four". In the first sentence of the second paragraph, "Mac™" has been added in front of "single-use" and "with plastic guide tube" has been added after "disposable". In the second sentence, the "25mm" needle size has been replaced with "0.5 inch 34-gauge" and the manufacturer and distribution companies have been updated. The following has been added to the third sentence: inserting the needle by tapping in just to the upper level of the guide tube, at a transverse angle.

27. Section 19.4, Page 102: The requirement of a minimum of two years experience has been removed from the first paragraph.

28. Section 19.6, Pages 104-106: This section has been added to include the procedures for emergency unblinding. This appendix has been added to the list on Page 96.

29. Grammatical and formatting changes were made throughout the protocol, which did not affect content. Typographical errors were corrected as well as pagination.

Enclosed please find a complete copy of the study. Please replace any previous copies with the version dated 3/13/12.

PC/kfk
Enclosure/file copy

SWOG Authorized Document

SWOG

RANDOMIZED BLINDED SHAM- AND WAITLIST-CONTROLLED TRIAL OF ACUPUNCTURE FOR JOINT SYMPTOMS RELATED TO AROMATASE INHIBITORS IN WOMEN WITH EARLY STAGE BREAST CANCER

		Page
	SCHEMA	2
1.0	OBJECTIVES	3
2.0	BACKGROUND	4
3.0	DRUG INFORMATION	13
4.0	STAGING CRITERIA	13
5.0	ELIGIBILITY CRITERIA	17
6.0	STRATIFICATION FACTORS	19
7.0	PROCEDURES AND STUDY PLAN	19
8.0	SAFETY MONITORING	23
9.0	STUDY CALENDAR	24
10.0	CRITERIA FOR EVALUATION AND ENDPOINT DEFINITIONS	25
11.0	STATISTICAL CONSIDERATIONS	26
12.0	DISCIPLINE REVIEW	28
13.0	REGISTRATION GUIDELINES	28
14.0	DATA SUBMISSION SCHEDULE	30
15.0	SPECIAL INSTRUCTIONS	33
16.0	ETHICAL AND REGULATORY CONSIDERATIONS	36
17.0	BIBLIOGRAPHY	39
18.0	MASTER FORMS SET	50
19.0	APPENDIX	65

PARTICIPANTS: Approved Acupuncturists at: Columbia University, Fred Hutchinson Cancer Research Center, Grand Rapids CCOP, Kaiser Permanente Northern California, Oregon Health and Sciences University, University of Southern California

SPONSORS: National Center for Complementary and Alternative Medicine (R01 AT00636-01)
National Cancer Institute, Office of Research on Women's Health

STUDY COORDINATORS:

Dawn Hershman, M.D., M.S. (Medical Oncology)
Columbia University Medical Center
161 Fort Washington Avenue
10-1068
New York, NY 10032
Phone: 212/305-1945
FAX: 212/305-0178
E-mail: dlh23@columbia.edu

Katherine Crew, M.D., M.S. (Medical Oncology)
Columbia University
161 Fort Washington Avenue
10-1072
New York, NY 10032
Phone: 212/305-1732
FAX: 212/305-0178
E-mail: kd59@columbia.edu

BIOSTATISTICIANS:

Joseph Unger, M.S. (Biostatistics)
Danika Lew, M.A.
SWOG Statistical Center
Fred Hutchinson Cancer Research Center
1100 Fairview Avenue North, M3-C102
PO Box 19024
Seattle, WA 98109-1024
Phone: 206/667-4623
FAX: 206/667-4408
E-mail: junger@fhcrc.org
E-mail: dlew@fhcrc.org

mb

SWOG Authorized Document

SCHEMA

Women with stage I-III hormone receptor-positive breast cancer who are receiving adjuvant aromatase inhibitors (AIs) and report worst pain of at least 5 (out of 10) that has started or increased since initiation of aromatase inhibitor treatment

↓

At baseline:
Questionnaires
Functional testing
Blood/urine collection

↓

RANDOMIZE
2:1:1

↓

Intervention*:
True Acupuncture twice weekly x 6 weeks (12 sessions) then weekly x 6 weeks (6 sessions)
Vs.
Sham Acupuncture twice weekly x 6 weeks (12 sessions) then weekly x 6 weeks (6 sessions)
Vs.
Waitlist control x 12 weeks

↓

At 2 and 4 weeks:
Telephone contact

↓

At 6, 12, and 24 weeks:
Questionnaires
Functional testing
Blood/urine collection

↓

At 16 and 20 weeks:
Telephone contact and BPI-SF Questionnaire

↓

At 52 weeks:
Questionnaires
Functional testing
Urine collection

*At 24 weeks, all 3 arms will receive vouchers for 10 true acupuncture sessions.

SWOG Authorized Document

1.0 OBJECTIVES

1.1 Primary objective:

To determine whether true acupuncture administered twice weekly for 6 weeks (8-12 sessions) compared to sham acupuncture and waitlist control causes a significant reduction in joint pain related to aromatase inhibitors (AIs) in women with early stage breast cancer as measured by the Brief Pain Inventory-Short Form (BPI-SF) worst pain score at 6 weeks.

1.2 Secondary objectives are to investigate the effects of true acupuncture administered twice weekly for 6 weeks (8-12 sessions) followed by 6 weekly treatments (4-6 sessions) of maintenance (12-18 sessions total over 12 weeks) compared to sham acupuncture and waitlist control in this study population. The evaluations at 12 and 24 weeks are to determine the benefit of additional 6 weekly acupuncture treatments for maintenance and to determine the durability of response after stopping acupuncture, respectively. The evaluation at 52 weeks is to determine the long-term effects of acupuncture and adherence to AIs.

a. To evaluate the effects of acupuncture on the Brief Pain Inventory-Short Form (BPI-SF) worst pain, worst stiffness, pain severity, and pain-related interference scores at 6, 12, 16, 20, 24, and 52 weeks.

b. To evaluate the effects of acupuncture on Western Ontario and McMaster Universities Osteoarthritis (WOMAC) index (pain, stiffness, and function) for the hips and knees at 6, 12, 24, and 52 weeks.

c. To evaluate the effects of acupuncture on Modified-Score for the Assessment and Quantification of Chronic Rheumatoid Affections of the Hands (M-SACRAH) (pain, stiffness, and function) at 6, 12, 24, and 52 weeks.

d. To evaluate the effects of acupuncture on the PROMIS Pain Impact-Short Form (PROMIS PI-SF) at 6, 12, 24, and 52 weeks.

e. To evaluate the effects of acupuncture on quality of life (QOL) as assessed by the Functional Assessment of Cancer Therapy-Endocrine Subscales (FACT-ES) at 6, 12, 24, and 52 weeks.

f. To evaluate the effects of acupuncture on functional testing with grip strength and “Timed Get Up and Go” (TGUG) test at 6, 12, 24, and 52 weeks.

g. To evaluate the effects of acupuncture on analgesic and opioid use at 2, 4, 6, 12, 16, 20, 24, and 52 weeks.

h. To evaluate the effects of acupuncture on self-reported AI adherence at 12, 24, and 52 weeks.

i. To assess AI adherence via urine AI metabolites at baseline, 24, and 52 weeks.

j. To evaluate the effects of acupuncture on serum hormones (estradiol, FSH, LH) and inflammatory biomarkers (serum TNFα, IL-6, IL-12, CRP and urine CTX-II) at 6, 12, and 24 weeks.

k. To evaluate whether polymorphisms in *CYP19A1* aromatase gene predict severity of AI-related joint symptoms.

l. To assess the safety and tolerability of acupuncture in this study population.

SWOG Authorized Document

2.0 BACKGROUND

Use of Aromatase Inhibitors for Breast Cancer

Endocrine treatments, including tamoxifen and aromatase inhibitors, are widely prescribed for all stages of hormone-responsive breast cancer. These agents are primarily directed at inducing estrogen deprivation through blocking estrogen at the receptor level (tamoxifen), or by inhibiting estrogen biosynthesis (aromatase inhibitors). Tamoxifen, a selective estrogen receptor modulator (SERM), is both an antagonist and a partial agonist of the estrogen receptor. *(1)* Tamoxifen may cause hot flashes, vaginal bleeding, and serious long-term side effects, including endometrial cancer and thromboembolism. *(2-4)*

Third-generation aromatase inhibitors (AIs), namely anastrozole, letrozole and exemestane, markedly suppress plasma estrogen levels in postmenopausal women by inhibiting the enzyme responsible for the conversion of androgens to estrogens in peripheral tissues (skin, muscle, fat, benign and malignant breast tissue). *(5-7)* Treatment of hormone receptor-positive breast cancer in postmenopausal women with third-generation AIs has been shown to be superior to tamoxifen with respect to disease-free survival, distant and local recurrence rates, and incidence of contralateral breast cancer. *(8-13)* The Arimidex® (anastrozole), Tamoxifen, Alone or in Combination (ATAC) trial compared five years of anastrozole to 5 years of tamoxifen in 9,366 postmenopausal women with localized breast cancer. After a median follow-up of 68 months, anastrozole significantly prolonged disease-free survival (HR 0.87, 95% CI 0.78-0.97), time-to-recurrence (HR 0.79, 95% CI 0.70-0.90), and contralateral breast cancers (42% reduction, 95% CI 12-62%). *(10)* As a result of these studies, they are currently the first-line hormonal therapy in postmenopausal women with early stage breast cancer and standard of care for women who have received 2 ½ years to 5 years of tamoxifen in the adjuvant settings. *(11-13)* They are also undergoing evaluation as chemopreventive agents in several large clinical trials for the primary prevention of breast cancer.

Side Effects Associated with Aromatase Inhibitors

Despite the well-proven efficacy of AIs for the treatment of hormone-sensitive breast cancer, some patients suffer from side effects or even stop treatment early due to undesirable toxicities. The most common side effects of AIs are hot flashes, vaginal dryness, musculoskeletal pain and headache, and possibly alterations in serum lipid profiles. *(8)* In addition, all third-generation AIs increase bone resorption and may predispose to osteoporosis and fractures. *(14, 15)* In one study, 16% of metastatic breast cancer patients complained of joint pain within 2 months of starting anastrozole and 5% had to discontinue therapy because of severe arthralgia. *(16)* Discontinuation of anastrozole resulted in resolution of pain-related symptoms. In large adjuvant trials involving AIs, the incidence of musculoskeletal disorders was 20% to 30% and nearly 5% of patients discontinued therapy in the AI group because of toxic effects. *(8, 9)* Investigators have recently shown that at the end of 4.5 years of therapy, 30% of women have discontinued hormonal treatment, and of those who continue, only 60% are taking the medication 80% of the time or more. *(17)*

A survey was conducted to assess the prevalence of joint symptoms in postmenopausal women on adjuvant AIs for early stage breast cancer. *(18)* In the clinical breast oncology academic practice at Columbia University Medical Center (CUMC), 253 consecutive breast cancer patients receiving adjuvant AIs were screened over a 4-month period. Of these women, 200 completed a 25-item self-administered survey asking about the presence of joint pain or stiffness in the preceding week, if symptoms started or worsened after initiating AIs, and the location and severity of symptoms using a 0-10 scale. Among all participants, 50% reported joint pain and 47% joint stiffness within the past week which started or worsened after initiating AIs. Among those with AI-related arthralgias, over 50% reported joint symptoms in the hands and/or knees. The score for severity of joint symptoms on a scale of 0-10 was ≥ 5 in 68% of the patients who experienced AI-

SWOG Authorized Document

related arthralgia. Subsequently, 53 patients who were initiating AI therapy were prospectively evaluated. Compared to baseline, there was a 49% increase in joint pain severity at 6 months and an 86% increase in pain-related interference at 6 months as assessed by the Brief Pain Inventory-Short Form (BPI-SF). At 3 months, 46% of subjects had at least a 2-point increase in the BPI-SF worst pain score (0-10 scale) from baseline, and at 6 months, a significant decrease in pinch grip strength was detected (p=0.05). *(19)* The results are consistent with findings from other studies. *(16, 20)*

In a study evaluating menopausal symptoms in breast cancer patients receiving endocrine therapy, there was a significant change in musculoskeletal pain in women receiving AIs. *(21)* Fifty percent of women who were asymptomatic at baseline reported variable degrees of pain after one month of treatment. There was a marked increase in the number of patients reporting severe to intolerable symptoms after three months of therapy, which led to treatment interruption in 11% of patients. Patients usually present with polyarthralgia affecting the hands, knees, hips, lower back or shoulders, which is often refractory to conventional pharmacological interventions. *(16)* The musculoskeletal pain appears to be specific for this class of compounds, regardless of the AI prescribed.

Recent studies have found that AI-induced arthralgia is accompanied by physical changes in the affected joints seen on MRI, EMG, and ultrasound. Women on AIs for six months had decreased grip strength and increased tenosynovial changes seen on MRI. *(22)* In a study conducted by Dizdar et al., women taking AIs have increased tendon thickness and higher rates of effusions in hand joints/tendons on musculoskeletal sonography compared to women who never received AIs. *(23)* AI use is also associated with a greater incidence of carpal tunnel syndrome of moderate intensity and short duration. *(24)*

The risk factors for developing AI-associated arthralgia are unclear. In the ATAC trial, high body mass index (BMI), prior chemotherapy, history of hormone replacement therapy were major risk factors for developing joint symptoms. *(25)* In a cross-sectional survey conducted at CUMC, prior taxane chemotherapy was associated with a 4-fold increased risk of AI-related joint symptoms, and BMI of 25-30 kg/m^2 and prior tamoxifen use were inversely associated with AI-associated arthralgias. *(18)* A recent study found a significant association between greater joint symptoms and lower bone mineral density in women taking AIs. *(26)* Recent studies have reported an association between a polymorphism in the 3' untranslated region of the *CYP19A1* aromatase gene and improved AI treatment efficacy. Less is known about its relationship to toxicity. *(27, 28)*

AIs are better tolerated than tamoxifen with respect to hot flushes, vaginal bleeding and discharge, endometrial cancer and thromboembolic events. *(8)* However, patients receiving AIs have a higher incidence of osteoporosis, fractures and musculoskeletal symptoms (arthralgia and other joint disorders) compared to tamoxifen. *(8, 9, 13)* Although joint pain is not considered a serious side effect, increasing intensity decreases patient compliance and adversely affects quality of life. The risk of important long-term toxicities, including arthritis and osteoporosis, may increase with the use of AIs and the increase in life expectancy among breast cancer survivors. While bisphosphonates such as alendronate are available for the prevention of bone loss, safe and effective treatments are needed to alleviate AI-induced musculoskeletal pain.

Mechanism of Aromatase Inhibitor-Induced Musculoskeletal Pain

Estrogen deficiency after menopause has been linked to an increase in several chronic inflammatory conditions, including osteoporosis and osteoarthritis. *(29, 30)* In terms of preclinical evidence, conjugated equine estrogen demonstrated significant anti-inflammatory activity in a rat model. *(31)* Several investigators have reported the presence of estrogen receptors in cartilage. *(32-34)* When adult monkeys were treated with conjugated equine estrogens, chondrocytes incorporated higher levels of sulfate in proteoglycans compared to baseline. *(35)* Decreased estrogen results in increased release of proinflammatory cytokines from monocytes and

SWOG Authorized Document

macrophages. *(36)* The proinflammatory cytokines, interleukin-1 (IL-1) and tumor necrosis factor (TNFα) promote cartilage reabsorption, inhibit synthesis of proteoglycans and cause inflammation. *(37-39)* From these preclinical results, it seems clear that close interactions exist between estrogen and inflammation as well as cartilage metabolism.

The identification of the two estrogen receptors α and β in human articular chondrocytes provided further evidence that cartilage is sensitive to estrogens. *(32, 40-42)* Various animal and *in vitro* studies suggest that estrogen may play a role in the regulation of cartilage turnover and development of joint disease. In an experimental model of postmenopausal osteoarthritis with ovariectomized rats, estrogen deficiency accelerated cartilage turnover and increased cartilage surface erosion, whereas administration of estrogen or SERMs significantly suppressed cartilage degradation. *(43)* In ovariectomized rats, estrogen prevented the cartilage breakdown caused by IL-6 *(44),* a proinflammatory cytokine that plays a critical role in the pathogenesis of osteoarthritis (OA). *(45)* In a sheep model, estrogen replacement therapy reduced the loss of proteoglycans from cartilage adjacent to OA lesions, providing evidence for a chondroprotective effect of estrogen. *(46)*

Estrogen can influence chondrocyte formation on multiple levels by interacting with cellular growth factors, adhesion molecules, and cytokines. In vitro studies showed a dose-dependent change in matrix protein turnover when cultured chondrocytes were exposed to estradiol. *(33, 34, 47, 48)* Production of IL-6 and type II collagen in articular chondrocytes was affected by estradiol, suggesting possible mechanisms whereby it may affect cartilage metabolism. *(49, 50)* In addition, estrogens may decrease the acceleration in subchondral bone remodeling, which is a key factor in the pathophysiology of osteoarthritis. *(51)*

Several small studies have also evaluated the influence AI therapy has on inflammatory serum markers. Results evaluating CRP, IL-6, and TNFα have been inconsistent. *(52-54)* Even in studies showing AI-related changes to inflammatory markers, such as CRP, these changes did not correlate with symptoms of AI-induced arthralgia. *(53)* One study involving 57 women on AIs found an increase in ANA and anti-DNA positivity rates: 40% vs 19% for ANA and 14% vs 0% for anti-DNA. *(54)* A case-control study evaluating 24 women with AI-related arthralgia and 30 without, were evaluated for a wide range of inflammatory markers. While many of the assayed factors including interferons, interleukins, matrix metalloproteinases (MMPs), and chemokines were different at baseline, these differences did not persist at 6 months. This suggests a possible inflammatory phenotype that predicts the development of this toxicity. *(55)* These studies were limited by small samples sizes.

The exact mechanism of AI-related arthralgia is unclear, but is thought to be related to estrogen deprivation. The effects of estrogen on bone, endometrium, and breast tissue have been extensively studied. Cartilage is not generally viewed as an estrogen responsive tissue, however, several epidemiological studies and a few intervention studies support that estrogen may have a role in osteoarthritis. OA is a disease of joints, involving both cartilage and bone. Progressive and permanent articular cartilage degeneration is the hallmark of osteoarthritis. *(56)* Two biochemical markers, serum cartilage oligomeric matrix protein (COMP) and urine crosslinked C-telopeptides of Type II collagen (CTX-II) were shown to correlate with symptoms and x-ray findings of knee OA. *(57)* Urinary CTX-II, a biochemical marker of type II collagen breakdown, has been negatively correlated with joint surface area and was an important predictor of joint damage assessed by radiographs. *(58)* Factors influencing the incidence of radiological osteoarthritis include obesity and female gender, especially after entering the menopause. *(59, 60)* Over 80 years ago, Cecil and Archer first described “arthritis of the menopause” as the rapid development of hand and knee osteoarthritis coinciding with cessation of menses. *(61)* Various studies report larger increases in women than in men in the incidence and prevalence of hip, knee, and hand osteoarthritis after 50 years of age. *(62)* Sowers et al. reported significant associations of lower serum estradiol and urinary metabolites with the development of knee osteoarthritis in women. *(63)* However, other studies have not found a consistent link between osteoarthritis and sex hormone levels. *(64-66)*

SWOG Authorized Document

Findings in epidemiologic studies regarding a correlation between estrogen and osteoarthritis are inconsistent. *(67)* However, observational studies of the incidence and prevalence of osteoarthritis in postmenopausal women with and without hormone replacement therapy (HRT) have supported a protective effect of estrogens in osteoarthritis. *(62, 68, 69)* In a cross-sectional study by Nevitt et al., women taking HRT had a lower relative risk of developing hip osteoarthritis (OR 0.62; 95% CI 0.49-0.86) compared to women not taking HRT. The protective effect was greater in women on HRT for more than ten years with OR 0.57 (95% CI 0.40-0.82). *(70)* Two studies found that HRT tended to reduce the incidence of radiological knee osteoarthritis. *(71)* In a study of women without osteoarthritis, HRT taken for longer than 5 years was associated with a larger volume of tibial cartilage as compared to women who were not taking HRT. *(56)* The number of years since menopause was more strongly related to diminished knee cartilage volume than age per se, suggesting an effect of hormonal status on knee cartilage. In a randomized controlled trial of HRT, the Heart and Estrogen/progestin Replacement Study (HERS) found no difference in the prevalence of knee pain with or without HRT (24.1% and 26.1%, respectively). *(70)* However, the Women's Health Initiative intervention trial of HRT recently reported a difference in the incidence of any joint pain or swelling in postmenopausal women on estrogen compared to those who were not on estrogen (70.6% versus 77.2%, p=0.01). *(72)*

In elderly postmenopausal women, estrogen may be an important regulator of osteoarthritis. The long-term effects of profound estrogen suppression in breast cancer patients taking AIs are unknown. The acute and long-term side effects of AIs are becoming an increasingly important issue as more and more women are being treated with these agents. Musculoskeletal pain may be associated with a deterioration of quality of life due to physical disability, sleep disturbance, impaired cognitive function, depression and anxiety, and increased utilization of health care resources. *(73-75)* Therefore, targeted interventions that relieve AI-induced musculoskeletal pain are needed.

<u>Current Treatment for Aromatase Inhibitor-Induced Joint Pain</u>

Non-steroidal anti-inflammatory drugs (NSAIDs), the most common medications used to treat pain associated with arthritis, may lead to serious renal and gastrointestinal toxicity, particularly in older adults. *(76, 77)* Selective cyclooxygenase-2 (COX-2) inhibitors, such as rofecoxib and celecoxib, are effective analgesics with fewer gastrointestinal side effects but have recently been shown to elevate the risk of cardiovascular events. *(78)* Non-toxic treatments that effectively relieve AI-induced musculoskeletal pain are needed. Ongoing studies are evaluating the potential benefits of physical activity, vitamin D, glucosamine/chondroitin and omega-3 fatty acids for treating this toxicity.

Since research in this area is limited, a small pilot study evaluating the use of acupuncture to relieve symptoms of AI-associated arthralgias was initially conducted. In this study of 21 women treated with a 6-week course of total body and auricular acupuncture, improvements were reported in pain severity, pain-related functional outcomes, and physical well-being, and no significant adverse events were reported. *(79)* This study was limited due to its small sample size and lack of an adequate control group. Investigators then went on to conduct a randomized, blinded, sham-controlled study in 38 breast cancer patients with AI-associated arthralgias. *(80)* True acupuncture for 6 weeks was associated with about a 50% decrease in mean BPI-SF scores compared to sham acupuncture with superficial needling at non-acupuncture points. Similar findings were seen for the WOMAC and M-SACRAH scores. Results found that acupuncture is an effective and well-tolerated strategy for managing this common treatment-related side effect.

<u>Acupuncture for the Treatment of Joint Pain</u>

Approximately 25% of the population over the age of 55 years are affected by joint pain and about half of them will have some restriction of normal daily activities. *(59, 81)* Virtually all individuals over the age of 65 have radiographic evidence of osteoarthritis. *(82)* With

SWOG Authorized Document

conventional approaches such as analgesics and exercise, controlling pain and minimizing loss of function are the principal aims of treatment. *(83, 84)* About two-thirds of individuals who suffer from joint pain have used complementary and alternative treatments to control their symptoms. *(85)* However, due to the dearth of rigorously derived experimental evidence, most third party payers do not reimburse patients for the cost of many of these procedures.

Acupuncture is a traditional Chinese method of medical treatment involving the insertion of fine, single-use, sterile needles in acupoints according to a system of channels and meridians that was developed by early practitioners of Traditional Chinese Medicine (TCM) over 2000 years ago. The needles are stimulated by either manual manipulation, electrical stimulation, or heat. *(86)* Patients in pain have imbalance in their Qi or vital energy. The Qi travels through 12 main meridians in the body, connecting the body in a weblike matrix. *(87)* Within the traditional framework, meridians are thought to represent conduits for electrical signals. *(88)* During traditional acupuncture, needles are inserted into a meridian point and then manipulated to elicit "de qi." *De qi* has been described as a sensation of achiness, soreness, or dull pain. According to TCM, elicitation of *de qi* re-establishes normal flow of Qi and is essential for clinical efficacy. More recently, it has been postulated that sensation of *de qi* represents activation of nerve receptors during insertion of acupuncture needles at the appropriate location and depth of traditional acupuncture points. *(89)* Currently, acupuncture is often used with TCM and it is a recognized health profession with strict licensure and regulatory status in 40 states. *(90)*

Acupuncture is a popular non-pharmacological modality used for treating a variety of conditions, including musculoskeletal pain. Acupuncture has been shown to have short-term analgesic effect in musculoskeletal pain. *(91, 92)* Clinical trials have found a benefit to patients with knee osteoarthritis when acupuncture is used as an adjunct to conventional management strategies. *(93)* In a randomized study of 97 patients with osteoarthritis of the knee, acupuncture as a complementary therapy to pharmacological treatments is more effective than pharmacological treatment alone, in terms of reducing pain, improving physical function and health-related quality of life. *(94)* A trial conducted in Germany concluded that true acupuncture has a better effect than sham acupuncture in the treatment of knee and back pain. *(95)* However, in two large multicenter trials of acupuncture for osteoarthritis of the knee, one found a benefit in joint pain and function compared to sham acupuncture and the other found no additional improvement in pain scores. *(96,97)*

Acupuncture and TCM vary slightly from Western biomedicine in the methods of physical examination, evaluation and diagnostic criteria. For example, the major diagnostic criteria of TCM practitioners include inquiry, inspection of the body, acupuncture channels or meridians, and the tongue, as well as palpation of the channels, points and pulse. Pulse palpation in TCM occurs at the radial artery in the wrist and is assessed for rate, rhythm, force and shape, which all correspond to internal physiological processes according to TCM theory. Using these criteria, a differential diagnosis is made and an appropriate point prescription is applied as treatment.

Acupuncture points are located on the body using anatomical locations and the TCM standard unit of body measurement termed the "cun." As everyone's body has different dimensions, a cun is defined according to the person whose body is to be treated. For example, 1 cun is equivalent to the width of the thumb, in the middle, at the crease; 3 cun is equivalent to the combined breadth of the 4 fingers, at the level of the pinky finger's first joint above the palm of the hand; and 12 cun is equivalent to the distance from the elbow crease to the wrist crease.

Auricular acupuncture therapy has long been used in China to treat conditions by stimulating certain points on the ear with needles. The history of its use has been documented in various historical texts and auricular therapy is maintained in common use in the present day system of acupuncture. The National Acupuncture Detoxification Association (NADA) protocol has been extensively studied for its effect on addiction and stress relief. This protocol is endorsed by the National Institute on Drug Abuse (NIDA), the governing body of the treatment of chemical dependency, and is being used in some states such as Florida and California within their drug rehabilitation and court systems. *(98-100)* The function of the NADA protocol with full body acupuncture treatment is to relieve pain and decrease stress.

SWOG Authorized Document

The most common presentation of musculoskeletal pain according to TCM is "Bi Syndrome." "Bi" means obstruction or blockage. "Bi Syndrome" describes a set of patterns in which obstruction of qi and blood through the channels causes pain. Qi and blood are defined as the basic elements of all physicological activity according to TCM. The TCM differential diagnosis of "Bi syndrome" encompasses several disorders known in Western biomedicine, such as osteoarthritis, rheumatoid arthritis, rheumatic fever, fibromyalgia, gout and sciatica. *(101-103)* Of interest to this study, AI-induced musculoskeletal pain follows the pattern diagnosis of "Bi syndrome."

The use of complementary and alternative medicine (CAM) is growing in the U.S. According to a Center for Disease Control (CDC) report of the 2002 National Health Interview Study, 75% of respondents reported using some form of CAM. *(104)* Breast cancer patients are the cancer group most likely to use CAM therapies in conjunction with their conventional treatment. *(105-107)* Therapies used include nutritional and herbal supplements, massage, dietary changes, prayer, yoga and acupuncture. *(108-110)* An increasing amount of evidence suggests that acupuncture has a role in the supportive care of cancer patients for treating chemotherapy-induced nausea and vomiting, xerostomia, leukopenia, and other chemoradiotherapy-induced symptoms. *(111-116)* A recent study found that acupuncture treatments given twice weekly for 4 weeks, then weekly for 8 weeks (12 weeks total) was as effective as the antidepressant, venlafaxine, for managing vasomotor symptoms related to hormonal therapy in breast cancer patients. *(117)* In fact, acupuncture had a more durable effect with fewer side effects compared to venlafaxine and had additional benefits, including increased sex drive, improved energy and sense of well-being.

<u>Mechanism of Action of Acupuncture</u>

Ideally, relief from AI-associated arthralgia should be through non-hormonal mechanisms, so as not to interfere with the efficacy of the drug. Acupuncture is under current investigation for its mechanistic effect within the biomedical research paradigm and its influence on particular conditions or symptom sets. Although the precise mechanism of action is unknown, acupuncture analgesia is thought to be mediated by central mechanisms of pain control through the release of specific neurotransmitters, such as endorphin. *(118-120)* A recent study suggested that acupuncture modulates pain signals in the central nervous system at the level of the spinal cord, midbrain and cortex via release of endogenous opiates and neurotransmitters. *(121, 122)* Early studies show that naloxone, an antagonist to opiates, attenuated the analgesic effects of acupuncture in humans and mice. *(120, 123)* In humans after receiving acupuncture, elevated levels of ß endorphin in the cerebrospinal fluid have been observed. *(119)* Animal studies with electro-acupuncture (EA) have shown that EA at low frequency of 2 Hz stimulates the release of ß endorphins, enkelphalin and endomorphin, which activates μ and δ opiate receptors. *(124)* In addition, EA at higher frequency stimulates release of dynorphin which activates the κ opiate receptors. *(125)*

Another proposed mechanism of acupuncture analgesia involves the serotoninergic pathway. Acupuncture has been shown to activate the release of serotonin from the serotoninergic raphe spinal neurons in the nucleus raphemagnus, which is associated with the descending pain inhibitory pathway. *(126)* Serotonin then binds serotonin receptors on inhibitory interneurons in the spinal cord to release enkelphalin, which inhibits pain sensation signals. *(127)* A more recent study in rats found that acupuncture activate neurons in the periductal gray matter of the midbrain, a region in the brain that contributes to the descending pain inhibitory pathway. EA also had an anti-nociceptive effect by abolishing histamine and dopamine release induced by pain stimuli. *(128)* More recently, researchers are looking at the anti-hyperalgesic effect of acupuncture in inflammatory mouse models. In one *in vivo* study employing a persistent inflammatory pain rat model, administration of EA increased paw withdrawal latency suggestive of anti-hyperalgesic effect. In addition, EA was able to reduce inflammatory paw edema in the rats. *(129, 130)*

SWOG Authorized Document

Sham Acupuncture Interventions

The optimal control group for studies of acupuncture is controversial. Sham acupuncture methods include acupuncture for an unrelated condition, needle insertion at nonacupoint locations, noninsertive simulated acupuncture, or use of retractable needles. However, most acupuncture controls are not completely inert. Potential benefits may be conferred from the physiologic effect of needling even when not performed according to established principles. *(131-134)* Detrimental effects from sham needling are also possible, and other studies have found no benefit in the sham group. *(134)*

In the prior sham-controlled study of acupuncture for AI-related joint symptoms, the sham intervention used superficial needle insertion at body locations not recognized as true acupoints. *(80)* Results showed that 22% of subjects who received sham acupuncture reported at least a 2-point decrease in BPI-SF worst pain scores, consistent with the literature that reports a 25-55% placebo effect for nonacupoint and superficial insertion sham methods. *(135)* In this study, 57% of patients in the sham group believed they were receiving true acupuncture, suggesting that blinding was effective. For the auricular acupuncture, the sham intervention will involve adding adhesives to the selected points on the helix of the auricle. While a sham auricular intervention was not applied in the prior study, investigators feel that application of the sham auricular intervention may increase the effectiveness of blinding. While future studies may attempt to look at other point protocols and acupuncture interventions oriented in other traditional theories, investigators feel that it is important to remain consistent in this study design. Moreover, investigators will control for a placebo effect based on previous research as reflected in the statistical methods.

Patient Reported Outcomes for Joint Symptoms

This study will rely on a patient-reported outcome (PRO) for the primary endpoint. Traditionally, the Food and Drug Administration (FDA) requires substantial evidence for reaching a conclusion that a drug or intervention will have an effect on a given condition. Evidence that shows not only a change in symptoms, but how that change translates to other specific endpoints, such as the patients' ability to perform activities, is sometimes required by the FDA for approval. *(136)* The PRO of symptomatic and functional improvement may benefit from the use of objective measures, such as magnetic resonance imaging (MRI) of joints to assess for structural changes or functional assessments, including grip strength. *(22)*

Studies assessing AI-induced arthralgias have shown a correlation between PROs and objective findings. Morales et al. demonstrated that the subjective symptoms of AI-induced arthralgias in the hands are associated with physiologic changes to the joint and functional impairments. *(22)* The patients in this study were assessed with serial MRIs of the hands, measurements of grip strength, and symptoms self-assessments. In a 6-month period, women taking AIs were more likely than those on tamoxifen to have an increase in tenosynovial changes as seen on MRI, a decrease in grip strength as measured by a sphygmomanometer, as well as increased pain and stiffness as measured by self-administered questionnaire. *(22)*

Since there are no well-validated measures specifically for AI-induced arthralgias, scales that captured joint pain, stiffness, and functional status in the hips and knees (WOMAC) and hands (M-SACRAH) were selected, and a general pain scale used in cancer patients (BPI-SF). The BPI-SF *(137)* is a 14-item questionnaire which asks patients to rate pain over the prior week and the degree to which it interferes with activities on a 0 to 10 scale. Numeric rating scales such as the BPI are among the most common, valid, and reliable measures used to assess cancer pain. *(138)* The Western Ontario and McMaster Universities Osteoarthritis Index (WOMAC) Version 3.1 is a validated measure for assessing osteoarthritis of the knees or hips and consists of 24 questions related to three subscales: pain, stiffness, and physical function. *(139)* The Modified-Score for the Assessment and Quantification of Chronic Rheumatoid Affections of the Hands (M-SACRAH) consists of three domains assessing pain, stiffness, and functional status in patients suffering

SWOG Authorized Document

from hand osteoarthritis and rheumatoid arthritis. *(140)* Well-validated quantitative functional testing with grip strength to assess hand function and the "Timed Get Up and Go" (TGUG) test to assess functional status in the lower extremities have also been included. *(141,142)*

Acupuncture for Aromatase Inhibitor-Related Arthralgias

Researchers at CUMC have extensive experience evaluating interventions for the treatment of AI-induced arthralgias. Since research in this area is limited, a small pilot study evaluating the use of acupuncture to relieve symptoms of AI-associated arthralgias was initially conducted. In this study of 21 women treated with full body and auricular acupuncture twice weekly for six weeks, improvements were reported in pain severity, pain-related functional outcomes, and physical well-being, and no significant adverse events were reported. *(79)* Another pilot study used electro-acupuncture administered twice weekly for 2 weeks followed by 6 weekly treatments in 12 breast cancer survivors with AI-related arthralgia. *(143)* Similarly, the patients reported significant reductions in pain severity, stiffness, and joint symptom interference according to the BPI. Subjects also reported a significant decrease in fatigue and anxiety. These studies were limited due to small sample sizes, unblinded interventions, and lack of an adequate control group.

A randomized, blinded, sham-controlled study in 38 women with early stage breast cancer and AI-induced arthralgias to assess the efficacy of acupuncture was then conducted. As is shown in Figure 1, a 6-week course of true acupuncture was associated with about a 50% decrease in mean BPI-SF scores. No change from baseline was observed for the sham arm. At 6 weeks, the mean BPI-SF worst pain scores (range, 0-10) were lower for true acupuncture compared to sham acupuncture (3.0 vs. 5.5, p<0.001), as well as pain severity (2.6 vs. 4.5, p=0.003) and pain-related interference (2.5 vs. 4.5, p=0.002). Similar findings were seen for the WOMAC and M-SACRAH scores which assess knee/hip and hand joint symptoms, respectively. *(144)* These measures were very effective at capturing the symptoms of AI-related arthralgia.

Figure 1. Percent change in the group mean Brief Pain Inventory-Short Form (BPI-SF) scores from baseline to 3 and 6 weeks for the true and sham acupuncture groups: A) BPI-SF worst pain, B) BPI-SF pain severity, and C) BPI-SF pain-related interference.

A. B. C.

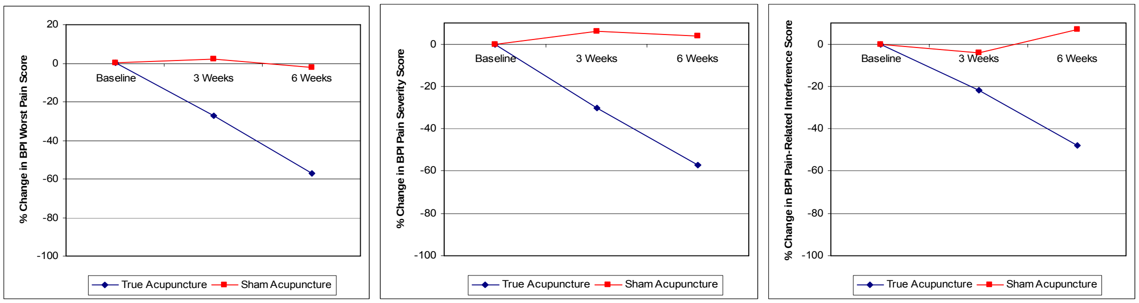

Summary

Due to early detection and improved treatments, women with breast cancer are living longer. The increase in breast cancer survival is largely due to the benefits of hormonal therapy, such as tamoxifen and aromatase inhibitors (AIs), for the treatment of hormone-sensitive breast cancer. Recent clinical trials have demonstrated that AIs are more effective than tamoxifen at reducing breast cancer recurrences. *(8-13)* However, breast cancer patients receiving AIs have a higher incidence of osteoporosis, bone fractures and musculoskeletal symptoms, particularly joint pain and stiffness. Musculoskeletal pain, which occurs in up to 50% of patients treated with AIs *(18),* may lead to noncompliance, deterioration of quality of life due to physical disability, sleep disturbance, impaired cognitive function, depression and anxiety, and increased utilization of health care resources. *(73-75)* Since women with hormone receptor-positive breast cancer require long-term hormonal therapy, an important objective is minimizing long-term side effects to enhance patient compliance and improve quality of life. Therefore, targeted interventions that relieve AI-induced musculoskeletal pain are needed.

SWOG Authorized Document

There are currently no proven treatments for AI-induced arthralgias. Acupuncture is a popular non-pharmacologic modality that has been shown to have analgesic effects in many chronic pain syndromes, including osteoarthritis. A recent evaluation of acupuncture by the National Institutes of Health concluded that it is a useful adjunct in a range of painful conditions, including musculoskeletal pain. *(145)* While the use of complementary and alternative medicine for the treatment of persistent musculoskeletal pain continues to increase, rigorous clinical trials examining their efficacy are needed before definitive recommendations can be made. Investigators recently conducted a randomized, blinded, sham-controlled trial to assess the effect of acupuncture on musculoskeletal pain induced by AIs. The true acupuncture group had about a 50% decrease in mean BPI-SF worst pain score compared to the sham acupuncture group (3.0 vs 5.5, $p<0.001$). *(80)* There were also differences in pain severity and pain related interference. Acupuncture was well tolerated with no complications. However, despite these promising results, this single institution study had a relatively small sample size, had a single acupuncturist, and was only a 6-week intervention. *(80)* For the community, cost and lack of insurance coverage limit access to this therapy. A larger, confirmatory, multicenter trial is necessary to truly control for confounding factors, and may provide the data critical to extend coverage for this indication. This approach is innovative because it is a new application of a well recognized pain therapy for breast cancer supportive care. Improved adherence is critical for this class of medications, given the large discontinuation rate due to toxicity, which has been shown to affect survival. *(17)*

For this current proposal, SWOG will conduct a multicenter randomized, blinded, sham- and waitlist-controlled clinical trial to test the effect of acupuncture on AI-associated joint pain in 228 postmenopausal women with early stage hormone receptor-positive breast cancer who report joint pain that has increased or started since initiation of AI therapy. The primary endpoints are to compare the mean BPI-SF worst pain scores at 6 weeks in the true acupuncture compared to sham acupuncture and waitlist groups in order to confirm initial results of a 6-week acupuncture intervention given twice weekly in a larger multicenter study. Secondary exploratory endpoints include evaluations at 12, 24, and 52 weeks to determine the benefit of additional 6 weekly acupuncture treatments for maintenance and to determine the durability of response 12 and 40 weeks after stopping acupuncture, respectively. This study will be the first large multicenter trial to investigate the effect of acupuncture in treating AI-induced joint symptoms in breast cancer patients. This is a common complaint in this patient population which can lead to the discontinuation of a life-saving therapy. Research has shown that even taking this medication less than 80% of the time can have a negative impact on survival. *(17)* Results from this study will inform clinicians, insurance companies and patients on whether or not this is an effective therapy, and may help predict who is most likely to benefit from this therapy.

Inclusion of Minorities

This clinical trial was designed to include minorities, but was not designed to measure differences of intervention in minority subgroups. It is expected that the patients accrued to this trial will reflect the minority representation in the local population of the participating sites. Men with breast cancer are not eligible for this study because it is not standard for them to receive aromatase inhibitors. Anticipated accrual to this study by race and ethnicity, based on previous Group trials in this disease type, follows:

SWOG Authorized Document

Ethnic Category	Females	Total
Hispanic or Latino	18	18
Not Hispanic or Latino	210	210
**Total Ethnic**	228	228
**Racial Category**		
American Indian or Alaskan Native	2	2
Asian	5	5
Black or African American	18	18
Native Hawaiian or other Pacific Islander	2	2
White	201	201
**Racial Category: Total of all Subjects**	228	228

3.0 DRUG INFORMATION

There are no drugs used in this study.

4.0 STAGING CRITERIA

4.1 Breast Cancer Staging Criteria, AJCC 7th Edition, 2010

DEFINITION OF TNM

Primary Tumor (T)

Definitions for classifying the primary tumor (T) are the same for clinical and for pathologic classification. If the measurement is made by physical examination, the examiner will use the major headings (T1, T2 or T3). If other measurements, such as mammographic or pathologic measurements are used, the subsets of T1 can be used. Tumors should be measured to the nearest 0.1 cm increment.

TX Primary tumor cannot be assessed
T0 No evidence of primary tumor
Tis Carcinoma in situ
Tis (DCIS) Ductal carcinoma in situ
Tis (LCIS) Lobular carcinoma in situ
Tis (Paget's) Paget's disease of the nipple with no tumor

NOTE: Paget's disease associated with a tumor is classified according to the size of the tumor.

SWOG Authorized Document

T1	Tumor 2 cm or less in greatest dimension
T1mic	Microinvasion 0.1 cm or less in greatest dimension
T1a	Tumor more than 0.1 cm but no more than 0.5 cm in greatest dimension
T1b	Tumor more than 0.5 cm but not more than 1 cm in greatest dimension
T1c	Tumor more than 1 cm but not more than 2 cm in greatest dimension
T2	Tumor more than 2 cm but not more than 5 cm in greatest dimension
T3	Tumor more than 5 cm in greatest dimension
T4	Tumor of any size with direct extension to (a) chest wall or (b) skin, only as described below:
T4a	Extension to chest wall, not including pectoralis muscle
T4b	Edema (including peau d'orange) or ulceration of the skin of the breast, or satellite skin nodules confined to the same breast
T4c	Both T4a and T4b
T4d	Inflammatory carcinoma

Regional Lymph Nodes (N)

Clinical

NX	Regional lympnodes cannot be assessed
N0	No regional lymph node metastasis
N1	Metastasis to movable ipsilateral axillary lymph node(s)
N2	Metastasis in ipsilateral axillary lymph nodes fixed or matted, or in clinically apparent* ipsilateral internal mammary nodes in the *absence* of clinically evident axillary lymph node metastasis
N2a	Metastasis in ipsilateral axillary lymph nodes fixed to one another (matted) or to other structures
N2b	Metastasis only in clinically detected* ipsilateral internal mammary nodes and in the *absence* of clinically evident axillary lymph node metastasis
N3	Metastasis in ipsilateral infraclavicular lymph node(s) with or without axillary lymph node involvement, or in clinically detected* ipsilateral internal mammary lymph node(s) and in the *presence* of clinically evident axillary lymph node metastasis; or metastasis in ipsilateral supraclavicular lymph node(s) with or without axillary or internal mammary lymph node involvement
N3a	Metastasis in ipsilateral infraclavicular lymph node(s)
N3b	Metastasis in ipsilateral internal mammary lymph node(s) and axillary lymph node(s)
N3c	Metastasis in ipsilateral supraclavicular lymph node(s)

**Clinically detected* is defined as detected by imaging studies (excluding lymphoscintigraphy) or by clinical examination or grossly visible pathologically.

Pathologic (pN)[a]

pNX	Regional lymph nodes cannot be assessed
pN0	No regional lymph node metastasis identified histologically

NOTE: Isolated tumor cells (ITC) are defined as single tumor cells or small cell clusters not greater than 0.2 mm, usually detected only by immunohistochemical (IHC) or molecular methods but which may be verified on H&E stains. ITCs do not usually show evidence of malignant activity e.g., proliferation or stromal reaction.

SWOG Authorized Document

pN0 (i-)	No regional lymph node metastasis histologically, negative IHC
pN0 (i+)	Malignant cells in regional lymph node(s) no greater than 0.2mm (detected by H&E or IHC including ITC)
pN0(mol-)	No regional lymph node metastasis histologically, negative molecular findings (RT-PCR)[b]
pN0(mol+)	No regional lymph node metastasis histologically, positive molecular findings (RT-PCR)

[a] Classification is based on axillary lymph node dissection with or without sentinel lymph node dissection. Classification based solely on sentinel lymph node dissection without subsequent axillary lymph node dissection is designated (sn) for "sentinel node," e.g., pN0 (i+) (sn).

[b] RT-PCR: reverse transcriptase/polymerase chain reaction.

pN1	Metastasis in 1 to 3 axillary lymph nodes, and/or in internal mammary nodes with microscopic disease detected by sentinel lymph node dissection but not clinically detected**
pN1mi	Micrometastasis (greater than 0.2 mm, none greater than 2.0 mm)
pN1a	Metastasis in 1 to 3 axillary lymph nodes, at least one metastasis is greater than 2.0mm
pN1b	Metastasis in internal mammary nodes with microscopic disease detected by sentinel lymph node dissection but not clinically detected.**
pN1c	Metastasis in 1 to 3 axillary lymph nodes and in internal mammary lymph nodes with microscopic disease detected by sentinel lymph node dissection but not clinically detected.** (If associated with greater than 3 positive axillary lymph nodes, the internal mammary nodes are classified as pN3b to reflect increased tumor burden)
pN2	Metastasis in 4 to 9 axillary lymph nodes or in clinically detected* internal mammary lymph nodes in the *absence* of axillary lymph node metastasis
pN2a	Metastasis in 4 to 9 axillary lymph nodes (at least one tumor deposit greater than 2.0 mm)
pN2b	Metastasis in clinically detected* internal mammary lymph nodes in the *absence* of axillary lymph node metastasis
pN3	Metastasis in 10 or more axillary lymph nodes, or in infraclavicular lymph nodes, or in clinically detected* ipsilateral internal mammary lymph nodes in the *presence* of 1 or more positive axillary lymph nodes; or in more than 3 axillary lymph nodes with clinically negative microscopic metastasis in internal mammary lymph nodes; or in ipsilateral supraclavicular lymph nodes
pN3a	Metastasis in 10 or more axillary lymph nodes (at least one tumor deposit greater than 2.0 mm), or metastasis to the infraclavicular lymph nodes
pN3b	Metastasis in clinically detected* ipsilateral internal mammary lymph nodes in the *presence* of 1 or more positive axillary lymph nodes; or in more than 3 axillary lymph nodes and in internal mammary lymph nodes with microscopic disease detected by sentinel lymph node dissection but not clinically detected**
pN3c	Metastasis in ipsilateral supraclavicular lymph nodes

* *Clinically detected* is defined as detected by imaging studies (excluding lymphoscintigraphy) or by clinical examination.

** *Not clinically detected* is defined as not detected by imaging studies (excluding lympho-seintiography) or by clinical examination.

SWOG Authorized Document

Distant Metastasis (M)

M0 No distant metastasis

STAGE GROUPING

Stage IA	T1*	N0	M0
Stage IB	T0	N1mi	M0
	T1*	N1mi	M0
Stage IIA	T0	N1**	M0
	T1*	N1**	M0
	T2	N0	M0
Stage IIB	T2	N1	M0
	T3	N0	M0
Stage IIIA	T0	N2	M0
	T1*	N2	M0
	T2	N2	M0
	T3	N1	M0
	T3	N2	M0
Stage IIIB	T4	N0	M0
	T4	N1	M0
	T4	N2	M0
Stage IIIC	Any T	N3	M0

*T1 includes T1mic
** T0 and T1 tumors with nodal micrometastases only are excluded from Stage IIA and ar classified Stage IB.

NOTE: Stage designation may be changed if post-surgical imaging studies reveal the presence of distant metastases, provided the studies are carried out within 4 months of diagnosis in the absence of disease progression and provided that the patient has not received neoadjuvant therapy.

SWOG Authorized Document

5.0 ELIGIBILITY CRITERIA

Each of the criteria in the following section must be met in order for a patient to be considered eligible for registration. Use the spaces provided to confirm a patient's eligibility. For each criterion requiring test results and dates, please record this information on the Prestudy Form (Form #41314) and submit to the Data Operations Center in Seattle (see Section 14.0). Any potential eligibility issues should be addressed to the Data Operations Center in Seattle at 206/652-2267 prior to registration.

In calculating days of tests and measurements, the day a test or measurement is done is considered Day 0. Therefore, if a test is done on a Monday, the Monday four weeks later would be considered Day 28. This allows for efficient patient scheduling without exceeding the guidelines. **If Day 28 falls on a weekend or holiday, the limit may be extended to the next working day.**

SWOG Patient No. ______________________

Patient's Initials (L, F, M) _______________________

______ 5.1 Patients must be women with histologically confirmed primary invasive carcinoma of the breast (Stage I,II, or III) with no evidence of metastatic disease (M0) (see Section 4.0). Patients must have undergone modified radical mastectomy or breast sparing surgery. Patients must have recovered from all side-effects of the surgery.

______ 5.2 Patients must be postmenopausal, as defined by at least one of the following:

a. ≥ 12 months since the last menstrual period OR

b. prior bilateral oophorectomy OR

c. current use of a GnRH agonist OR

d. previous hysterectomy with one or both ovaries left in place (or previous hysterectomy in which documentation of bilateral oophorectomy is unavailable) AND FSH values consistent with the institutional normal values for the postmenopausal state. If patient is under the age of 55, FSH levels must be obtained within 28 days prior to registration.

______ 5.3 Patients must be positive for either estrogen receptor (ER) and/or progesterone receptor (PgR) as determined by institutional standard.

______ 5.4 Patients must currently be taking a third-generation aromatase inhibitor (AI) – anastrozole, letrozole, or exemestane for at least the previous 90 days prior to registration with plans to continue for at least an additional 1 year after registration. Patients may have switched AIs provided that they have been on a stable dose for at least 90 days. Concurrent trastuzumab (herceptin) is allowed.

______ 5.5 Patients must have completed the **S1200** Brief Pain Inventory-Short Form (BPI-SF) (Form #40963) within 14 days prior to registration. Patients must have a worst pain score of at least 5 on the Brief Pain Inventory (item #2) that has started or increased since starting AI therapy.

______ 5.6 Patients must have a Zubrod performance status of 0 to 1 (see Section 10.3).

______ 5.7 Patients must not have had prior acupuncture treatment within the past 12 months or for AI-induced joint symptoms at any time.

______ 5.8 Patients must not have a severe bleeding disorder.

SWOG Authorized Document

SWOG Patient No. ___________________________________

Patient's Initials (L, F, M) ______________________

______ 5.9 Patients must not have concurrent medical/arthritic disease that could confound or interfere with evaluation of pain or efficacy including: inflammatory arthritis (e.g., rheumatoid arthritis, systemic lupus, spondyloarthropathy, psoriatic arthritis, polymyalgia rheumatica), gout, episodes of acute monoarticular arthritis clinically consistent with pseudogout, Paget's disease affecting the study joint (knees/hands), a history of septic arthritis or avascular necrosis or intra-articular fracture of the study joint, Wilson's disease, hemochromatosis, alkaptonuria, or primary osteochondromatosis.

______ 5.10 Patients must not have a history of bone fracture or surgery of the afflicted knees and/or hands within 6 months prior to registration.

______ 5.11 Patients must not have a history of illness that, in the opinion of the investigator, might confound the results of the study or pose additional risk to the patient.

______ 5.12 Patients must not be on narcotics within 14 days of registration.

______ 5.13 Patients must not have received oral corticosteroids, intramuscular corticosteroids, or intra-articular steroids within 28 days prior to registration.

______ 5.14 Patients must not have received topical analgesics (e.g., capsaicin preparations) or any other analgesics (e.g., opiates, tramadol, with the exception of NSAIDs and acetaminophen) within 14 days prior to registration.

______ 5.15 Patients must not have received or implemented any other medical therapy, alternative therapy or physical therapy for the treatment of joint pain/stiffness within 28 days prior to registration. Therapeutic massage is allowed.

______ 5.16 Patients must be willing to submit blood and urine samples for serum hormones (estradiol, FSH, LH), inflammatory biomarkers (serum TNFα, IL-6, IL-12, CRP and urine CTX-II), urine AI metabolites, and DNA analysis *(CYP19A1),* and must be given the option to consent to use of remaining specimens for future translational medicine studies as outlined in Section 15.0. Baseline samples must be obtained prior to beginning intervention.

______ 5.17 Patients must be able to complete study questionnaires in English.

______ 5.18 No other prior malignancy is allowed except for adequately treated basal cell or squamous cell skin cancer, *in situ* cervical cancer, DCIS, adequately treated Stage I or II cancer from which the patient is currently in complete remission, or any other cancer for which the patient has been disease-free for > 5 years.

______ 5.19 All patients must be informed of the investigational nature of this study and must sign and give written informed consent in accordance with institutional and federal guidelines.

______ 5.20 At the time of patient registration, the treating institution's name and ID number must be provided to the Data Operations Center in Seattle in order to ensure that the current (within 365 days) <u>date of institutional review board approval</u> for this study has been entered into the database.

SWOG Authorized Document

6.0 STRATIFICATION FACTORS

Patients will be randomized using a 2:1:1 ratio to true acupuncture vs sham acupuncture vs waitlist control. Patient randomization will be dynamically balanced according to study site at time of registration.

7.0 PROCEDURES AND STUDY PLAN

For study-related questions, please contact Dr. Dawn Hershman at 212/305-1945 or Dr. Katherine Crew at 212/305-1732.

7.1 All patients, whether receiving acupuncture treatments (either true or sham) or assigned to the waitlist control group, will have the same clinic visit and telephone contact schedule, including patient-completed questionnaires and functional testing. See Appendix 19.1 for a description of true acupuncture, sham acupuncture, waitlist control.

7.2 Initial Visit

Before beginning the intervention, patients will have completed the following self-administered questionnaires:

- **S1200** Brief Pain Inventory-Short Form (BPI-SF) (Form #40963)
- **S1200** Western Ontario and McMaster Universities Osteoarthritis (WOMAC) Index (Version 3.1) (Form #33579)
- **S1200** Modified-Score for the Assessment and Quantification of Chronic Rheumatoid Affections of the Hands (M-SACRAH) (Form #48347)
- **S1200** PROMIS Pain Impact-Short Form (PROMIS PI-SF) (Form #28494)
- **S1200** FACT-ES Trial Outcome Index (Version 4) (Form #13659)
- **S1200** Aromatase Inhibitor Usage Form (Form #39879)

It should take the patient about 30 minutes to complete the assessments. **See Section 15.2 for instructions for administration of the questionnaires.** The nurse or CRA must complete the **S1200** Cover Sheet for Patient-Completed Questionnaires (Form #19445). Use of pain medications will be recorded on the **S1200** Supplemental Agents Reporting Form (Form #9413).

Functional testing (grip strength, 'Timed Get Up and Go') will be conducted and recorded on the **S1200** Functional testing (Form #37709). **See Section 7.5 for instructions for functional testing.**

Additionally, blood will be drawn for serum hormones (estradiol, FSH, LH) and inflammatory markers (TNFα, IL-6, IL-12, CRP) and for DNA analysis *(CYP19A1)* (see Section 15.1). Urine will be collected for urine CTX-II and urine AI metabolites.

7.3 Follow-Up Visits

a. Follow-up visits will occur at 6 and 12 weeks after registration.

Acupuncture adherence will be ascertained by the Columbia CRA by maintenance of the acupuncturist-completed web-based study calendar and documentation on the **S1200** Adherence to Acupuncture Form (Form #6044). Adverse events will be assessed and recorded on the **S1200** Adverse Event Summary Form (Form #5183). Use of pain medications will be recorded on the **S1200** Supplemental Agents Reporting Form (Form #9413). At 6 weeks and 12 weeks, to assess the adequacy of blinding, patients in the true and sham acupuncture groups will give an opinion about their treatment assignment. All of this data will be recorded on the **S1200** Assessment of Blinding (Form #20574).

SWOG Authorized Document

Patients will be instructed to complete the following self-administered questionnaires. It takes about 30 minutes to complete the assessments. **See Section 15.2 for instructions for administration of the questionnaires.**

- **S1200** Brief Pain Inventory-Short Form (BPI-SF) (Form #40963)
- **S1200** Western Ontario and McMaster Universities Osteoarthritis (WOMAC) Index (Version 3.1) (Form #33579)
- **S1200** Modified-Score for the Assessment and Quantification of Chronic Rheumatoid Affections of the Hands (M-SACRAH) (Form #48347)
- **S1200** PROMIS Pain Impact-Short Form (PROMIS PI-SF) (Form #28494)
- **S1200** FACT-ES Outcome Trial Outcome Index (Version 4) (Form #13659)
- **S1200** Aromatase Inhibitor Usage Form (Form #39879)

The nurse or CRA will also complete the **S1200** Cover Sheet for Patient-Completed Questionnaires (Form #19445).

Functional testing (grip strength, 'Timed Get Up and Go') will be conducted and recorded on the **S1200** Functional testing (Form #37709). **See Section 7.5 for instructions for functional testing.**

Additionally, blood will be drawn for serum hormones (estradiol, FSH, LH) and inflammatory markers (TNFα, IL-6, IL-12, CRP) (see Section 15.2). Urine will be collected for urine CTX-II.

b. Follow-up visits after the 12-week study intervention will occur at 24 and 52 weeks after registration.

Patients will be instructed to complete the same self-administered questionnaires as listed in Section 7.3a.

The nurse or CRA will also complete the **S1200** Cover Sheet for Patient-Completed Questionnaires (Form #19445).

Functional testing (grip strength, 'Timed Get Up and Go') will be conducted and recorded on the **S1200** Functional testing (Form #37709). **See Section 7.5 for instructions for functional testing.**

At 24 and 52 weeks, use of pain medications will be recorded on the **S1200** Supplemental Agents Reporting Form (Form #9413). To assess the adequacy of blinding, patients in the true and sham acupuncture groups will give an opinion about their treatment assignment and all three groups will receive a voucher for 10 free true acupuncture sessions to be administered by the study acupuncturist(s) and used off-study at her convenience. Information on additional acupuncture received off-study will be collected. All of this data will also be recorded on the **S1200** Assessment of Blinding (Form #20574).

Additionally, at 24 weeks, blood will be drawn for serum hormones (estradiol, FSH, LH) and inflammatory markers (TNFα, IL-6, IL-12, CRP) and urine will be collected for CTX-II. At 24 and 52 weeks, urine for AI metabolites will be collected (see Section 15.1).

SWOG Authorized Document

7.4 Telephone Contact

During telephone contact by the CRA at 2 and 4 weeks, acupuncture adherence will be ascertained and documented on the **S1200** Adherence to Acupuncture Form (Form #6044) for those patients assigned to receive acupuncture treatment. Adverse events will be assessed and recorded on the **S1200** Adverse Event Summary Form (Form #5183). Use of pain medications will be recorded on the **S1200** Supplemental Agents Reporting Form (Form #9413).

During telephone contact by the CRA at 16 and 20 weeks, the BPI-SF will be administered and documented on the **S1200** Brief Pain Inventory-Short Form (BPI-SF) (Form #40963). Use of pain medications will be recorded on the **S1200** Supplemental Agents Reporting Form (Form #9413). The nurse or CRA will also complete the **S1200** Cover Sheet for Patient-Completed Questionnaires (Form #19445).

7.5 Functional Testing

At baseline, Week 6, Week 12, Week 24, and Week 52, functional testing of the hands and legs will be conducted. Grip strength will be measured with a Detecto DHS 88 Digital Hand Grip Strength Dynamometer in kilograms (kg). (*141*) The dominant hand will be recorded and both hands will be tested. Patients will be asked to make 3 maximal voluntary contractions (MVC), with 1 minute between each. Verbal encouragement will be given and the read out of each contraction, in kg, will allow visual feedback. If these readings are not within < 10% of each other, further contractions will be undertaken, with 1 minute between each, until they are repeatable. Once repeatable, only the maximum contraction will then be taken to represent the MVC strength.

The "Timed Get Up and Go" (TGUG) test is a physical function assessment tool that measures balance and gait. (*142*) The test uses a straight-backed chair with arms and a seat height of 42 cm. Lower extremity strength is measured when the patient is asked to stand up from the chair without using her arms, walk 3 meters (10 feet), turn around, walk back to the chair, and be seated. The subject is timed with a stopwatch from the command 'go' until she returns to a seated position in the chair. Many recently developed measures of physical activity use timing of tasks to improve objectivity of the scoring. Timing of task performance has been found to provide greater discrimination of differences in ability, especially in elderly subjects with higher functional abilities. *(146)*

7.6 Acupuncture Treatment

a. Acupuncture Schedule

Patients will be randomized (2:1:1) to true acupuncture vs. sham acupuncture vs. waitlist control. Patients will be blinded to true acupuncture vs. sham acupuncture. Patients will not be blinded to the waitlist control. The study intervention is to begin within 5 working days of registration.

- True acupuncture administered twice weekly for 6 weeks followed by weekly treatments for 6 weeks (12-18 sessions total over 12 weeks).
- Sham acupuncture administered twice weekly for 6 weeks followed by weekly treatments for 6 weeks (12-18 sessions total over 12 weeks).
- Duration of intervention – Study intervention will be continued for 12 weeks unless unacceptable toxicity occurs, the subject has concurrent illness that necessitates withdrawal or subject decides to withdraw from participation for any reason. If the subject's medical condition is influenced by the knowledge of which study intervention she was assigned, there will be unblinding upon the Investigator's request.

SWOG Authorized Document

- A licensed Study Acupuncturist(s) designated at each site will administer the true and sham acupuncture treatments.
- After the 24-week visit, all patients will receive a voucher for 10 free true acupuncture sessions administered by the Study Acupuncturist(s) and to be used at the patient's convenience and documented in the web-based calendar.

During the first 6 weeks of acupuncture, the twice weekly acupuncture sessions should be at least 24 hours apart and if a session is missed, it may be made up the following week for up to 3 sessions per week. The schedule will be flexible to allow for 8-12 sessions during the first 6 weeks, which may be more reflective of what may happen in the "real world" setting. During the second 6 weeks of weekly maintenance acupuncture, there will be no make-up of missed sessions, so patients may have only 1 session per week during the second 6 weeks of the study intervention (Week 7 to 12). Breaks of up to 30 consecutive days are allowed.

b. Documentation of Acupuncture Visits

The Study Acupuncturist(s) must maintain a careful record of the acupuncture visits and the true or sham point prescription administered at each visit, which will be captured on the proper electronic case report forms (e-CRFs). To maintain blinding, the data on the true or sham point prescription administered at each visit will be entered into a separate locked database, which will only be accessible to the Study Acupuncturist(s) and an unblinded Site Administrator at CUMC who will not have contact with patients.

7.7 Criteria for Removal from Protocol Intervention*:

a. Evidence of new cancer or cancer recurrence at any time.

b. Unacceptable toxicity (see Section 8.1).

c. Delay of 30 consecutive days due to any reason after study registration.

d. The patient may withdraw from the study at any time for any reason.

e. Completion of 12 weeks of study intervention (acupuncture). (NOTE: Patients randomized to Waitlist Control are considered off protocol intervention after 12 weeks.)

* Under intent-to-treat, all follow-up assessments will continue, if possible, whether the patient completes intervention or has early removal from intervention.

7.8 Additional Instructions for Patient-Completed Questionnaires

If a patient goes off protocol intervention before the schedule for patient-completed questionnaires has been completed, please administer all patient-completed questionnaires at the scheduled assessment times, even if the patient has begun another treatment. The 6, 12, 24, and 52 week assessment times as well should be defined from registration.

The **S1200** Cover Sheet for Patient-Completed Questionnaires (Form #19445) is required with each set of patient-completed questionnaires indicating whether or not the assessment occurred, if assistance was required, and the location of the assessment. If one or all of the questionnaires were not administered, an overall reason must be indicated on the **S1200** Cover Sheet for Patient-Completed Questionnaires (Form #19445). Please see Section 15.2 for more detailed instructions for the questionnaires.

SWOG Authorized Document

7.9 All reasons for study discontinuation must be documented on the **S1200** Off Protocol Notice (Form #9707).

7.10 All patients will be followed for one year from registration. No further follow-up will be required once the patient completes the 52-week visit.

8.0 SAFETY MONITORING

8.1 This study will utilize the CTCAE (NCI Common Terminology Criteria for Adverse Events) Version 4.0 for toxicity and Reportable Adverse Event reporting. A copy of the CTCAE Version 4.0 can be downloaded from the CTEP home page (http://ctep.cancer.gov). All appropriate treatment areas should have access to a copy of the CTCAE Version 4.0.

8.2 Acupuncture Schedule Adjustments

The main toxic effects of acupuncture include a slightly increased risk of minor bruising and bleeding and rarely infection. Needle shock, a vaso-vagal response to needle insertion, occurs about 5% of the time usually during the first or second treatment. *(147)* Patients may become pre-syncopal, diaphoretic, nauseated and occasionally lose consciousness. These symptoms are easily reversible by removing the acupuncture needles and laying the patient supine. Toxicity will be assessed during telephone contact at 2 and 4 weeks and during study visits at 6 and 12 weeks.

8.3 Reporting adverse events and referrals for patient care

During the study, all study patients will be under the care of either a medical oncologist or a surgical oncologist. If any abnormalities or problems, including possible adverse events (AE) or reportable adverse events, are detected during screening, subsequent measurements, or study activities, the patient will be referred back to their medical provider for appropriate care. The site CRAs and site investigators will be responsible for recording and reporting any AEs and reportable adverse events. The possible routes of referral are as follows:

a. Abnormality/problem detected during screening and subsequent clinic visits: The CRA will notify and discuss with the responsible site investigator. The patient will be referred to their medical provider by the CRA and the responsible site investigator as appropriate. The CRA and responsible site investigator will record and report AEs and reportable adverse events as appropriate.

b. Abnormality/problem detected during telephone contact: The CRA will refer the patient to their medical provider and the responsible site investigator as appropriate. The CRA and responsible site investigator will record and report AEs and reportable adverse events as appropriate.

c. Abnormality/problem detected during acupuncture session: The Study Acupuncturist will notify and discuss with the responsible site investigator. The CRA will be notified and the patient will be referred to their medical provider by the CRA and the responsible site investigator as appropriate. The CRA and responsible site investigator will record and report AEs and reportable adverse events as appropriate.

8.4 For study related questions, please contact Dr. Dawn Hershman at 212/305-1495 or Dr. Katherine Crew at 212/305-1732.

8.5 Toxicities (including suspected reactions) that meet the expedited reporting criteria as outlined in Section 16.0 of the protocol must be reported to the Operations Office, Study Coordinator and NCI via AdEERS, and to the IRB per local IRB requirements.

SWOG Authorized Document

9.0 STUDY CALENDAR **S1200**, "Randomized Blinded Sham- and Waitlist-Controlled Trial of Acupuncture for Joint Symptoms Related to Aromatase Inhibitors in Women With Early Stage Breast Cancer"

								α						α			π	
**REQUIRED STUDIES**	PRE	BASELINE	Wk	Wk	Wk	Wk	Wk	Wk	Wk	Wk	Wk	Wk	Wk	Wk	Wk	Wk	Wk	Wk
	STUDY		1	2	3	4#	5	6 #	7	8	9	10	11	12#	16^	20^	24^	52^
**PHYSICAL**																		
History (demographics, comorbidities, medications)	X																	
Physical Exam (height, weight, performance status)	X							X						X			X	X
Telephone Contact				X†		X†									X~	X~		
Functional Testing		X						X						X			X	X
Toxicity Notation				X		X		X						X				
**LABORATORY**																		
FSH (if necessary)	X																	
ER/PgR	X																	
**QUESTIONNAIRES**																		
**S1200** Cover Sheet for Patient-Completed Questionnaires	X	X						X						X	X	X	X	X
**S1200** BPI-SF	X							X						X	X	X	X	X
**S1200** WOMAC		X						X						X			X	X
**S1200** M-SACRAH		X						X						X			X	X
**S1200** PROMIS PI-SF		X						X						X			X	X
**S1200** FACT-ES		X						X						X			X	X
**S1200** AI Usage Form		X						X						X			X	X
**PROCEDURES**																		
Blood for Serum Hormones √ β		X						X						X			X	
Blood for Inflammatory Markers √ β		X						X						X			X	
Blood for DNA √ β		X																
Urine for CTX-II β		X						X						X			X	
Urine for AI metabolites β		X															X	X
**TREATMENT**																		
True or Sham Acupuncture*			X	X	X	X	X	X	X	X	X	X	X	X				
Waitlist Control %																		

NOTE: Forms are found in Section 18.0. Forms submission guidelines are found in Section 14.0.

√ Blood will be collected to measure serum hormones (estadiol, FSH, LH) and inflammatory biomarkers (TNF, IL-6, IL-12, CRP) at baseline, weeks 6, 12, and 24. Plasma and buffy coat will be collected at baseline only for DNA extraction and genotyping (CYP19A1).

β See Section 15.1 and order kit from SWOG Specimen Repository.

α If the study visit occurs on the same day as an acupuncture visit, then the evaluations should be conducted prior to acupuncture treatment. Study visit to include assessment of adherence to acupuncture use of pain medication, adverse events, and adequacy of blinding.

+/- 3 days to allow flexibility in scheduling of assessments.

^ +/- 7 days to allow flexibility in scheduling of assessments.

π After the Week 24 visit, all patients will receive a voucher for 10 free true acupuncture sessions to be administered by the study acupuncturist(s) and used off-study at her convenience.

* True/Sham acupuncture will be administered twice weekly for 6 weeks (12 sessions) followed by 6 weekly treatments (6 sessions) of maintenance (18 sessions total over 12 weeks).

% Patients on the Waitlist Control arm will not receive any acupuncture (true or sham) until given the vouchers at Week 24. All forms must be submitted as required in Section 14.0.

† Telephone contact at Weeks 2 and 4 will include assessment at adherence to acupuncture, use of pain medications, and adverse events.

~ Telephone contact at Weeks 16 and 20 will include phone administration of the **S1200** Brief Pain Inventory - Short Form (Form #40963) and assessment of use of pain medications.

SWOG Authorized Document

10.0 CRITERIA FOR EVALUATION AND ENDPOINT DEFINITIONS

10.1 Primary Endpoint:

Reduction in worst joint pain at 6 weeks between the true acupuncture compared to sham acupuncture and waitlist control groups: A difference of two points in the modified Brief Pain Inventory worst pain score (item #2) has been identified as a clinically meaningful difference. *(148)* This item has a scale of 0 to 10 with 0 indicating "No pain" and 10 indicating "Pain as bad as you can imagine".

10.2 Secondary Endpoints:

To investigate the effects of true acupuncture compared to sham acupuncture and waitlist control on the following outcome measures compared to baseline:

a. Brief Pain Inventory-Short Form (BPI-SF) worst pain, worst stiffness (items 15 & 16), pain severity, and pain-related interference scores at 6, 12, 16, 20, 24, and 52 weeks.

b. Western Ontario and McMaster Universities Osteoarthritis (WOMAC) index (pain, stiffness, and function) for the hips and knees at 6, 12, 24, and 52 weeks.

c. Modified-Score for the Assessment and Quantification of Chronic Rheumatoid Affections of the Hands (M-SACRAH) (pain, stiffness, and function) at 6, 12, 24, and 52 weeks.

d. PROMIS Pain Impact-Short Form (PROMIS PI-SF) at 6, 12, 24, and 52 weeks.

e. Quality of life as assessed by the FACT-ES Trial Outcome Index (Version 4) at 6, 12, 24, and 52 weeks.

f. Functional testing of the hands (grip strength) and legs ('Timed Get Up and Go') at 6, 12, 24, and 52 weeks.

g. Analgesic and opioid use at 2, 4, 6, 12, 16, 20, 24, and 52 weeks

h. AI adherence at 12, 24, and 52 weeks.

i. Urine AI metabolites at 24 and 52 weeks.

j. Serum hormone biomarkers (estradiol, FSH, LH) and inflammatory biomarkers (serum TNFα, IL-6, IL-12, CRP; urine CTX-II) at 6, 12, and 24 weeks.

k. Polymorphisms in *CYP19A1.*

l. Safety and tolerability of acupuncture.

This study will also describe adverse events, assess the association between *CYP19A1* genotype and severity of AI-related joint symptoms, and assess the adequacy of blinding and use of additional off-study acupuncture treatments.

SWOG Authorized Document

10.3 Performance Status: Patients will be graded according to the Zubrod performance status scale.

POINT	DESCRIPTION
0	Fully active, able to carry on all pre-disease performance without restriction.
1	Restricted in physically strenuous activity but ambulatory and able to carry out work of a light or sedentary nature, *e.g.,* light housework, office work.
2	Ambulatory and capable of self-care but unable to carry out any work activities; up and about more than 50% of waking hours.
3	Capable of limited self-care, confined to bed or chair more than 50% of waking hours.
4	Completely disabled; cannot carry on any self-care; totally confined to bed or chair.

11.0 STATISTICAL CONSIDERATIONS

11.1 The primary hypothesis of the study is that true acupuncture will decrease joint pain associated with the use of AIs in breast cancer patients compared to sham acupuncture or waitlist control at 6 weeks. Joint pain will be assessed using “worst pain” according to the BPI. Enrollees must currently be taking AIs and must exhibit joint pain with a minimum BPI worst pain score of 5; scores of 5 to 10 are considered to reflect moderate to severe pain. *(149)* Patients will be stratified at randomization by study site to account for potential site-specific variation in the administration of acupuncture. The primary endpoint of 6 weeks was chosen in order to confirm initial results of a 6-week acupuncture intervention given twice weekly in a larger multicenter study. *(80)*

The study team stipulates an alpha=.025 two-sided test (to account for two comparisons; i.e., true acupuncture vs. sham acupuncture and true acupuncture vs. waitlist control), with an estimated 5% non-adherence (reducing the nominal effect size) and 10% dropout rate (increasing the total required sample size) at the primary endpoint evaluation time of 6 weeks after randomization. In addition, the design will incorporate a 10% contamination rate (which also reduces the nominal effect size) based on the assumption that a portion of the patients will have joint pain with a different etiology (that is, not biologically associated with AI treatment and therefore not amenable to the specified intervention). The subjects will be randomized 2:1:1 to: 1) true acupuncture, 2) sham acupuncture, or 3) waitlist control. Power for the design will be a function of the difference detected and the standard deviation (SD) at 6 weeks after randomization. Data on the use of the BPI to assess joint pain from AIs is limited. A difference of 2 points in the BPI worst pain score has been identified as a clinically meaningful difference. *(148)* In the pilot single institution randomized sham acupuncture-controlled trial for AI-induced joint pain, the standard deviation for BPI “worst pain” at 6 weeks on the control arm was 2.31. *(88)* For design purposes for this multicenter acupuncture study, a more conservative estimate of standard deviation of 3.0 points will be assumed. Given the number controls in the prior study was small (n=18), assumption of a standard deviation (SD) of 3.0 points for this study is likely conservative.

SWOG Authorized Document

For a 2-point difference and a 3.0 point SD at 6 weeks, with other parameters as specified above, 208 total eligible patients (104 true acupuncture, 52 sham acupuncture, and 52 waitlist control) would be required for 82% power for the two comparisons, based on two separate t-tests. [Note that in power calculations, the combination of the 5% non-adherence rate and the 10% contamination rate, stipulated above, reduces the nominal effect size of a 2 point difference to 1.7 points; while the 10% dropout rate inflates the estimated sample size by a factor of 1/.9 or about 11%.] This approach allows two fully-powered independent comparisons between true acupuncture and the two alternatives. In addition, unlike an ANOVA approach, it does not assume equal variances across the three arms, which may be more appropriate in this setting given the inclusion of a waitlist control. Power will be higher with lower observed standard deviation.

A multiple linear regression analysis of the primary endpoint will include the baseline score as well as the pre-specified stratification factor. To allow for an ineligibility of 8% (based on a recent study, **S0715**), a total of 228 patients will be enrolled to achieve 208 eligible patients.

The potential for different dropout by arm, especially with respect to the waitlist control, will be mitigated by monthly conferences among study investigators to encourage proper assessment and submission of forms at every required timepoint for all patients. Dropout patterns will be monitored on an ongoing basis.

11.2 Secondary endpoints:

a. Secondary hypotheses (Section 10.2) will include assessment of intervention differences at each timepoint individually (weeks 6, 12, 16, 20, 24, and 52), as well as longitudinal modeling of the outcome measures over time. For longitudinal modeling, linear mixed models will be used; if there is evidence of non-random dropout, pattern-mixture models will be utilized as sensitivity analysis. Covariates for longitudinal modeling will include intervention assignment, assessment time, their interaction, baseline score, and other potential confounding variables (*i.e.* age).

b. Exploratory analyses will also be done to evaluate the relationship between aromatase gene polymorphisms *(CYP19A1)* and inflammatory biomarkers with severity of joint symptoms and response to acupuncture treatment.

c. A further exploratory analysis at both 6 and 12 weeks according to adherence to intervention assignment will be conducted, with receipt of ≥ 80% of planned treatment sessions defined as "adherent." (In particular, patients will be divided into the adherent true (≥ 80%), non-adherent true (< 80%), adherent sham (≥ 80%), and non-adherent sham (< 80%) acupuncture groups.) The interaction between treatment adherence category and treatment category will be tested. Adherence to acupuncture sessions as a continuous measure will also be evaluated.

d. The success of blinding will be evaluated by using chi-square tests to compare the percentage of patients in each group who believed that they had received true acupuncture at Weeks 6, 12, 24, and 52.

11.3 Sample Size/Accrual Rate

Based upon a previous cross-sectional survey at Columbia University, 253 consecutive breast cancer patients on adjuvant AI therapy were screened over a 4-month period; 47% reported joint pain which started or worsened after initiation of AI therapy and among those with AI-related arthralgias, about two-thirds reported a worst pain score ≥ 5 points on a 0-10 scale. Therefore, it is anticipated that up to 20 women screened in the clinic

SWOG Authorized Document

each month may be eligible for this trial. In addition, the Research Recruitment and Minority Outreach Core at Columbia University will be utilized to target women with breast cancer in the community. Based on this strategy, investigators were able to successfully recruit to other intervention studies for AI-related arthralgias, including acupuncture and supplements such as glucosamine/chondroitin and omega-3 fatty acids. Investigators will also advertise through breast cancer support groups and reach out to colleagues in the community who see breast cancer patients

Accrual is expected to be rapid given the common use of AIs for adjuvant therapy in postmenopausal women with breast cancer. Allowing for 6 months ramp-up and IRB approval time, accrual of 1.5 patients per month for each site would allow completion of the study accrual in approximately 2.5 years. Accrual will be assessed every 6 months after study activation. If monthly average accrual is < 50% 1 year after study activation revision will be considered. For an individual site, if there is no accrual in a 6 month period, the study team will evaluate barriers to accrual. If these changes do not improve accrual, the site will be closed.

11.4 A Data and Safety Monitoring Committee will oversee the conduct of the study. The Committee consists of four members from outside of SWOG, 3 SWOG members, 3 non-voting representatives from the National Cancer Institute (NCI), and the Group Statistician (non-voting). The members of this Committee will receive confidential reports every 6 months from the SWOG Statistical Center, and will meet at the Group's bi-annual meetings as necessary. The Committee will be responsible for decisions regarding possible termination and/or early reporting of the study.

12.0 DISCIPLINE REVIEW

There will be no formal discipline review for this study.

13.0 REGISTRATION GUIDELINES

13.1 Patients must be registered prior to initiation of study intervention (no more than 5 working days prior to planned start of study intervention).

13.2 The individual registering the patient must have completed the appropriate SWOG Registration Worksheet. The completed form must be referred to during the registration but should not be submitted as part of the patient data.

OPEN will also ask additional questions that are not present on the SWOG Registration Worksheet. The individual registering the patient must be prepared to provide answers to the following questions:

a. Institution CTEP ID

b. Protocol Number

c. Registration Step

d. Treating Investigator

e. Credit Investigator

f. Patient Initials

g. Patient’s Date of Birth

SWOG Authorized Document

h. Patient SSN (SSN is desired, but optional. Do not enter invalid numbers.)

i. Country of Residence

j. ZIP Code

k. Gender (select one):
- Female Gender
- Male Gender

l. Ethnicity (select one):
- Hispanic or Latino
- Not Hispanic or Latino
- Unknown

m. Method of Payment (select one):
- Private Insurance
- Medicare
- Medicare and Private Insurance
- Medicaid
- Medicaid and Medicare
- Military or Veterans Sponsored NOS
- Military Sponsored (Including Champus & Tricare)
- Veterans Sponsored
- Self Pay (No Insurance)
- No Means of Payment (No Insurance)
- Other
- Unknown

n. Race (select all that apply):
- American Indian or Alaska Native
- Asian
- Black or African American
- Native Hawaiian or other Pacific Islander
- White
- Unknown

13.3 Registration procedures

a. All site staff will use OPEN to enroll patients to this study. OPEN is a web-based application and can be accessed at https://open.ctsu.org, or from the OPEN tab on the CTSU members' side of the website at https://www.ctsu.org, or from the OPEN Patient Registration link on the SWOG CRA Workbench.

b. Prior to accessing OPEN site staff should verify the following:

- All eligibility criteria have been met within the protocol stated timeframes. Site staff should refer to Section 5.0 to verify eligibility.

- All patients have signed an appropriate consent form and HIPAA authorization form (if applicable).

c. Access requirements for OPEN:

- Site staff will need to be registered with CTEP and have a valid and active CTEP-IAM account. This is the same account (user ID and password) used for the CTSU members' web site.

SWOG Authorized Document

- To perform registrations on SWOG protocols you must have an equivalent 'Registrar' role on the SWOG roster. Role assignments are handled through SWOG.

Note: The OPEN system will provide the site with a printable confirmation of registration and treatment information. Please print this confirmation for your records.

d. Further instructional information is provided on the OPEN tab on the CTSU members' side of the website at https://www.ctsu.org or at https://open.ctsu.org. For any additional questions contact the CTSU Help Desk at 1-888-823-5923 or ctsucontact@westat.com.

13.4 Exceptions to SWOG registration policies will not be permitted.

a. Patients must meet all eligibility requirements.

b. Institutions must be identified as approved for registration.

c. Registrations may not be cancelled.

d. Late registrations (after initiation of treatment) will not be accepted.

14.0 DATA SUBMISSION SCHEDULE

14.1 Data must be submitted according to the protocol requirements for **ALL** patients registered, whether or not assigned treatment is administered, including patients deemed to be ineligible. Patients for whom documentation is inadequate to determine eligibility will generally be deemed ineligible.

14.2 Master forms are included in Section 18.0 and (with the exception of the sample consent form and the Registration Worksheet) must be submitted to the Data Operations Center in Seattle. Data from approved SWOG institutions must be submitted on-line via the Web; see Section 14.3a for details. Exceptions to online data submission are patient-completed (*e.g.,* Quality of Life) forms and source documents (*e.g.,* pathology/operative/lab reports).

14.3 Data Submission Procedures

a. SWOG institutions must submit data electronically via the Web by using the SWOG CRA Workbench. To access the CRA Workbench, go to the SWOG Web site (http://swog.org) and logon to the Members Area. After you have logged on, click on the *CRA Workbench* link to access the home page for CRA Workbench website. Next, click on the *Data Submission* link and follow the instructions. For new users, the link to a "Starter Kit" of help files may be found by clicking on the **Starter Kit** link at the Members' logon page.

To submit data via the web the following must be done (in order):

1. You are entered into the SWOG Roster and issued a SWOG Roster ID Number,
2. You are associated as an investigator or CRA/RN at the institution where the patient is being treated or followed, and
3. Your Web User Administrator has added you as a web user and has given you the appropriate system permissions to submit data for that institution.

SWOG Authorized Document

For assistance with points 1 and 2 call the Operations Office at 210/614-8808. For point 3, contact your local Web User Administrator (refer to the "Who is my Web User Administrator?" function on the swog.org Members logon page). For other difficulties with the CRA Workbench, please email *technicalquestion@crab.org*.

b. If you need to submit data that are not available for online data submission, the only alternative is via facsimile. Should the need for this occur, institutions may submit data via facsimile to 800/892-4007 or 206/342-1680 locally. Please do not use cover sheet for faxed data. Please make sure that each page of all faxed data include the SWOG patient number, study ID and patient initials.

14.4 AFTER REGISTRATION BUT PRIOR TO BEGINNING STUDY INTERVENTION (WITHIN 24 HOURS AFTER OBTAINING SPECIMENS):

Submit baseline blood and samples as described in Section 15.0. The SWOG Specimen Tracking System must be used for sample submission.

14.5 WITHIN 7 DAYS AFTER REGISTRATION:

Submit the following:

a. **S1200** Prestudy Form (Form #41314).

b. **S1200** Functional Testing (Form #37709)

c. **S1200** Supplemental Agents Reporting Form (Form #9413)

d. Institutional surgical pathology report to confirm staging.

e. **S1200** Cover Sheet for Patient-Completed Questionnaires (Form #19445)

f. **S1200** Brief Pain Inventory-Short Form (BPI-SF) (Form #40963)

g. **S1200** Western Ontario and McMaster Universities Osteoarthritis (WOMAC) Index (Version 3.1) (Form #33579)

h. **S1200** Modified-Score for the Assessment and Quantification of Chronic Rheumatoid Affections of the Hands (M-SACRAH) (Form #48347)

i. **S1200** PROMIS Pain Impact-Short Form (PROMIS PI-SF) (Form #28494)

j. **S1200** FACT-ES Trial Outcome Index (Version 4) (Form #13659)

k. **S1200** Aromatase Inhibitor Usage Form (Form #39879)

14.6 WITHIN 14 DAYS OF THE WEEK 2 AND 4 STUDY ASSESSMENTS:

Submit the following:

a. **S1200** Adherence to Acupuncture Form (Form #6044)

b. **S1200** Adverse Event Summary Form (Form #5183)

c. **S1200** Supplemental Agents Reporting Form (Form #9413)

SWOG Authorized Document

14.7 WITHIN 14 DAYS OF THE WEEK 6, 12, 24 AND 52 STUDY ASSESSMENTS:

Submit the following:

a. **S1200** Cover Sheet for Patient-Completed Questionnaires (Form #19445)

b. **S1200** Brief Pain Inventory-Short Form (BPI-SF) (Form #40963)

c. **S1200** Western Ontario and McMaster Universities Osteoarthritis (WOMAC) Index (Version 3.1) (Form #33579)

d. **S1200** Modified-Score for the Assessment and Quantification of Chronic Rheumatoid Affections of the Hands (M-SACRAH) (Form #48347)

e. **S1200** PROMIS Pain Impact-Short Form (PROMIS PI-SF) (Form #28494)

f. **S1200** FACT-ES Trial Outcome Index (Version 4) (Form #13659)

g. **S1200** Aromatase Inhibitor Usage Form (Form #39879)

h. **S1200** Functional Testing (Form #37709)

i. **S1200** Adherence to Acupuncture Form (Form #6044) [WEEK 6 AND 12]

j. **S1200** Adverse Event Summary Form (Form #5183)

k. **S1200** Supplemental Agents Reporting Form (Form #9413)

l. **S1200** Assessment of Blinding (Form #20574)

14.8 WITHIN 24 HOURS OF OBTAINING SPECIMENS AT THE WEEK 6, 12, 24 AND 52 STUDY ASSESSMENTS:

Submit blood and urine samples as described in Section 15.0.

14.9 WITHIN 14 DAYS OF THE WEEK 16 AND 20 STUDY ASSESSMENTS:

Submit the following:

a. **S1200** Brief Pain Inventory-Short Form (BPI-SF) (Form #40963)

b. **S1200** Supplemental Agents Reporting Form (Form #9413)

c. **S1200** Cover Sheet for Patient-Completed Questionnaires (Form #19445)

14.10 WITHIN 14 DAYS OF REMOVAL FROM PROTOCOL TREATMENT:

Submit a copy of the Off Protocol Notice (Form #9707) along with the final copy of the **S1200** Adverse Event Summary Form (Form #5183).

14.11 WITHIN 14 DAYS OF A DIAGNOSIS OF NEW OR RECURRENT CANCER OCCURRING UP TO 52 WEEKS AFTER REGISTRATION:

Submit copies of:

a. Pathology report documenting cancer

b. Follow-Up Form (Form #64587)

SWOG Authorized Document

14.12 WITHIN 4 WEEKS OF KNOWLEDGE OF DEATH OCCURRING UP TO 52 WEEKS AFTER REGISTRATION:

Submit the Notice of Death (Form #49467) documenting death information.

15.0 SPECIAL INSTRUCTIONS

15.1 Collection and Submission of Blood and Urine Specimens

Patients are required to submit blood samples for serum hormones (estradiol, FSH, LH), serum inflammatory markers (TNFα, IL-6, IL-12, CRP), blood for DNA analysis of *CYP19A1*, urine for CTX-II, and urine for AI metabolites as listed below.

	Baseline	Week 6	Week 12	Week 24	Week 52
Blood for Serum Hormones	X	X	X	X	
Blood for Inflammatory Markers	X	X	X	X	
Blood for DNA	X				
Urine for CTX-II	X	X	X	X	
Urine for AI metabolites	X			X	X

If the patient agrees, left over blood and urine will be banked for future translational medicine studies. **Institutions are required to seek additional patient consent to bank blood and urine for future translational medicine studies.**

a. Specimen collection and submission instructions can be accessed on the SWOG Specimen Submission webpage (https://swog.org/Members/ClinicalTrials/Specimens/STSpecimens.asp), or via the link on the **S1200** protocol abstract page on the SWOG website (www.swog.org). Additional collection instructions are located in Section 15.1c.

b. Specimen collection kits may be ordered by the SWOG Specimen Repository Management Application at http://ricapps.nationwidechildrens.org/BPCKitManagement/.

c. Additional Collection Instructions

1. Blood for serum hormones (estradiol, FSH, LDH) and inflammatory markers (TNFα, IL-6, IL-12, CRP) will be collected in two 10 mL red top tubes. Store samples at 4ºC for up to 24 hours prior to shipping.

2. Blood for DNA (*CYP19A1*) will be collected in a 10 mL purple top EDTA tube. Store sample at 4ºC for up to 24 hours prior to shipping.

3. Untreated urine will be collected in a 20 mL tube and stored at 4ºC for up to 24 hours prior to shipping.

SWOG Authorized Document

15.2 Patient Questionnaires: Instructions for Administration

a. Time frame for questionnaires

It is important to note that the time frame for providing ratings differs depending on the scale the patient is completing. The **S1200** Brief Pain Inventory Short Form (BPI-SF) (Form #40963), **S1200** Western Ontario and McMaster Universities Osteoarthritis (WOMAC) Index (Form #33579), **S1200** Modified-Score for the Assessment and Quantification of Chronic Rheumatoid Affections of the Hands (M-SACRAH) (Form #48347), **S1200** PROMIS Pain Impact-Short Form (PROMIS PI-SF) (Form #28494), and **S1200** FACT-ES Trial Outcome Index (Version 4) (Form #13659) should be rated with respect to the past 7 days. (See Section 14.0 for additional questionnaire submission requirements.)

If treatment is delayed due to toxicity, the assessment schedule for the Week 2, 4, 6, 12, 24 and 52 assessments should be defined from the date of registration.

b. Administration of Questionnaires

1. The first time the patient completes the questionnaires: Please read to the patient the instructions attached to each patient questionnaire. Explain the specific administration times for this protocol. Patients should be directed to report all symptoms and limitations whether or not they are related to the cancer or its treatment.

2. It is permissible to assist patients with completing the questionnaires being careful not to influence the patient's response. Note on the cover sheet what assistance was required and indicate reason (*e.g.,* elderly, too sick, etc.). Discourage family members from: 1) being present while the patient completes the questionnaire and/or 2) influencing patient responses to the questions.

3. It is very important to review the questionnaires after the patient has completed them to be sure all of the questions have been answered and that only one answer is marked. a) If the patient has marked more than one answer per question, ask the patient which answer reflects how she is feeling. b) If the patient has skipped a question, tell the patient that a question was not answered and ask if she would like to answer the question. Always give the patient the option to refuse. Indicate on the form by the question that the patient did not want to answer this question.

4. If a patient refuses or cannot complete the questionnaire for some reason, then this must be documented on the cover sheet and faxed to the Data Operations Center in Seattle (see Section 14.3b). This form will also be available for online submission.

5. If a patient misses an appointment or is too sick to complete the questionnaires on the scheduled date, the questionnaire can be mailed to the patient or sent home with her. A telephone interview must be scheduled and completed within one week of the originally scheduled time. Patient responses to questionnaire items are to be obtained during the telephone interview while the patient is looking at her copy of the questionnaire. The date of the telephone interview is to be noted on the cover sheet. In this study, the patient will also complete scheduled telephone assessments at Weeks 16 and 20 only. As noted above, ask the patient to have a copy of the BPI in front of her and then read each item and its possible response options. Do not discuss the patient's pain or help her perception of the pain for the study questions. Reinforce the idea that only the patient perception of pain is relevant for the study outcomes.

SWOG Authorized Document

c. Additional quality control procedures:

1. When a patient is registered on **S1200**, a calendar should be made with dates of upcoming patient-completed questionnaires noted. A copy of this calendar can be given to the patient with the notation that the questionnaires should be completed before receiving treatment. You may wish to photocopy the Study Calendar, Section 9.0, and include the patient's name and specific dates. A copy of this should be kept in the patient file.

2. If a patient goes off study intervention prior to the protocol-defined end of intervention at 12 weeks, administer the patient-completed questionnaires according to the protocol-defined assessment schedule (timed from registration date).

3. If a patient refuses or cannot complete the patient questionnaires at one time point, she should be asked to do so at the next scheduled administration time.

4. Anyone involved in the collection of quality of life data in SWOG trials should review the training program available on the SWOG website (www.swog.org). Please log on as a member and go to the **CRA Workbench**. Inside the Workbench, click on **Tools of the Trade** and select the training program: **Patient-Reported Outcome Questionnaires Training Program**. This program is a narrated set of slides designed to standardize the way quality of life data is collected from patients; it takes the place of the Quality of Life Training Video. Questions regarding the quality of life assessments can be addressed to Dr. Carol Moinpour at the Fred Hutchinson Cancer Research Center (206/677-4604).

d. **S1200** Cover Sheet for Patient-Completed Questionnaires

For each time point, the nurse or CRA completes the **S1200** Cover Sheet for Patient-Completed Questionnaires (Form #19445). The Cover Sheet is submitted with the set of patient-completed forms at each scheduled assessment. The Cover Sheet is very important for tracking how and when the patient forms were completed. When a patient-completed form is not administered at a scheduled time point, it is important to know why the assessment did not occur; the form includes potential reasons for a patient not completing a form. See Section 14.0 for data submission guidelines.

15.3 Acupuncture Quality Control: Instructions to Acupuncturist.

An acupuncturist from each site will be required to attend an initiation training session at the SWOG group meeting, watch the online training module, read and follow the training manual and train the other acupuncturists at the site. The other acupuncturists will be required to watch the online training module and follow the training manual. During the initiation, the Columbia training acupuncturist will observe each site acupuncturist perform true and sham acupuncture on a volunteer. All acupuncturists will undergo quality assurance checks throughout the study period.

a. The first time the patient arrives for acupuncture: The acupuncturist will read to the patient the instructions. Explain the specific administration times for this protocol. Patients should be directed to report all symptoms and limitations.

SWOG Authorized Document

b. The acupuncturist will be the only participant on the study team that is unblinded to sham and true acupuncture assignment. The acupuncturist will log into the CRAB website to retrieve the assignment after the patient has been registered. They will maintain all blinding to both the patient and the investigators. All eCRF's should be filled out at the time of each visit and sent electronically to the Columbia Coordinating center. An unblinded staff member at CUMC will review everything for accuracy and completeness and will be available for questions by the study acupuncturists regarding data submission of acupuncture visits: Ramona Jayasena, phone 212/304-5579 or e-mail ra2002@columbia.edu.

c. It is very important to review the CRFs after the patient has completed treatment to be sure all of the questions have been answered.

d. If a patient refuses, does not show up or cannot complete the acupuncture for some reason, then this must be documented on the cover sheet and faxed to the Data Operations Center in Seattle as well as the Columbia Coordinating Center (see Section 14.3b).

e. The acupuncturists will participate in a monthly conference call with the Columbia Coordinating Center along with the SWOG Protocol Coordinator to maintain quality control.

16.0 ETHICAL AND REGULATORY CONSIDERATIONS

The following must be observed to comply with Food and Drug Administration regulations for the conduct and monitoring of clinical investigations; they also represent sound research practice:

Informed Consent

The principles of informed consent are described by Federal Regulatory Guidelines (Federal Register Vol. 46, No. 17, January 27, 1981, part 50) and the Office for Protection from Research Risks Reports: Protection of Human Subjects (Code of Federal Regulations 45 CFR 46). They must be followed to comply with FDA regulations for the conduct and monitoring of clinical investigations.

Institutional Review

This study must be approved by an appropriate institutional review committee as defined by Federal Regulatory Guidelines (Ref. Federal Register Vol. 46, No. 17, January 27, 1981, part 56) and the Office for Protection from Research Risks Reports: Protection of Human Subjects (Code of Federal Regulations 45 CFR 46).

Monitoring

This study will be monitored by the Clinical Data Update System (CDUS) Version 3.0. Cumulative CDUS data will be submitted quarterly to CTEP by electronic means. Reports are due January 31, April 30, July 31 and October 31.

Adverse Experiences

There are no agents in this study.

SWOG Authorized Document

16.1 Adverse Event Reporting Requirements

a. Purpose

Adverse event data collection and reporting, which are required as part of every clinical trial, are done to ensure the safety of patients enrolled in the studies as well as those who will enroll in future studies using similar agents. Adverse events are reported in a routine manner at scheduled times during a trial. (Directions for routine reporting are provided in Section 14.0.) Additionally, certain adverse events must be reported in an expedited manner to allow for more timely monitoring of patient safety and care. The following guidelines prescribe expedited adverse event reporting for this protocol. See also Appendix 19.1 for general and background information about expedited reporting.

b. Reporting methods

This study requires that expedited adverse event reporting use the NCI's Adverse Event Expedited Reporting System (AdEERS). The NCI's guidelines for AdEERS can be found at http://ctep.cancer.gov. An AdEERS report must be sent to SWOG Operations Office by electronically submitting the report via the AdEERS Web-based application located at http://ctep.cancer.gov.

In the rare event when internet connectivity is disrupted a 24-hour notification is made to NCI by telephone at 301-897-7497. An electronic report MUST be submitted immediately upon re-establishment of internet connection. Please note that all paper AdEERS forms have been removed from the CTEP website and will NO LONGER be accepted.

c. When to report an event in an expedited manner

When the adverse event requires expedited reporting, submit the report within 10 calendar days of learning of the event.

You may be asked to submit supporting clinical data to the Operations Offices in order to complete the evaluation of the event. If requested, the supporting data should be sent within 5 calendar days by fax to 210-614-0006.

d. Other recipients of adverse event reports

The Operations Office will forward reports and documentation to the appropriate regulatory agencies and drug companies as required. Adverse events determined to be reportable to the Institutional Review Board responsible for oversight of the patient must be reported according to local policy and procedures.

e. Expedited reporting for acupuncture/sham interventions

Reporting requirements for acupuncture intervention are provided in Table 16.1. If there is any question about the reportability of an adverse event or if on-line AdEERS cannot be used, please telephone or email the SAE Program at the Operations Office, 210/614-8808 or adr@swog.org, before preparing the report.

SWOG Authorized Document

Table 16.1. Expedited reporting requirements for adverse events experienced by patients who have received intervention on this study.

**Attribution**	**Grade 4**		**Grade 5**[a]	
	Unexpected	Expected	Unexpected	Expected
Unrelated or Unlikely			**AdEERS**	**AdEERS**
Possible, Probable, Definite	**AdEERS**		**AdEERS**	**AdEERS**

AdEERS: Indicates an expedited report is to be submitted via NCI AdEERS within 10 calendar days of learning of the event[b].

[a] This includes all deaths within 30 days of the last acupuncture/sham intervention, regardless of attribution. Any death that occurs more than 30 days after the last acupuncture/sham intervention and is attributed (possibly, probably, or definitely) to the intervention and is not due to cancer recurrence must be reported according to the instructions above.

[b] Submission of the on-line AdEERS report plus any necessary amendments generally completes the reporting requirements. You may, however, be asked to submit supporting clinical data to the Operations Office in order to complete the evaluation of the event. If requested, the specified data should be sent by fax to 210-614-0006.

SWOG Authorized Document

17.0 BIBLIOGRAPHY

1. Wakeling AE, Valcaccia B, Newboult E, Green LR. Non-steroidal antioestrogens--receptor binding and biological response in rat uterus, rat mammary carcinoma and human breast cancer cells. Journal of Steroid Biochemistry, 20: 111-20, 1984.

2. Cutuli B, Petit JC, Fricker JP, Schumacher C, Velten M, Abecassis J. [Thromboembolic accidents in postmenopausal patients with adjuvant treatment by tamoxifen. Frequency, risk factors and prevention possibilities]. Bulletin du Cancer, 82: 51-6, 1995.

3. Meier CR, Jick H. Tamoxifen and risk of idiopathic venous thromboembolism. British Journal of Clinical Pharmacology, 45: 608-12, 1998.

4. Bernstein L, Deapen D, Cerhan JR, Schwartz SM., Liff J, McGann-Maloney E, Perlman JA, Ford, L. Tamoxifen therapy for breast cancer and endometrial cancer risk. Journal of the National Cancer Institute, 91: 1654-62, 1999.

5. Siiteri P, MacDonald P. Role of extraglandular estrogen in human endocrinology. In: R. Greep and E. Astwood (eds.), Handbook of Physiology, pp. 615-629. Washington, D.C.: American Physiological Society, 1973.

6. Miller WR, Hawkins RA, Forrest AP. Significance of aromatase activity in human breast cancer. Cancer Research, 42, 1982.

7. Nelson LR, Bulun SE. Estrogen production and action. Journal of the American Academy of Dermatology, 45, 2001.

8. Baum M, Budzar AU, Cuzick J, Forbes J, Houghton JH, Klijn JG, Sahmoud T, Group AT. Anastrozole alone or in combination with tamoxifen versus tamoxifen alone for adjuvant treatment of postmenopausal women with early breast cancer: first results of the ATAC randomised trial.[see comment][erratum appears in Lancet 2002 Nov 9;360(9344):1520]. Lancet, 359: 2131-9, 2002.

9. Goss PE, Ingle JN, Martino S, Robert NJ, Muss HB, Piccart MJ, Castiglione M, Tu D, Shepherd LE, Pritchard KI, Livingston RB, Davidson NE, Norton L, Perez EA, Abrams JS, Therasse P, Palmer MJ, Pater JL. A randomized trial of letrozole in postmenopausal women after five years of tamoxifen therapy for early-stage breast cancer.[see comment]. New England Journal of Medicine, 349: 1793-802, 2003.

10. Howell A, Cuzick J, Baum M, Buzdar A, Dowsett,M, Forbes JF, Hoctin-Boes G, Houghton J, Locker GY, Tobias JS Results of the ATAC (Arimidex, Tamoxifen, Alone or in Combination) trial after completion of 5 years' adjuvant treatment for breast cancer. Lancet, 365: 60-2, 2005.

11. Thurlimann B, Keshaviah A, Coates AS, Mouridsen H, Mauriac L, Forbes JF, Paridaens R, Castiglione-Gertsch M, Gelber RD, Rabaglio M, Smith I, Wardly A, Price KN, Goldhirsch A. A comparison of letrozole and tamoxifen in postmenopausal women with early breast cancer. N Engl J Med, 353: 2747-57, 2005.

12. Goss PE, Ingle JN, Martino S, Robert NJ, Muss HB, Piccart MJ, Castiglione M, Tu D, Shepherd LE, Pritchard KI., Livingston RB, Davidson NE, Norton L, Perez EA, Abrams JS, Cameron DA, Palmer MJ, Pater JL. Randomized trial of letrozole following tamoxifen as extended adjuvant therapy in receptor-positive breast cancer: updated findings from NCIC CTG MA.17. J Natl Cancer Inst, 97: 1262-71, 2005.

SWOG Authorized Document

13. Coombes RC, Hall E, Gibson LJ, Paridaens R, Jassem J, Delozier T, Jones SE, Alvarez I, Bertelli G, Ortmann O, Coates AS, Bajetta E, Dodwell D, Coleman RE, Fallowfield LJ, Mickiewicz E, Andersen J, Lonning PE, Cocconi G, Stewart A, Stuart N, Snowdon CF, Carpentieri M, Massimini G, Bliss JM. A randomized trial of exemestane after two to three years of tamoxifen therapy in postmenopausal women with primary breast cancer. N Engl J Med, 350: 1081-92, 2004.

14. Bajetta E, Martinetti A, Zilembo N, Pozzi P, La Torre I, Ferrari L, Seregni E, Longarini R, Salvucci G, Bombardieri E. Biological activity of anastrozole in postmenopausal patients with advanced breast cancer: effects on estrogens and bone metabolism. Annals of Oncology, 13: 1059-66, 2002.

15. Harper-Wynne C, Ross G, Sacks N, Salter J, Nasiri N, Iqbal J, A'Hern R, Dowsett M. Effects of the aromatase inhibitor letrozole on normal breast epithelial cell proliferation and metabolic indices in postmenopausal women: a pilot study for breast cancer prevention. Cancer Epidemiology, Biomarkers & Prevention, 11: 614-21, 2002.

16. Donnellan PP, Douglas SL, Cameron DA, Leonard RC. Aromatase inhibitors and arthralgia.[comment]. Journal of Clinical Oncology, 19: 15, 2001.

17. Hershman DL, Kushi L, Shao T, Buono,D, Kershenbaum A, Tsai W, Neugut A. I. Early discontinuation and non-adherence to adjuvant hormonal therapy in a cohort of 8769 early stage breast cancer patients (In Press). Journal of Clinical Oncology, 2010.

18. Crew KD, Greenlee H, Capodice J, Raptis G, Brafman L, Fuentes D, Sierra A, Hershman DL Prevalence of joint symptoms in postmenopausal women taking aromatase inhibitors for early-stage breast cancer. J Clin Oncol, 25: 3877-83, 2007.

19. Crew KD, Awad D, Brafman L, Fuentes D, Hershman DL. Prospective Evaluation of Joint Symptoms in Postmenopausal Women Initiating Aromatase Inhibitors for Early Stage Breast Cancer. San Antonio Breast Cancer Conference, 2009., Howard, F., and Rios, A. Aromatase inhibitor-associated arthralgia and bone pain: Frequency and characterization in clinical practice. Am Soc Slin Oncol, 2006.

20. Presant CA, Kelly C, Bosserman L, Upadhyaya G, Vakil M, Horns R, Ebrahimi B, Yeon C, Howard F, Rios A. Aromatase inhibitor-associated arthralgia and bone pain: Frequency and characterization in clinical practice. Am Soc Slin Oncol, 2006.

21. Morales L, Neven P, Timmerman D, Christiaens M, Vergote I, Van Limbergen E, Carbonez A., Van Huffel, S., Ameye, L., and Paridaens, R. Acute effects of tamoxifen and third-generation aromatase inhibitors on menopausal symptoms of breast cancer patients. Anti-Cancer Drugs, 15: 753-760, 2004.

22. Morales L, Pans S, Verschueren K, Van Calster B, Paridaens R, Westhovens R, Timmerman D, De Smet L, Vergote I, Christiaens MR, Neven P. Prospective study to assess short-term intra-articular and tenosynovial changes in the aromatase inhibitor-associated arthralgia syndrome. J Clin Oncol, 26: 3147-52, 2008.

23. Dizdar O, Ozcakar L, Malas FU, Harputluoglu H, Bulut N, Aksoy S, Ozisik Y, Altundag K. Sonographic and electrodiagnostic evaluations in patients with aromatase inhibitor-related arthralgia. J Clin Oncol, 27: 4955-60, 2009.

24. Sestak I, Sapunar F, Cuzick J. Aromatase inhibitor-induced carpal tunnel syndrome: results from the ATAC trial. J Clin Oncol, 27: 4961-5, 2009.

25. Sestak I, Cuzick J, Sapunar F, Eastell R, Forbes JF, Bianco AR, Buzdar AU. Risk factors for joint symptoms in patients enrolled in the ATAC trial: a retrospective, exploratory analysis. Lancet Oncol, 9: 866-72, 2008.

SWOG Authorized Document

26. Muslimani AA, Spiro TP, Chaudhry AA, Taylor HC, Jaiyesimi I, Daw HA. Aromatase inhibitor-related musculoskeletal symptoms: is preventing osteoporosis the key to eliminating these symptoms? Clin Breast Cancer, 9: 34-8, 2009.

27. Colomer R, Monzo M, Tusquets I, Rifa J, Baena JM, Barnadas A, Calvo L, Carabantes F, Crespo C, Munoz M, Llombart A, Plazaola A, Artells R, Gilabert M, Lloveras B, Alba E. A single-nucleotide polymorphism in the aromatase gene is associated with the efficacy of the aromatase inhibitor letrozole in advanced breast carcinoma. Clin Cancer Res, 14: 811-6, 2008.

28. Ingle JN. Pharmacogenomics of tamoxifen and aromatase inhibitors. Cancer, 112: 695-9, 2008.

29. Riggs BL, Melton LJ 3rd. The prevention and treatment of osteoporosis.[see comment][erratum appears in N Engl J Med. 1993 Jan 7;328(1):65; author reply 66; PMID: 8416278]. New England Journal of Medicine, 327: 620-7, 1992.

30. Sherwin,BB. Hormones, mood, and cognitive functioning in postmenopausal women. Obstetrics & Gynecology, 87, 1996.

31. Thomas TN, Rhodin JA, Clark L, Garces A, Bryant MA comparison of the anti-inflammatory activities of conjugated estrogens and 17-beta estradiol. Inflammation Research, 52: 452-60, 2003.

32. Ushiyama T, Inoue K, Nishioka J. Expression of estrogen receptor related protein (p29) and estradiol binding in human arthritic synovium. Journal of Rheumatology, 22: 421-6, 1995.

33. Rosner I. A., Manni, A., Malemud, C. J., Boja, B., and Moskowitz, R. W. Estradiol receptors in articular chondrocytes. Biochemical & Biophysical Research Communications, 106: 1378-82, 1982.

34. Dayani N, Corvol MT, Robel P, Eychenne B, Moncharmont B, Tsagris L, Rappaport R. Estrogen receptors in cultured rabbit articular chondrocytes: influence of age. Journal of Steroid Biochemistry, 31: 351-6, 1988.

35. Richmond RS, Carlson CS, Register TC, Shanker G, Loeser RF. Functional estrogen receptors in adult articular cartilage: estrogen replacement therapy increases chondrocyte synthesis of proteoglycans and insulin-like growth factor binding protein 2. Arthritis & Rheumatism, 43: 2081-90, 2000.

36. Kramer PR, Kramer SF, Guan G. 17 beta-estradiol regulates cytokine release through modulation of CD16 expression in monocytes and monocyte-derived macrophages. Arthritis & Rheumatism, 50: 1967-75, 2004.

37. Saklatvala J. Tumour necrosis factor alpha stimulates resorption and inhibits synthesis of proteoglycan in cartilage. Nature, 322: 547-9, 1986.

38. Henderson B, Pettipher ER. Arthritogenic actions of recombinant IL-1 and tumour necrosis factor alpha in the rabbit: evidence for synergistic interactions between cytokines in vivo. Clinical & Experimental Immunology, 75: 306-10, 1989.

39. Wooley PH, Whalen JD, Chapman DL, Berger AE, Richard KA, Aspar DG, Staite,ND. The effect of an interleukin-1 receptor antagonist protein on type II collagen-induced arthritis and antigen-induced arthritis in mice. Arthritis & Rheumatism, 36: 1305-14, 1993.

40. Tsai CL, Liu TK, Chen TJ. Estrogen and osteoarthritis: a study of synovial estradiol and estradiol receptor binding in human osteoarthritic knees. Biochem Biophys Res Commun, 183: 1287-91, 1992.

SWOG Authorized Document

41. Claassen H, Hassenpflug J, Schunke M, Sierralta W, Thole H, Kurz B. Immunohistochemical detection of estrogen receptor alpha in articular chondrocytes from cows, pigs and humans: in situ and in vitro results. Ann Anat, 183: 223-7, 2001.

42. Nilsson LO, Boman A, Savendahl L, Grigelioniene G, Ohlsson C, Ritzen EM., Wroblewski J. Demonstration of estrogen receptor-beta immunoreactivity in human growth plate cartilage. J Clin Endocrinol Metab, 84: 370-3, 1999.

43. Hoegh-Andersen P, Tanko LB, Andersen TL, Lundberg CV, Mo JA, Heegaard AM, Delaisse J M, Christgau S. Ovariectomized rats as a model of postmenopausal osteoarthritis: validation and application. Arthritis Res Ther, 6: R169-80, 2004.

44. da Silva JA, Colville-Nash P, Spector TD, Scott DL, Willoughby D. A. Inflammation-induced cartilage degradation in female rodents. Protective role of sex hormones. Arthritis Rheum, 36: 1007-13, 1993.

45. Dinarello CA. Interleukin-1. Ann N Y Acad Sci, 546: 122-32, 1988.

46. Parker D, Hwa SY, Sambrook PN, Ghosh P. Estrogen replacement therapy mitigates the loss of joint cartilage proteoglycans and bone mineral density induced by ovariectomy and osteoarthritis. APLAR J Rheumatol, 6: 116, 2003.

47. Richmond RS, Carlson CS, Register TC, Shanker G, Loeser RF. Functional estrogen receptors in adult articular cartilage: estrogen replacement therapy increases chondrocyte synthesis of proteoglycans and insulin-like growth factor binding protein 2. Arthritis Rheum, 43: 2081-90, 2000.

48. Blanchard O, Tsagris L, Rappapor, R, Duval-Beaupere G, Corvol M. Age-dependent responsiveness of rabbit and human cartilage cells to sex steroids in vitro. J Steroid Biochem Mol Biol, 40: 711-6, 1991.

49. Guerne PA, Carson DA, Lotz M. IL-6 production by human articular chondrocytes. Modulation of its synthesis by cytokines, growth factors, and hormones in vitro. J Immunol, 144: 499-505, 1990.

50. Claassen H, Schluter M, Schunke M, Kurz B. Influence of 17beta-estradiol and insulin on type II collagen and protein synthesis of articular chondrocytes. Bone, 39: 310-7, 2006.

51. Richette P, Corvol M, Bardin T. Estrogens, cartilage, and osteoarthritis. Joint Bone Spine, 70: 257-62, 2003.

52. Dougherty RH, Rohrer JL, Hayden D, Rubin SD, Leder BZ. Effect of aromatase inhibition on lipids and inflammatory markers of cardiovascular disease in elderly men with low testosterone levels. Clin Endocrinol (Oxf), 62: 228-35, 2005.

53. Azria D, Lamy Y, Belkacemi G, Romieu G, Roux C, Gourgou S, Ozsahin M, Zaman K, Moscardo L, Gligorov J. Letrozole-induced arthralgia: Results of a multicenteric prospective trial exploring clinical parameters and plasma biomarkers. ASCO Breast Cancer Symposium, 2007.

54. Harputluoglu H, Dizdar O, Malas U, Ozcakar L, Bulut N, Ozisik Y, Altundag K. Aromatase inhibitor-associated arthralgia: Prevalence, clinicl and serum parameters among Turkish postmenopausal breast cancer patients. ASCO Annual Meeting 26 J Clin Oncol (May 20 suppl), 2008.

55. Henry N, Harles P, Pshexhetskiy D, Nguyen A, Ahern R, Waxman J, Storniolo A, Hayes D, Flockhart D, Stearns V, Stebbing J. A distinct inflammatory marker pattern in patients with aromatase inhibitor (AI) induceced musculoskeletal symptoms. ASCO Breast Conference, 2008.

SWOG Authorized Document

56. Wluka AE, Davis SR, Bailey M, Stuckey SL, Cicuttini FM. Users of oestrogen replacement therapy have more knee cartilage than non-users. Annals of the Rheumatic Diseases, 60: 332-6, 2001.

57. Sowers MF, Karvonen-Gutierrez CA, Yosef M, Jannausch M, Jiang Y, Garnero P, Jacobson J. Longitudinal changes of serum COMP and urinary CTX-II predict X-ray defined knee osteoarthritis severity and stiffness in women. Osteoarthritis Cartilage, 17: 1609-14, 2009.

58. Garnero P, Piperno M, Gineyts E, Christgau S, Delmas PD, Vignon E. Cross sectional evaluation of biochemical markers of bone, cartilage, and synovial tissue metabolism in patients with knee osteoarthritis: relations with disease activity and joint damage. Ann Rheum Dis, 60: 619-26, 2001.

59. McAlindon TE, Cooper C, Kirwan JR, Dieppe P. A. Knee pain and disability in the community. British Journal of Rheumatology, 31: 189-92, 1992.

60. Neugarten BL, Kraines RJ. "Menopausal Symptoms" in Women of Various Ages. Psychosom Med, 27: 266-73, 1965.

61. Cecil RL, Archer BH. Arthritis of the menopause. JAMA, 84: 75-79, 1925.

62. Felson DT, Nevitt MC. The effects of estrogen on osteoarthritis. Current Opinion in Rheumatology, 10: 269-72, 1998.

63. Sowers MR, McConnell D, Jannausch M, Buyuktur AG, Hochberg M, Jamadar DA. Estradiol and its metabolites and their association with knee osteoarthritis. Arthritis Rheum, 54: 2481-7, 2006.

64. Spector TD, Perry LA, Jubb RW. Endogenous sex steroid levels in women with generalised osteoarthritis. Clin Rheumatol, 10: 316-9, 1991.

65. Cauley JA, Kwoh CK, Egeland G, Nevitt MC, Cooperstein L, Rohay J, Towers, A., and Gutai, J. P. Serum sex hormones and severity of osteoarthritis of the hand. J Rheumatol, 20: 1170-5, 1993.

66. Sowers MF, Hochberg M, Crabbe JP, Muhich A, Crutchfield M, Updike S. Association of bone mineral density and sex hormone levels with osteoarthritis of the hand and knee in premenopausal women. Am J Epidemiol, 143: 38-47, 1996.

67. Gokhale JA, Frenkel SR, Dicesare PE. Estrogen and osteoarthritis. American Journal of Orthopedics, 33: 71-80, 2004.

68. Hannan MT, Felson DT, Anderson JJ, Naimark A, Kannel WB. Estrogen use and radiographic osteoarthritis of the knee in women. The Framingham Osteoarthritis Study. Arthritis Rheum, 33: 525-32, 1990.

69. Spector TD, Nandra D, Hart DJ, Doyle DV. Is hormone replacement therapy protective for hand and knee osteoarthritis in women?: The Chingford Study. Ann Rheum Dis, 56: 432-4, 1997.

70. Nevitt MC, Cummings SR, Lane NE, Hochberg MC, Scott JC, Pressman AR, Genant HK, Cauley JA. Association of estrogen replacement therapy with the risk of osteoarthritis of the hip in elderly white women. Study of Osteoporotic Fractures Research Group. Arch Intern Med, 156: 2073-80, 1996.

71. Uebelhart D, Thonar EJ, Zhang J, Williams JM. Protective effect of exogenous chondroitin 4,6-sulfate in the acute degradation of articular cartilage in the rabbit. Osteoarthritis & Cartilage, 6: 6-13, 1998.

SWOG Authorized Document

72. Prentice RL, Caan B, Chlebowski RT, Patterson R, Kuller LH, Ockene JK, Margolis KL, Limacher MC, Manson JE, Parker LM, Paskett E, Phillips L, Robbins J, Rossouw JE, Sarto GE, Shikany JM, Stefanick ML, Thomson CA, Van Horn L, Vitolins MZ, Wactawski-Wende J, Wallace RB, Wassertheil-Smoller S, Whitlock E, Yano K, Adams-Campbell L, Anderson GL, Assaf AR, Beresford SA, Black HR, Brunner RL, Brzyski RG, Ford L, Gass M, Hays J, Heber D, Heiss G, Hendrix SL, Hsia J, Hubbell FA, Jackson RD, Johnson KC, Kotchen JM, LaCroix AZ, Lane DS, Langer RD, Lasser NL, Henderson MM. Low-fat dietary pattern and risk of invasive breast cancer: the Women's Health Initiative Randomized Controlled Dietary Modification Trial. JAMA, 295: 629-42, 2006.

73. Scudds RJ, Mc DRJ. Empirical evidence of the association between the presence of musculoskeletal pain and physical disability in community-dwelling senior citizens. Pain, 75: 229-35, 1998.

74. Kewman DG, Vaishampayan N, Zald D, Han B. Cognitive impairment in musculoskeletal pain patients. International Journal of Psychiatry in Medicine, 21: 253-62, 1991.

75. Carey TS, Evans A, Hadler N, Kalsbeek W, McLaughlin C, Fryer J. Care-seeking among individuals with chronic low back pain. Spine, 20: 312-7, 1995.

76. Griffin MR, Yared A, Ray WA. Nonsteroidal antiinflammatory drugs and acute renal failure in elderly persons. American Journal of Epidemiology, 151: 488-96, 2000.

77. Walt R, Katschinski B, Logan R, Ashley J, Langman M. Rising frequency of ulcer perforation in elderly people in the United Kingdom. Lancet, 1: 489-92, 1986.

78. Fitzgerald GA. Coxibs and cardiovascular disease. New England Journal of Medicine, 351: 1709-11, 2004.

79. Crew KD, Capodice JL, Greenlee H, Apollo A, Jacobson JS, Raptis G, Blozie K, Sierra A, Hershman DL. Pilot study of acupuncture for the treatment of joint symptoms related to adjuvant aromatase inhibitor therapy in postmenopausal breast cancer patients. J Cancer Surviv, 1: 283-91, 2007.

80. Crew KD, Capodice JL, Greenlee H, Brafman L, Fuentes D, Awad D, Yann Tsai W, Hershman DL. Randomized, blinded, sham-controlled trial of acupuncture for the management of aromatase inhibitor-associated joint symptoms in women with early-stage breast cancer. J Clin Oncol, 28: 1154-60.

81. O'Reilly SC, Muir KR, Doherty M. Screening for pain in knee osteoarthritis: which question? Annals of the Rheumatic Diseases, 55: 931-3, 1996.

82. Weiner DK, Distell B, Studenski S, Martinez S, Lomasney L, Bongiorni D. Does radiographic osteoarthritis correlate with flexibility of the lumbar spine? Journal of the American Geriatrics Society, 42: 257-63, 1994.

83. Hochberg MC, Altman RD, Brandt KD, Clark BM, Dieppe PA, Griffin MR, Moskowitz RW, Schnitzer TJ. Guidelines for the medical management of osteoarthritis. Part II. Osteoarthritis of the knee.American College of Rheumatology.[see comment]. Arthritis & Rheumatism, 38: 1541-6, 1995.

84. Lane NE, Thompson J.M. Management of osteoarthritis in the primary-care setting: an evidence-based approach to treatment. American Journal of Medicine, 103: 29, 1997.

85. Rao JK, Mihaliak K, Kroenke K, Bradley J, Tierney WM, Weinberger M. Use of complementary therapies for arthritis among patients of rheumatologists. Annals of Internal Medicine, 131: 409-16, 1999.

SWOG Authorized Document

86. Helms J. Acupuncture Energetics: A Clinical Approach for Physicians. Berkley, CA: Medical Acupuncture Publishers, 1995.

87. Kaptchuk TJ. Acupuncture: theory, efficacy, and practice. Ann Intern Med, 136: 374-83, 2002.

88. Ahn AC, Colbert AP, Anderson BJ, Martinsen OG, Hammerschlag R, Cina S, Wayne PM, Langevin HM. Electrical properties of acupuncture points and meridians: a systematic review. Bioelectromagnetics, 29: 245-56, 2008.

89. Hui KK. Nixon EE, Vangel MG, Liu J, Marina O, Napadow V, Hodge SM, Rosen BR, Makris N, Kennedy DN. Characterization of the "deqi" response in acupuncture. BMC Complement Altern Med, 7: 33, 2007.

90. Feinberg AP, Vogelstein B. Hypomethylation distinguishes genes of some human cancers from their normal counterparts. Nature, 301: 89-92, 1983.

91. Takeda W, Wessel J. Acupuncture for the treatment of pain of osteoarthritic knees. Arthritis Care & Research, 7: 118-22, 1994.

92. Christensen BV, Iuhl IU, Vilbek H, Bulow HH, Dreijer NC, Rasmussen HF. Acupuncture treatment of severe knee osteoarhrosis. A long-term study. Acta Anaesthesiologica Scandinavica, 36: 519-25, 1992.

93. Ezzo J, Hadhazy V, Birch S, Lao L, Kaplan G, Hochberg M, Berman B. Acupuncture for osteoarthritis of the knee: a systematic review. Arthritis & Rheumatism, 44: 819-25, 2001.

94. Vas J, Mendez C, Perea-Milla E, Vega E, Panadero M, Leon J, MA B, Gaspar O, Sanchez-Rodriguez F, Aguilar I, Jurado R. Acupuncture as a complementary therapy to the pharmacological treatment of osteoarhritis of the knee: randomised controlled trial. British Medical Journal, doi:10.1136: 1-5, 2004.

95. Brinkhaus B, Becker-Witt C, Jena S, Linde K, Streng A, Wagenpfeil S, Irnich D, Hummelsberger J, Melchart D, Willich SN. Acupuncture Randomized Trials (ART) in patients with chronic low back pain and osteoarthritis of the knee - design and protocols. Forschende Komplementarmedizin und Klassische Naturheilkunde, 10: 185-91, 2003.

96. Witt C, Brinkhaus B, Jena S, Linde K, Streng A, Wagenpfeil S, Hummelsberger J, Walther HU, Melchart D, Willich SN. Acupuncture in patients with osteoarthritis of the knee: a randomised trial. Lancet, 366: 136-43, 2005.

97. Foster NE, Thomas E, Barlas P, Hill J.C, Young J, Mason E, Hay EM. Acupuncture as an adjunct to exercise based physiotherapy for osteoarthritis of the knee: randomised controlled trial. BMJ, 335: 436, 2007.

98. Berman AH, Lundberg U, Krook AL, Gyllenhammar C. Treating drug using prison inmates with auricular acupuncture: a randomized controlled trial. Journal of Substance Abuse Treatment, 26: 95-102, 2004.

99. Szyf M, Pakneshan P, Rabbani S. A. DNA methylation and breast cancer. Biochem Pharmacol, 68: 1187-97, 2004.

100. Alimi D, Rubino C, Pichard-Leandri E, Fermand-Brule S, Dubreuil-Lemaire ML, Hill C. Analgesic effect of auricular acupuncture for cancer pain: a randomized, blinded, controlled trial. Journal of Clinical Oncology, 21: 4120-6, 2003.

101. Wu Y, Fisher W. Practical Therapeutics of Traditional Chinese Medicine. Brookline, MA: Paradigm Publications, 1997.

SWOG Authorized Document

102. Maciocia G. The Practice of Chinese Medicine. pp. 561-605: Churchill Livingston, 1994.

103. Ellis A, Wiseman N, Boss K. Fundamentals of Chinese Acupuncture. Brookline, MA: Paradigm Publications, 1991.

104. Barnes PM, Powell-Griner E, McFann K, Nahin RL. Complementary and alternative medicine use among adults: United States, 2002. Advance Data, 343: 1-19, 2004.

105. Lengacher CA, Bennett MP, Kip KE, Keller R, LaVance MS, Smith LS, Cox CE. Frequency of use of complementary and alternative medicine in women with breast cancer. Oncology Nursing Forum. Online., 29: 1445-52, 2002.

106. Gray RE, Fitch M, Goel V, Franssen E, Labrecque M. Utilization of complementary/alternative services by women with breast cancer. Journal of Health & Social Policy, 16: 75-84, 2003.

107. Navo MA, Phan J, Vaughan C, Palmer JL, Michaud L, Jones KL, Bodurka DC, Basen-Engquist K, Hortobagyi GN, Kavanagh JJ, Smith JA. An assessment of the utilization of complementary and alternative medication in women with gynecologic or breast malignancies. Journal of Clinical Oncology, 22: 671-7, 2004.

108. Ashikaga T, Bosompra K, O'Brien P, Nelson L. Use of complimentary and alternative medicine by breast cancer patients: prevalence, patterns and communication with physicians. Supportive Care in Cancer, 10: 542-8, 2002.

109. DiGianni LM, Garber JE, Winer EP. Complementary and alternative medicine use among women with breast cancer. Journal of Clinical Oncology, 20: 15, 2002.

110. Patterson RE, Neuhouser ML, Hedderson MM, Schwartz SM, Standish LJ, Bowen DJ, Marshall LM. Types of alternative medicine used by patients with breast, colon, or prostate cancer: predictors, motives, and costs. Journal of Alternative & Complementary Medicine, 8: 477-85, 2002.

111. Roscoe JA, Morrow GR, Hickok JT, Bushunow P, Pierce HI, Flynn PJ, Kirshner JJ, Moore DF, Atkins JN. The efficacy of acupressure and acustimulation wrist bands for the relief of chemotherapy-induced nausea and vomiting. A University of Rochester Cancer Center Community Clinical Oncology Program multicenter study. J Pain Symptom Manage, 26: 731-42, 2003.

112. Johnstone PA, Niemtzow RC, Riffenburgh RH. Acupuncture for xerostomia: clinical update. Cancer, 94: 1151-6, 2002.

113. Johnstone PA, Peng YP, May BC, Inouye WS, Niemtzow RC. Acupuncture for pilocarpine-resistant xerostomia following radiotherapy for head and neck malignancies. Int J Radiat Oncol Biol Phys, 50: 353-7, 2001.

114. Wong RK., Jones GW, Sagar SM, Babjak,AF, Whelan T. A Phase I-II study in the use of acupuncture-like transcutaneous nerve stimulation in the treatment of radiation-induced xerostomia in head-and-neck cancer patients treated with radical radiotherapy. Int J Radiat Oncol Biol Phys, 57: 472-80, 2003.

115. Chen C, Zhang Z Li H, et al. Electroacupuncture on Zusangli (ST36) to reduce chemotherapy induced toxicity. Xin Zhong Yi, 36: 46-47, 2004.

116. Lu W. Acupuncture for side effects of chemoradiation therapy in cancer patients. Semin Oncol Nurs, 21: 190-5, 2005.

SWOG Authorized Document

117. Walker EM, Rodriguez AI, Kohn B, Ball RM, Pegg J, Pocock JR, Nunez R, Peterson E, Jakary S. Levine RA. Acupuncture Versus Venlafaxine for the Management of Vasomotor Symptoms in Patients With Hormone Receptor-Positive Breast Cancer: A Randomized Controlled Trial. J Clin Oncol, 2009.

118. Han J. Central neurotransmitters and acupuncture analgesia. In: B. Pomeranz and G. Stux (eds.), Scientific Bases of Acupunture, pp. 7-33. New York: Springer-Verlag, 1989.

119. Clement-Jones V, McLoughlin L, Tomlin S, Besser GM, Rees LH, Wen HL. Increased beta-endorphin but not met-enkephalin levels in human cerebrospinal fluid after acupuncture for recurrent pain. Lancet, 2: 946-9, 1980.

120. Pomeranz B, Chiu D. Naloxone blockade of acupuncture analgesia: endorphin implicated. Life Sciences, 19: 1757-62, 1976.

121. Murotani T, Ishizuka T, Nakazawa H, Wang X, Mori K, Sasaki K, Ishida T, Yamatodani A. Possible involvement of histamine, dopamine, and noradrenalin in the periaqueductal gray in electroacupuncture pain relief. Brain Res, 1306: 62-8.

122. Hans J. Neuroscience Letters, 361: 258-261, 1994.

123. Mendelson G. The possible role of enkephalin in the mechanism of acupuncture analgesia in man. Med Hypotheses, 3: 144-5, 1977.

124. Han JS. Acupuncture and endorphins. Neurosci Lett, 361: 258-61, 2004.

125. Han JS. Acupuncture: neuropeptide release produced by electrical stimulation of different frequencies. Trends Neurosci, 26: 17-22, 2003.

126. Liu X, Zhu B, Zhang SX. Relationship between electroacupuncture analgesia and descending pain inhibitory mechanism of nucleus raphe magnus. Pain, 24: 383-96, 1986.

127. Ho SC, Chiu JH, Yeh TC, Hsieh JC, Cheng HC, Cheng H, Ho LT. Quantification of electroacupuncture-induced neural activity by analysis of functional neural imaging with monocrystalline iron oxide nanocolloid enhancement. Am J Chin Med, 36: 493-504, 2008.

128. Kim WS, Kim IS, Kim SJ, Wei P, Hyung Choi D, Han TR. Effect of electroacupuncture on motor recovery in a rat stroke model during the early recovery stage. Brain Res, 1248: 176-83, 2009.

129. Guo ZL, Moazzami AR, Longhurst JC. Electroacupuncture induces c-Fos expression in the rostral ventrolateral medulla and periaqueductal gray in cats: relation to opioid containing neurons. Brain Res, 1030: 103-15, 2004.

130. Ma F, Xie H, Dong ZQ, Wang YQ, Wu GC. Effects of electroacupuncture on orphanin FQ immunoreactivity and preproorphanin FQ mRNA in nucleus of raphe magnus in the neuropathic pain rats. Brain Res Bull, 63: 509-13, 2004.

131. Scharf HP, Mansmann U, Streitberger K, Witte S, Kramer J, Maier C, Trampisch HJ, Victor N. Acupuncture and knee osteoarthritis: a three-armed randomized trial. Ann Intern Med, 145: 12-20, 2006.

132. Kaptchuk TJ, Stason WB, Davis RB, Legedza AR, Schnyer RN, Kerr CE, Stone DA, Nam BH, Kirsch I, Goldman RH. Sham device v inert pill: randomised controlled trial of two placebo treatments. Bmj, 332: 391-7, 2006.

133. McManus CA, Schnyer RN, Kong J, Nguyen LT, Hyun Nam B, Goldman R, Stason WB, Kaptchuk TJ. Sham acupuncture devices--practical advice for researchers. Acupunct Med, 25: 36-40, 2007.

SWOG Authorized Document

134. Moffet HH. Sham acupuncture may be as efficacious as true acupuncture: a systematic review of clinical trials. J Altern Complement Med, 15: 213-6, 2009.

135. Linde K, Witt CM, Streng A, Weidenhammer W, Wagenpfeil S, Brinkhaus B, Willich SN, Melchart D. The impact of patient expectations on outcomes in four randomized controlled trials of acupuncture in patients with chronic pain. Pain, 128: 264-71, 2007.

136. Guidance for industry: patient-reported outcome measures: use in medical product development to support labeling claims: draft guidance. Health Qual Life Outcomes, 4: 79, 2006.

137. Cleeland CS, Ryan KM. Pain assessment: global use of the Brief Pain Inventory. Annals of the Academy of Medicine, Singapore, 23: 129-38, 1994.

138. Jensen M.P, Turner JA, Romano JM, Fisher LD. Comparative reliability and validity of chronic pain intensity measures. Pain, 83: 157-62, 1999.

139. Bellamy N, Buchanan WW, Goldsmith CH, Campbell J, Stitt L.W. Validation study of WOMAC: a health status instrument for measuring clinically important patient relevant outcomes to antirheumatic drug therapy in patients with osteoarthritis of the hip or knee. Journal of Rheumatology, 15: 1833-40, 1988.

140. Sautner J, Andel I, Rintelen B, Leeb BF. Development of the M-SACRAH, a modified, shortened version of SACRAH (Score for the Assessment and Quantification of Chronic Rheumatoid Affections of the Hands). Rheumatology (Oxford), 43: 1409-13, 2004.

141. Fraser A, Vallow J, Preston A, Cooper RG. Predicting 'normal' grip strength for rheumatoid arthritis patients. Rheumatology (Oxford), 38: 521-8, 1999.

142. Mathias S, Nayak US, Isaacs B. Balance in elderly patients: the "get-up and go" test. Arch Phys Med Rehabil, 67: 387-9, 1986.

143. Mao JJ, Bruner DW, Stricker C, Farrar JT, Xie SX, Bowman MA, Pucci D, Han X, DeMichele A. Feasibility trial of electroacupuncture for aromatase inhibitor--related arthralgia in breast cancer survivors. Integr Cancer Ther, 8: 123-9, 2009.

144. Crew KD, Capodice J, Greenlee H, Brafman L, Fuentes D, Awad D, Tsai W, Hershman DL. Randomized, blinded, sham-controlled trial of acupuncture for the management of aromatase inhibitor-associated joint symptoms in women with early stage breast cancer (In Press). Journal of Clinical Oncology, 2010.

145. Anonymous. NIH Consensus Conference. Acupuncture. JAMA, 280: 1518-24, 1998.

146. Seeman TE, Charpentier PA, Berkman LF, Tinetti ME, Guralnik JM, Albert M, Blazer D, Rowe JW. Predicting changes in physical performance in a high-functioning elderly cohort: MacArthur studies of successful aging. J Gerontol, 49: M97-108, 1994.

147. Weiner DK, Ernst E. Complementary and alternative approaches to the treatment of persistent musculoskeletal pain. Clinical Journal of Pain, 20: 244-55, 2004.

148. Farrar JT, Young JP Jr, LaMoreaux L, Werth JL, Poole RM. Clinical importance of changes in chronic pain intensity measured on an 11-point numerical pain rating scale. Pain, 94: 149-58, 2001.

149. Yost KJ, Cella D, Chawla A, Holmgren E, Eton DT, Ayanian JZ, West DW. Minimally important differences were estimated for the Functional Assessment of Cancer Therapy-Colorectal (FACT-C) instrument using a combination of distribution- and anchor-based approaches. J Clin Epidemiol, 58: 1241-51, 2005.

SWOG Authorized Document

150. Schnyer RN, Allen JJ. Bridging the gap in complementary and alternative medicine research: manualization as a means of promoting standardization and flexibility of treatment in clinical trials of acupuncture. J Altern Complement Med, 8: 623-34, 2002.

151. Xinnong C. Chinese Acupuncture and Moxibustion. pp. 134-226. Beijing: Foreign Languages Press, 1999.

152. Chen X. Chinese Acupuncture and Moxibustion. pp. 134-226. Beijing: Foreign Languages Press, 1999.

153. Ernst E, White AR. Prospective studies of the safety of acupuncture: a systematic review. Am J Med, 110: 481-5, 2001.

154. Vincent C. The safety of acupuncture. BMJ, 323: 467-8, 2001.

155. Fallowfield LJ, Leaity SK, Howell A, Benson S, Cella D. Assessment of quality of life in women undergoing hormonal therapy for breast cancer: validation of an endocrine symptom subscale for the FACT-B. Breast Cancer Res Treat, 55: 189-99, 1999.

156. van der Waal J, Terwee C, van der Windt D, Bouter L, Dekker J. Health-related and overall quality of life of patients with chronic hip and knee complaints in general practice. Quality of Life Research, 14: 795-803, 2005.

157. Mitchell C, Walker J, Walters S, Morgan A, Binns T, Mathers N. Costs and effectiveness of pre- and post-operative home physiotherapy for total knee replacement. Journal of Evaluation in Clinical Practice, *11:* 283-92, 2005.

158. Jaarsma R, Ongkiehong B, Gruneberg C, Verdonschot N, Duysens J, van Kampen A. Compensation for rotational malalignment after intramedullary nailing for femoral shaft fractures. An analysis by plantar pressure measurements during gait. Injury, *35:* 1270-78, 2004.

159. Gangji V, Hauzeur J, Matos C, De Maertelaer V, Toungouz M, Lambermont M. Treatment of osteonecrosis of the femoral head with implantation of autologous bone-marrow cells. Journal of Bone & Joint Surgery - American Volume, *86-A:* 1153-60, 2004.

SWOG Authorized Document

18.0 MASTER FORMS SET

18.1 The Model Informed Consent Form is included in this section, preceded by "Notes for Local Institution Consent Form Authors" and "Notes for Local Investigators." The study - as well as the local consent form meeting the guidelines noted in these documents - must be reviewed and approved by the Institutional Review Board prior to registration and treatment of patients on this study.

18.2 This section includes copies of all data forms which must be completed for this study. These include:

a. **S1200** Registration Worksheet (Form #39748)

b. **S1200** Prestudy Form (Form #41314)

c. **S1200** Cover Sheet for Patient-Completed Questionnaires (Form #19445)

d. **S1200** Brief Pain Inventory Short Form (BPI-SF) (Form #40963)

e. **S1200** Western Ontario and McMaster Universities Osteoarthritis (WOMAC) Index (Version 3.1) (Form #33579)

f. **S1200** Modified-Score for the Assessment and Quantification of Chronic Rheumatoid Affections of the Hands (M-SACRAH) (Form #48347)

g. **S1200** PROMIS Pain Impact-Short Form (PROMIS PI-SF) (Form #28494)

h. **S1200** FACT-ES Trial Outcome Index (Version 4) (Form #13659)

i. **S1200** Aromatase Inhibitor Usage Form (Form #39879)

j. **S1200** Functional Testing (Form #37709)

k. **S1200** Adherence to Acupuncture Form (Form #6044)

l. **S1200** Adverse Event Summary Form (Form #5183)

m. **S1200** Supplemental Agents Reporting Form (Form #9413)

n. **S1200** Assessment of Blinding (Form #20574)

o. **S1200** Off Protocol Notice (Form #9707)

p. Follow-Up Form (Form #64587)

q. Notice of Death (Form #49467)

SWOG Authorized Document

Informed Consent Model for <u>S1200</u>

*NOTES FOR LOCAL INSTITUTION INFORMED CONSENT AUTHORS:

This model informed consent form has been reviewed by the DCT/NCI and is the official consent document for this study. Local IRB changes to this document are allowed. (Institutions should attempt to use sections of this document that are in bold type in their entirety.) Editorial changes to these sections may be made as long as they do not change information or intent. If the institutional IRB insists on making deletions or more substantive modifications to the risks or alternatives sections, they may be justified in writing by the investigator and approved by the IRB. Under these circumstances, the revised language, justification and a copy of the IRB minutes must be forwarded to the SWOG Operations Office for approval before a patient may be registered to this study.

Please particularly note that the questions related to banking of specimens for future study are in bolded type and may not be changed in any way without prior approval from the SWOG Operations Office.

Readability Statistics:	
Flesch Reading Ease	<u>62</u> (targeted above 55)
Flesch-Kincaid Grade Level	<u>8.5</u> (targeted below 8.5)

- Instructions and examples for informed consent authors are in *[italics]*.
- A blank line, __________, indicates that the local investigator should provide the appropriate information before the document is reviewed with the prospective research patient.
- The term "study doctor" has been used throughout the model because the local investigator for a cancer treatment trial is a physician. If this model is used for a trial in which the local investigator is not a physician, another appropriate term should be used instead of "study doctor".
- The dates of protocol updates in the header and in the text of the consent is for reference to this model only and should not be included in the informed consent form given to the prospective research patient.
- The local informed consent must state which parties may inspect the research records. This includes the NCI, NCCAM, Office of Research on Women's Health (ORWH), any companies or grantors that are providing study support (these will be listed in the protocol's model informed consent form) and SWOG.

  "SWOG" must be listed as one of the parties that may inspect the research records in all protocol consent forms for which patient registration is being credited to SWOG. This includes consent forms for studies where all patients are registered directly through the SWOG Data Operations Office, all intergroup studies for which the registration is being credited to SWOG (whether the registration is through the SWOG Data Operations Office or directly through the other group), as well as consent forms for studies where patients are registered via CTSU and the registration is credited to SWOG.

SWOG Authorized Document

- When changes to the protocol require revision of the informed consent document, the IRB should have a system that identifies the revised consent document, in order to preclude continued use of the older version and to identify file copies. An appropriate method to identify the current version of the consent is for the IRB to stamp the final copy of the consent document with the approval date. The stamped consent document is then photocopied for use. Other systems of identifying the current version of the consent such as adding a version or approval date are allowed as long as it is possible to determine during an audit that the patient signed the most current version of the consent form.

*NOTES FOR LOCAL INVESTIGATORS:

- The goal of the informed consent process is to provide people with sufficient information for making informed choices. The informed consent form provides a summary of the clinical study and the individual's rights as a research patient. It serves as a starting point for the necessary exchange of information between the investigator and potential research patient. This model for the informed consent form is only one part of the larger process of informed consent. For more information about informed consent, review the "Recommendations for the Development of Informed Consent Documents for Cancer Clinical Trials" prepared by the Comprehensive Working Group on Informed Consent in Cancer Clinical Trials for the National Cancer Institute. The Web site address for this document is http://cancer.gov/clinicaltrials/understanding/simplification-of-informed-consent-docs/
- A blank line, __________, indicates that the local investigator should provide the appropriate information before the document is reviewed with the prospective research patient.
- Suggestion for Local Investigators: An NCI pamphlet explaining clinical trials is available for your patients. The pamphlet is titled: "If You Have Cancer…What You Should Know about Clinical Trials". This pamphlet may be ordered on the NCI Web site at *https://cissecure.nci.nih.gov/ncipubs* or call 1-800-4- CANCER (1-800-422-6237) to request a free copy.
- Optional feature for Local Investigators: Reference and attach drug sheets, pharmaceutical information for the public, or other material on risks. Check with your local IRB regarding review of additional materials.

*These notes for authors and investigators are instructional and should not be included in the informed consent form given to the prospective research patient.

SWOG Authorized Document

S1200, "A Randomized Blinded Sham- and Waitlist-Controlled Trial of Acupuncture for Joint Symptoms Related to Aromatase Inhibitors in Women with Early Stage Breast Cancer"

This is a clinical trial, a type of research study. Your study doctor will explain the clinical trial to you. Clinical trials include only people who choose to take part. Please take your time to make your decision about taking part. You may discuss your decision with your friends and family. You can also discuss it with your health care team. If you have any questions, you can ask your study doctor for more explanation.

You are being asked to take part in this study because you are a postmenopausal woman currently taking an aromatase inhibitor for the treatment of early stage breast cancer and experiencing joint pain or stiffness. Aromatase inhibitors are a type of hormonal therapy for breast cancer, including anastrozole (Arimidex), letrozole (Femara), and exemestane (Aromasin).

Who is doing this study?

SWOG is sponsoring this trial. SWOG is an adult cancer clinical trials organization. SWOG is funded through the National Cancer Institute, and its network consists of almost four thousand physicians at almost three hundred institutions throughout the United States. Your study doctor has met all requirements to be a member of SWOG and to perform National Cancer Institute-funded research through this Group. This particular study is also being supported by the National Center for Complementary and Alternative Medicine and by the Office for Research on Women's Health.

Why is this study being done?

The purpose of this study is to test whether 12-week use of acupuncture can be used to ease joint pain or stiffness related to aromatase inhibitors for breast cancer treatment. We want to find out what effects, good and/or bad, it has on you and your joint symptoms.

Acupuncture is a traditional Chinese medical treatment which involves the use of very small, thin needles to stimulate specific points in the body. Previous studies have found a benefit of acupuncture for the treatment of knee and back pain. This study will test whether acupuncture can reduce joint pain associated with aromatase inhibitor therapy for breast cancer.

How many people will take part in the study?

About 228 people will take part in this study.

SWOG Authorized Document

What will happen if I take part in this research study?

Before you begin the study …

You will need to have the following exams, tests or procedures to find out if you can be in the study. These exams, tests or procedures are part of regular health care for postmenopausal women and may be done even if you do not join the study. If you have had some of them recently, they may not need to be repeated. This will be up to your study doctor.

- Medical history and physical exam
- Blood work to check your menopausal status (if necessary).
- You will be asked to take one questionnaire that will ask you to rate your joint pain and stiffness.

During the study …

If the exams, tests and procedures show that you can be in the study, and you choose to take part, then you will need the following procedure.

Randomization

You will be "randomized" into one of the study groups described below. Randomization means that you are put into a group by chance. A computer program will place you in one of the study groups. Neither you nor your doctor can choose the group you will be in.

You will have a 50% chance of receiving true acupuncture, a 25% chance of receiving sham acupuncture (light acupuncture at non-acupuncture points), and a 25% chance of being in the waitlist control group (you will wait 24 weeks before having the choice to receive true acupuncture). You will not be told whether you are receiving either true or sham acupuncture. Only the acupuncturist and staff members at SWOG and Columbia University will be aware of the acupuncture assignment. These are described below.

If you are in the true or sham acupuncture groups, you will be scheduled for 45-minute acupuncture sessions given twice a week for six weeks, then weekly for another six weeks for a total of twelve weeks. If you are in the waitlist control group, you will not receive true acupuncture until after the 24-week evaluation, if you choose. No matter what group you are assigned to after 24 weeks you will receive 10 free vouchers for true acupuncture. You will be allowed to take certain pain medications as necessary, such as acetaminophen (Tylenol) and anti-inflammatory drugs (such as aspirin, ibuprofen, Celebrex). For the duration of the study, you will be asked not to take steroids or narcotics (opiates) for pain relief or start new treatments for pain relief, if possible. The study will take fifty-two weeks to complete. Follow-up visits will be scheduled at six weeks, twelve weeks, twenty-four weeks, and fifty-two weeks.

SWOG Authorized Document

You will complete the following questionnaires and procedures after randomization.

- Questionnaires- Six self-administered questionnaires (which will take about 30 minutes to complete) will be given at the beginning of the study to collect information about how you are feeling physically during your treatment and how you are performing your daily activities. You will be asked to rate joint pain and stiffness, and will be asked to respond to questions regarding aromatase inhibitors and pain medications. If any questions make you feel uncomfortable, you may skip those questions and not give an answer. We will do our best to make sure that your personal information will be kept private.
- You will be tested for hand grip strength and an activity called "Timed Get Up and Go". The interviewer will ask you to take a short walk of 10 feet then measure the time it takes you to do so (~15 minutes to complete).
- Assess the amount and how often you have taken pain medications.
- Blood and urine samples – Blood (about 2 tablespoons) and urine (about 1/3 of a cup) will be taken before starting the study intervention. The blood and urine samples are not part of the usual medical care. The blood and urine samples will be submitted to look at hormone levels, specific biomarker (biological molecule found in blood that is a sign of a normal or abnormal process, or of a condition or disease), inflammatory markers, break down products from the aromatase inhibitors, and DNA (genetic) studies. These evaluations will be done at the end of the entire study and will not be part of your medical record. You will be asked if remaining blood and urine from the samples can be kept for future research purposes. Please see separate questions at the end of this document.

During telephone interviews at 2 week and 4 weeks …

The medical team or staff will record:

- Your acupuncture schedule (if applicable)
- Side effects you may be having
- Pain treatments you are taking

During study visits at 6, 12, 24 and 52 weeks …

You will have the following procedures:

- Questionnaires- Six self-administered questionnaires (which will take about 30 minutes to complete) will be given at six and twelve weeks to collect information about how you are feeling physically during your treatment and how you are performing your daily activities. You will be asked to rate joint pain and stiffness. You will also give your opinion about whether you were receiving true acupuncture or sham acupuncture (if applicable). If any questions make you feel uncomfortable, you may skip those questions and not give an answer. We will do our best to make sure that your personal information will be kept private.

SWOG Authorized Document

- Functional testing for hand grip strength and an activity called "Timed Get Up and Go" during which the interviewer will ask you to take a short walk of 10 feet then measure the time it takes you to do so (~15 minutes to complete)
- Blood and urine samples – Blood (about 1.5 tablespoons) and urine (about 1/3 of a cup) will be taken at six, twelve and twenty-four weeks. Urine samples only will be taken at 52 weeks. The blood and urine samples are not part of the usual medical care. The blood and urine samples will be submitted to look at hormone levels and inflammatory markers. These results will not be made available to you or your study doctor. You will be asked if remaining blood and urine from the samples can be kept for future research purposes. Please see separate consent form at the end of this document.

The medical team or staff will record:

- Your acupuncture schedule (if applicable)
- Side effects you may be having (at Weeks 6 and 12)
- Pain treatments you are receiving
- Additional acupuncture treatments received outside of the study (at Weeks 24 and 52)

During telephone interviews at 16 weeks and 20 weeks …

The medical team or staff will record:

- Your rating of joint pain and stiffness
- Pain treatments you are receiving

At 24 weeks, all 3 groups will receive vouchers for 10 true acupuncture sessions.

How long will I be in the study?

You will be asked to take part in the study for about 52 weeks.

Can I stop being in the study?

Yes. You can decide to stop at any time. Tell the study doctor if you are thinking about stopping or decide to stop.

The study doctor may stop you from taking part in this study at any time if he/she believes it is in your best interest or if the study is stopped.

SWOG Authorized Document

What side effects or risks can I expect from being in the study?

You may have side effects while on the study. Everyone taking part in the study will be watched carefully for any side effects. However, doctors don't know all the side effects that may happen. Side effects may be mild or very serious. Your health care team may give you medicines to help lessen side effects. Many side effects go away soon after you stop the acupuncture intervention. In some cases, side effects can be serious, long lasting, or may never go away.

You should talk to your study doctor and acupuncturist about any side effects that you have while taking part in the study.

Risks and side effects related to acupuncture include those which are:

Likely (> 20%)

- **Aching, warm or tingling sensation from needling**

Less Likely (~5%)

- **Needle shock or feeling 'faint' from needle insertion. If this should occur, then the acupuncture needles will be removed and you will be laid flat on the examination table for about 15 minutes or until the symptoms subside.**
- **Bruising and minor bleeding**

Rarely (< 1%)

- **Infection**

Are there benefits to taking part in the study?

If you agree to take part in this study, there may or may not be direct medical benefit to you. We hope the information learned from this study will benefit other patients with joint symptoms associated with aromatase inhibitor therapy for breast cancer treatment in the future.

What other choices do I have if I do not take part in this study?

Your other choices may include:

- **Getting treatment without being in a study**
- **Taking part in another study**
- **Getting no treatment**

Talk to your doctor about your choices before you decide if you will take part in this study.

SWOG Authorized Document

Will my medical information be kept private?

We will do our best to make sure that the personal information in your medical record will be kept private. However, we cannot guarantee total privacy. Your personal information may be given out if required by law. If information from this study is published or presented at scientific meetings, your name and other personal information will not be used.

Organizations that may look at and/or copy your medical records for research, quality assurance, and data analysis include:

- SWOG
- The National Cancer Institute (NCI), National Center for Complementary and Alternative Medicine (NCCAM), Office for Research on Women's Health (ORWH) and other government agencies, like the Food and Drug Administration (FDA), involved in keeping research safe for people

What are the costs of taking part in this study?

Taking part in this study may lead to added costs that you or your health insurance plan may be responsible for. Please ask about any expected added costs or insurance problems.

All tests and procedures which are not part of routine clinical care, including the blood and urine tests, will be covered by the study. The acupuncture sessions will be provided free of charge.

Regardless of study arm you are assigned, you will be offered a voucher for ten free true acupuncture sessions to be used off-study at your convenience after the 24-week visit.

For more information on clinical trials and insurance coverage, you can visit the National Cancer Institute's Web site at http://cancer.gov/clinicaltrials/understanding/insurance-coverage. You can print a copy of the "Clinical Trials and Insurance Coverage" information from this Web site.

Another way to get the information is to call 1-800-4-CANCER (1-800-422-6237) and ask them to send you a free copy.

What happens if I am injured because I took part in this study?

It is important that you tell your study doctor, __________________ *[investigator's name(s)],* if you feel that you have been injured because of taking part in this study. You can tell the doctor in person or call him/her at __________________ *[telephone number].*

You will get medical treatment if you are injured as a result of taking part in this study. You and/or your health plan will be charged for this treatment. The study will not pay for medical treatment.

SWOG Authorized Document

What are my rights if I take part in this study?

Taking part in this study is your choice. You may choose either to take part or not to take part in the study. If you decide to take part in this study, you may leave the study at any time. No matter what decision you make, there will be no penalty to you and you will not lose any of your regular benefits. Leaving the study will not affect your medical care. You can still get your medical care from our institution.

In the case of injury resulting from this study, you do not lose any of your legal rights to seek payment by signing this form.

Who can answer my questions about the study?

You can talk to your study doctor about any questions or concerns you have about this study. Contact your study doctor __________________ *[name(s)]* at __________________ *[telephone number].*

For questions about your rights while taking part in this study, call the ________________________ *[name of center]* Institutional Review Board (a group of people who review the research to protect your rights) at __________________ *(telephone number). [Note to Local Investigator: Contact information for patient representatives or other individuals in a local institution who are not on the IRB or research team but take calls regarding clinical trial questions can be listed here.]*

Where can I get more information?

You may call the National Cancer Institute's Cancer Information Service at:

1-800-4-CANCER (1-800-422-6237) or TTY: 1-800-332-8615

You may also visit the NCI Web site at http://cancer.gov/

- **A description of this clinical trial will be available on http://www.ClinicalTrials.gov. This Web site will not include information that can identify you. At most, the Web site will include a summary of study results. You can search this Web site at any time.**

[Note to Informed Consent Authors: the above paragraph complies with the new FDA regulation found at 21 CFR 50.25(c) and must be included verbatim in all informed consent documents. The text in this paragraph cannot be revised.]

- For NCI's clinical trials information, go to: http://cancer.gov/clinicaltrials/
- For NCI's general information about cancer, go to: http://cancer.gov/cancerinfo/

SWOG Authorized Document

- For the National Center for Complementary and Alternative Medicine (NCCAM), go to: http://nccam.nih.gov/.

- For the Office of Research on Women's Health (ORWH), go to http://orwh.od.nih.gov/.

  You will get a copy of this form. If you want more information about this study, ask your study doctor.

Future Contact

Occasionally, researchers working with SWOG may have another research idea that relates to people who were on a SWOG study. In some cases, to carry out the new research, we would need to contact patients in a particular study. You can agree or not agree to future contact by circling “yes” or “no”.

I agree to allow my study doctor, or someone approved by my study doctor, to contact me regarding future research involving my participation in this study.

Yes **No**

Consent Form for Use of Specimens for Research

We would like to keep some of the blood and urine specimens that are left over for future research. If you agree, these specimens will be kept and may be used in research to learn more about cancer and other diseases. Please read the information sheet called "How Are Specimens Used for Research" to learn more about tissue research.

The research that may be done with your specimens are not designed specifically to help you. It might help people who have cancer and other diseases in the future.

Reports about research done with your specimens will not be given to you or your doctor. These reports will not be put in your health record. The research will not have an effect on your care.

Things to Think About

The choice to let us keep the left over specimens for future research is up to you. No matter what you decide to do, it will not affect your care.

If you decide now that your specimens can be kept for research, you can change your mind at any time. Just contact us and let us know that you do not want us to use your specimens. Then any specimens that remain will no longer be used for research.

In the future, people who do research may need to know more about your health. While SWOG may give them reports about your health, it will not give them your name, address, phone number, or any other information that will let the researchers know who you are.

SWOG Authorized Document

Sometimes specimens are used for genetic research (about diseases that are passed on in families). Even if your specimens are used for this kind of research, the results will not be put in your health records.

If your confidential genetic information is discovered, you may suffer from genetic discrimination. Genetic discrimination occurs if people are treated unfairly because of differences in their genes that increase their chances of getting a certain disease. In the past, this could have resulted in the loss of health insurance or employment. Because of this, The Genetic Information Nondiscrimination Act of 2008, also referred to as GINA, was passed by Congress to protect Americans from such discrimination. The new law prevents discrimination from health insurers and employers. This act was signed into federal law on May 21, 2008, and went into effect May 2009. This law does not cover life insurance, disability insurance and long-term care insurance.

While this study has safeguards in place to protect your confidential genetic information and to make it extremely unlikely that your identity would be connected with any special studies that are performed on your tissue, it is possible that this information could be discovered by someone who is unauthorized to have access to it.

Your specimens will be used only for research and will not be sold. The research done with your specimens may help to develop new products in the future.

Benefits

The benefits of research using specimens include learning more about what causes cancer and other diseases, how to prevent them, and how to treat them.

Risks

The greatest risk to you is the release of information from your health records. We will do our best to make sure that your personal information will be kept private. The chance that this information will be given to someone else is very small.

Making Your Choice

Please read each sentence below and think about your choice. After reading each sentence, circle "Yes" or "No." If you have any questions, please talk to your doctor or nurse, or call our research review board at IRB's phone number.

No matter what you decide to do, it will not affect your care.

SWOG Authorized Document

Future Use of Specimens

1. **My specimens may be kept for use in research to learn about, prevent, treat or cure cancer.**

   Yes **No**

2. **My specimens may be kept for use in research about other health problems (for example: diabetes, Alzheimer's disease, or heart disease).**

   Yes **No**

3. **Someone may contact me in the future to ask me to allow other uses of my specimens.**

   Yes **No**

If you decide to withdraw your specimens from the SWOG Repository in the future, a written withdrawal of consent should be submitted by your study doctor to the SWOG Operations Office. Please designate in the written withdrawal whether you would prefer to have the specimens destroyed or returned to the study doctor.

Signature

I have been given a copy of all _____ *[insert total of number of pages]* pages of this form. I have read it or it has been read to me. I understand the information and have had my questions answered. I agree to take part in this study.

Patient ____

Date ____

SWOG Authorized Document

Specimen Consent Supplemental Sheets

How are Specimens Used for Research?

Where do specimens come from?

A specimen may be from a blood sample or from bone marrow, tissue from a biopsy, skin, toenails or other body materials. People who are trained to handle specimens and protect donors' rights make sure that the highest standards of quality control are followed by SWOG. Your doctor does not work for SWOG, but has agreed to help collect specimens from many patients. Many doctors across the country are helping in the same way.

Why do people do research with specimens?

Research with specimens can help to find out more about what causes cancer, how to prevent it, how to treat it, and how to cure it. Research using specimens can also answer other health questions. Some of these include finding the causes of diabetes and heart disease, or finding genetic links to Alzheimer's.

What type of research will be done with my specimen?

Many different kinds of studies use specimens. Some researchers may develop new tests to find diseases. Others may develop new ways to treat or even cure diseases. In the future, some of the research may help to develop new products, such as tests and drugs. Some research looks at diseases that are passed on in families (called genetic research). Research done with your specimen may look for genetic causes and signs of disease.

How do researchers get the specimen?

Researchers from universities, hospitals, and other health organizations conduct research using specimens. They contact SWOG and request samples for their studies. SWOG reviews the way that these studies will be done, and decides if any of the samples can be used. SWOG gets the specimen and information about you from your hospital, and sends the specimen samples and some information about you to the researcher. SWOG will not send your name, address, phone number, social security number or any other identifying information to the researcher.

Will I find out the results of the research using my specimen?

You will not receive the results of research done with your specimen. This is because research can take a long time and must use specimen samples from many people before results are known. Results from research using your specimen may not be ready for many years and will not affect your care right now, but they may be helpful to people like you in the future.

Why do you need information from my health records?

In order to do research with your specimen, researchers may need to know some things about you. (For example: Are you male or female? What is your race or ethnic group? How old are you? Have you ever smoked?) This helps researchers answer questions about diseases. The information that will be given to the researcher may include your age, sex, race, diagnosis, treatments and family history. This information is collected by your hospital from your health record and sent to SWOG. If more information is needed, SWOG will send it to the researcher.

Will my name be attached to the records that are given to the researcher?

No. Your name, address, phone number and anything else that could identify you will be removed before they go the researcher. The researcher will not know who you are.

SWOG Authorized Document

How could the records be used in ways that might be harmful to me?

Sometimes, health records have been used against patients and their families. For example, insurance companies may deny a patient insurance or employers may not hire someone with a certain illness (such as AIDS or cancer). The results of genetic research may not apply only to you, but to your family members too. For disease caused by gene changes, the information in one person's health record could be used against family members.

How am I protected?

SWOG is in charge of making sure that information about you is kept private. SWOG will take careful steps to prevent misuse of records. Your name, address, phone number and any other identifying information will be taken off anything associated with your specimen before it is given to the researcher. This would make it very difficult for any research results to be linked to you or your family. Also, people outside the research process will not have access to results about any one person which will help to protect your privacy.

What if I have more questions?

If you have any questions, please talk to your doctor or nurse, or call our research review board at (Insert IRB's Phone Number).

SWOG Authorized Document

DRAFT

SWOG
S1200 REGISTRATION WORKSHEET

RANDOMIZED BLINDED SHAM- AND WAITLIST-CONTROLLED TRIAL OF ACUPUNCTURE FOR JOINT SYMPTOMS RELATED TO AROMATASE INHIBITORS IN WOMEN WITH EARLY STAGE BREAST CANCER

Activation Date

Last Amended Date

Registration Step 1

INSTRUCTIONS: All of the information on this Registration Worksheet and the Protocol Eligibility Section must be answered appropriately for a patient to be considered eligible for registration. This Registration Worksheet must be entirely filled out and referred to during the registration. **Do NOT submit this worksheet as part of the patient data.**

SWOG PATIENT ID

If the patient has a SWOG Patient ID assigned by a prior registration or Specimen Tracking, choose "Previous Patient" and use that number.

SWOG Patient ID Status: ☐ New Patient ☐ Previous Patient: **SWOG Patient ID:** ☐☐☐☐☐☐

DEMOGRAPHY

Full names preferred, initials OK

Patient First Name: ____________________

Patient Middle Name: ____________________

Patient Last Name: ____________________

FOR SWOG INSTITUTIONS

Registrar's SWOG Roster ID Number: ☐☐☐☐☐

SWOG Investigator Number: ☐☐☐☐☐

SWOG Treating Institution Number: ☐☐☐☐☐

PATIENT INFORMATION

Date Informed Consent Signed: ☐☐ / ☐☐ / ☐☐☐☐

Date HIPAA Authorization signed: ☐☐ / ☐☐ / ☐☐☐☐ *(Not required if Country of Residence is not USA)*

Projected Start Date of Treatment: ☐☐ / ☐☐ / ☐☐☐☐

Stratification Question

Study Site: ☐ (1) Columbia U ☐ (2) FHCRC ☐ (3) Grand Rapids CCOP ☐ (4) Kaiser Permanente NoCa ☐ (5) Oregon HSU ☐ (6) USC

Indicate how the patient answered the following questions on the consent form

**My specimens may be kept for use in research to learn about, prevent, treat, or cure cancer.**	☐ Yes	☐ No
**My specimens may be kept for use in research about other health problems (for example: diabetes, Alzheimer's disease, or heart disease).**	☐ Yes	☐ No
**Someone may contact me in the future to ask me to allow other uses of my specimens.**	☐ Yes	☐ No
**I agree to allow my study doctor, or someone approved by my study doctor, to contact me regarding future research involving my participation in this study.**	☐ Yes	☐ No

Patient Eligibility

**Has the SWOG Registration Worksheet been completed entirely and is the patient eligible according to the current version of protocol section 5.0?**	☐ Yes	☐ No

DRAFT

SWOG
S1200 PRESTUDY FORM

SWOG Patient ID ☐☐☐☐☐☐ **SWOG Study No.** S 1 2 0 0 **Registration Step** 1

Patient Initials ____________ (L, F M)

Institution / Affiliate ________________________________ Physician ________________________________

Instructions: Submit this form within 7 days of registration. All dates are **MONTH, DAY, YEAR**. Explain any blank fields or blank dates in the **Comments** section. Place an X in appropriate boxes. Circle **AMENDED** items in red and write **AMENDED** across top of form.

ELIGIBILITY VERIFICATION:

Each of the fields below corresponds to a criterion in Section 5 and must be completed for patient to be eligible.

PATIENT AND DISEASE DESCRIPTION

Performance Status: ☐ 0 ☐ 1 **Height:** ☐☐☐ cm **Weight:** ☐☐☐.☐ kg

Date of initial diagnosis of breast cancer: ☐☐ / ☐☐ / ☐☐☐☐

Stage of breast cancer (select one): ☐ I ☐ II ☐ III

Tumor Laterality (select one): ☐ Left ☐ Right ☐ Bilateral

Prior surgery (select one):

☐ Modified radical mastectomy, date: ☐☐ / ☐☐ / ☐☐☐☐

☐ Breast sparing surgery, date: ☐☐ / ☐☐ / ☐☐☐☐

RECEPTOR STATUS

(Use institutional standards; borderline results should be reported as positive.)

ER Status: ☐ ER- ☐ ER+

PgR Status: ☐ PgR- ☐ PgR+

HER-2/neu status: ☐ HER2+ ☐ HER2- ☐ Equivocal ☐ Not done

Spontaneous menstrual bleeding within the last 12 months? ☐ Yes ☐ No

Month and year of last menstrual period: ☐☐ / ☐☐☐☐

Current GnRH agonist? ☐ Yes ☐ No

Date started: ☐☐ / ☐☐ / ☐☐☐☐

Hysterectomy? ☐ Yes ☐ No

Bilateral oophorectomy? ☐ Yes ☐ No ☐ Unknown

Serum FSH: ☐☐☐.☐☐ IU/L **Date FSH obtained:** ☐☐ / ☐☐ / ☐☐☐☐

Institutional post-menopausal range: ☐☐.☐☐ IU/L - ☐☐☐.☐☐ IU/L

continued on next page

SWOG Patient ID ☐☐☐☐☐☐ **SWOG Study No.** S 1 2 0 0 **Registration Step** 1

Patient Initials__________ (L, F M)

ADDITIONAL PRESTUDY DATA:

Has the patient ever had acupuncture treatment? ☐ Yes ☐ No

If Yes, reason: ____________________

Date of last treatment: ☐☐ / ☐☐ / ☐☐☐☐

Does the patient have a severe bleeding disorder? ☐ Yes ☐ No

Is the patient right- or left-handed? (select one) ☐ Right-handed ☐ Left-handed ☐ Ambidextrous

Has the patient's joint pain/stiffness recorded on the Brief Pain Inventory (select one):

- ☐ started after initiating aromatase inhibitor therapy
- ☐ worsened after initiating aromatase inhibitor therapy

PRIOR TREATMENT RELATED TO THIS CANCER

Prior Therapy Type(s) *(select all that apply)***:**

☐ Aromatase Inhibitor Date started: ☐☐ / ☐☐ / ☐☐☐☐

Type of Aromatase Inhibitor:

- ☐ Anastrozole/Arimadex
- ☐ Letrozole/Femara
- ☐ Exemestane/Aromasin

☐ Bisphosphonate Date started: ☐☐ / ☐☐ / ☐☐☐☐

☐ Tamoxifen/Nolvadex

☐ Chemotherapy

☐ Radiation therapy

Has the patient received prior taxane therapy for any reason? ☐ Yes ☐ No

Comorbid Conditions

- ☐ Renal insufficiency
- ☐ Abnormal pulmonary function
- ☐ Hypertension
- ☐ Heart disease
- ☐ Hearing impairment
- ☐ Visual impairment
- ☐ Deformity or orthopedic impairment
- ☐ Diminished mental capacity/neurologic impairment/dementia
- ☐ Arthritis
- ☐ HIV/AIDS
- ☐ Other comorbidities (please specify): ____________________

Comments:

DRAFT

SWOG
S1200 COVER SHEET FOR PATIENT-COMPLETED QUESTIONNAIRES

SWOG Patient ID ☐☐☐☐☐☐ **SWOG Study No.** S 1 2 0 0 **Registration Step** 1

Patient Initials ____________ (L, F M) **Date Form Completed:** ☐☐ / ☐☐ / ☐☐☐☐

Institution / Affiliate ________________________ Physician ________________________________

Planned Assessment:

☐ Baseline ☐ 6-week visit ☐ 12-week visit ☐ 16-week contact ☐ 20-week contact

☐ 24-week visit ☐ 52-week visit

Instructions: Please complete form at time of registration and at 6 weeks, 12 weeks, 16 weeks, 20 weeks, 24 weeks, and 52 weeks post-registration. **Note: The prestudy baseline Brief Pain Inventory Short Form is an eligibility requirement and must be completed within 14 days prior to registration. All other baseline forms must be completed prior to initiation of acupuncture treatment.** All dates are **MONTH, DAY, YEAR**. Place an ☒ in appropriate boxes. Circle **AMENDED** items in red and write **AMENDED** across the top of this form or any of the attached patient-completed forms.

Was the Brief Pain Inventory Short Form (BPI-SF) completed? ☐ No ☐ Yes

Was the WOMAC Index completed? ☐ No ☐ Yes

Was the M-SACRAH completed? ☐ No ☐ Yes

Was the PROMIS PI-SF completed? ☐ No ☐ Yes

Was the FACT-ES Trial Outcome Index completed? ☐ No ☐ Yes

Was the Aromatase Inhibitor Usage Form completed? ☐ No ☐ Yes

Was the S1200 Assessment of Blinding Form completed? ☐ No ☐ Yes

If any of the questionnaires was completed:

Did the patient require any assistance in completing the questionnaire(s)? ☐ No ☐ Yes

If Yes, Describe: ____

Method of completing questionnaire(s):

☐ In the clinic

☐ By telephone

☐ Other (Please specify): ____

If not completed, please give reason (select one):

☐ Illness/deteriorating health

☐ Not illness related (e.g., unable to contact, patient refusal, patient failure to return questionnaire)

☐ Institution error (e.g., forgot to administer, did not continue schedule when patient went off treatment)

☐ Death

☐ Other

____

I have reviewed the Cover Sheet and Questionnaire(s). All forms are complete or an explanation is given for any missing data.

Person completing form, last name: ______________________________ Person completing form, phone: (☐☐☐) ☐☐☐ - ☐☐☐☐

Comments:

11/29/2011

Draft

DRAFT

SWOG
S1200 BRIEF PAIN INVENTORY SHORT FORM (BPI-SF)

SWOG Patient ID ☐☐☐☐☐☐ **SWOG Study No.** S 1 2 0 0 **Registration Step** 1

Patient Initials ____________ (L, F M) **Date of Completion:** ☐☐ / ☐☐ / ☐☐☐☐

Planned Assessment: ☐ Prestudy ☐ 6-week visit ☐ 12-week visit ☐ 16-week contact ☐ 20-week contact ☐ 24-week visit ☐ 52-week visit

Institution/Affiliate ______________________________ Physician ______________________________

Instructions: You will be asked to fill out this form at the times listed above for a total of 7 times. The staff at your clinic will remind you of the times the questionnaire is to be completed. For each item below, please put an X in the one box that best describes your joint pain (page 1) and how that pain affects your daily activites (page 2). Be sure to read each question carefully and to provide one answer for each question. Thank you for participating in this study.

1. **Throughout our lives, most of us have had pain from time to time (such as minor headaches, sprains, and toothaches). Have you had joint pain other than these everyday kinds of pain in the last week?**

   ☐ Yes ☐ No

2. **Please rate your joint pain by selecting the one number that best describes your joint pain at its WORST in the last week.**

   ☐ 0 ☐ 1 ☐ 2 ☐ 3 ☐ 4 ☐ 5 ☐ 6 ☐ 7 ☐ 8 ☐ 9 ☐ 10
   No pain — Pain as bad as you can imagine

3. **Please rate your joint pain by selecting the one number that best describes your joint pain at its LEAST in the last week.**

   ☐ 0 ☐ 1 ☐ 2 ☐ 3 ☐ 4 ☐ 5 ☐ 6 ☐ 7 ☐ 8 ☐ 9 ☐ 10
   No pain — Pain as bad as you can imagine

4. **Please rate your joint pain by selecting the one number that best describes your joint pain on the AVERAGE.**

   ☐ 0 ☐ 1 ☐ 2 ☐ 3 ☐ 4 ☐ 5 ☐ 6 ☐ 7 ☐ 8 ☐ 9 ☐ 10
   No pain — Pain as bad as you can imagine

5. **Please rate your joint pain by selecting the one number that tells how much joint pain you have RIGHT NOW.**

   ☐ 0 ☐ 1 ☐ 2 ☐ 3 ☐ 4 ☐ 5 ☐ 6 ☐ 7 ☐ 8 ☐ 9 ☐ 10
   No pain — Pain as bad as you can imagine

6. **Are you taking any oral medications for your joint pain?** ☐ Yes ☐ No

7. **In the last week, how much RELIEF have joint pain treatments or medications provided? Please select the percentage that most shows how much.**

   ☐ 0% ☐ 10% ☐ 20% ☐ 30% ☐ 40% ☐ 50% ☐ 60% ☐ 70% ☐ 80% ☐ 90% ☐ 100%
   No relief — Complete relief

continued on next page

SWOG
S1200 BRIEF PAIN INVENTORY SHORT FORM (BPI-SF)

SWOG Patient ID ☐☐☐☐☐☐ **SWOG Study No.** S 1 2 0 0 **Registration Step** 1

Patient Initials ____________ (L, F M) **Date of Completion:** ☐☐ / ☐☐ / ☐☐☐☐

Planned Assessment: ☐ Prestudy ☐ 6-week visit ☐ 12-week visit ☐ 16-week contact ☐ 20-week contact ☐ 24-week visit ☐ 52-week visit

...Continued from Page 1

Select the one number that describes how, during the last week, JOINT PAIN HAS INTERFERED with your:

8. General Activity

☐ 0 ☐ 1 ☐ 2 ☐ 3 ☐ 4 ☐ 5 ☐ 6 ☐ 7 ☐ 8 ☐ 9 ☐ 10

Does not interfere — Completely interferes

9. Mood

☐ 0 ☐ 1 ☐ 2 ☐ 3 ☐ 4 ☐ 5 ☐ 6 ☐ 7 ☐ 8 ☐ 9 ☐ 10

Does not interfere — Completely interferes

10. Walking ability

☐ 0 ☐ 1 ☐ 2 ☐ 3 ☐ 4 ☐ 5 ☐ 6 ☐ 7 ☐ 8 ☐ 9 ☐ 10

Does not interfere — Completely interferes

11. Normal work (includes both work outside the home and housework)

☐ 0 ☐ 1 ☐ 2 ☐ 3 ☐ 4 ☐ 5 ☐ 6 ☐ 7 ☐ 8 ☐ 9 ☐ 10

Does not interfere — Completely interferes

12. Relations with other people

☐ 0 ☐ 1 ☐ 2 ☐ 3 ☐ 4 ☐ 5 ☐ 6 ☐ 7 ☐ 8 ☐ 9 ☐ 10

Does not interfere — Completely interferes

13. Sleep

☐ 0 ☐ 1 ☐ 2 ☐ 3 ☐ 4 ☐ 5 ☐ 6 ☐ 7 ☐ 8 ☐ 9 ☐ 10

Does not interfere — Completely interferes

14. Enjoyment of life

☐ 0 ☐ 1 ☐ 2 ☐ 3 ☐ 4 ☐ 5 ☐ 6 ☐ 7 ☐ 8 ☐ 9 ☐ 10

Does not interfere — Completely interferes

Source: Brief Pain Inventory-Short Form: C. S. Cleeland, PhD, University of Texas, M. D. Anderson Cancer Center, Houston, TX (Cleeland, 1994)

continued on next page

DRAFT

SWOG
S1200 BRIEF PAIN INVENTORY SHORT FORM (BPI-SF)

SWOG Patient ID ☐☐☐☐☐☐ **SWOG Study No.** S1200 **Registration Step** 1

Patient Initials ____________ (L, F M) **Date of Completion:** ☐☐ / ☐☐ / ☐☐☐☐

Planned Assessment: ☐ Prestudy ☐ 6-week visit ☐ 12-week visit ☐ 16-week contact ☐ 20-week contact ☐ 24-week visit ☐ 52-week visit

Instructions: In the first two questions on page 1 of this form, you were asked about joint pain. The two questions below ask instead about joint stiffness. For each item below, please put an ☒ in the one box that best describes your joint stiffness. Be sure to read each question carefully and to provide one answer.

15. Throughout our lives, most of us have had stiffness from time to time (such as difficulty getting up from a chair). Have you had joint stiffness in the last week?

☐ Yes ☐ No

16. Please rate your joint stiffness by selecting the one number that best describes your joint stiffness at its WORST in the last week.

☐ 0 ☐ 1 ☐ 2 ☐ 3 ☐ 4 ☐ 5 ☐ 6 ☐ 7 ☐ 8 ☐ 9 ☐ 10

No stiffness — Stiffness as bad as you can imagine

DRAFT

SWOG
S1200 WESTERN ONTARIO AND MCMASTER UNIVERSITIES OSTEOARTHRITIS (WOMAC) INDEX (VERSION 3.1)

SWOG Patient ID ☐☐☐☐☐☐ **SWOG Study No.** S 1 2 0 0 **Registration Step** 1

Patient Initials ____________ (L, F M) **Date of Completion:** ☐☐ / ☐☐ / ☐☐☐☐

Planned Assessment: ☐ Baseline ☐ 6-week visit ☐ 12-week visit ☐ 24-week visit ☐ 52-week visit

Institution/Affiliate ________________________________ Physician ______________________________

Instructions: You will be asked to complete this form at time of registration and at 6 weeks, 12 weeks, 24 weeks, and 52 weeks post-registration. All dates are **MONTH, DAY, YEAR**. Thank you for your participation on this study.

Section A: **PAIN**

Think about the pain you felt during the past week caused by your joints. For each of the following questions, select the number between "0" and "10" that best describes the amount of **pain** you have experienced by putting an X in the box next to that number.

Question: **How much pain have you had...**

1. **when walking on a flat surface?**

☐ 0 ☐ 1 ☐ 2 ☐ 3 ☐ 4 ☐ 5 ☐ 6 ☐ 7 ☐ 8 ☐ 9 ☐ 10
No Pain — Extreme Pain

2. **when going up or down stairs?**

☐ 0 ☐ 1 ☐ 2 ☐ 3 ☐ 4 ☐ 5 ☐ 6 ☐ 7 ☐ 8 ☐ 9 ☐ 10
No Pain — Extreme Pain

3. **at night while in bed? (that is - pain that disturbs your sleep)**

☐ 0 ☐ 1 ☐ 2 ☐ 3 ☐ 4 ☐ 5 ☐ 6 ☐ 7 ☐ 8 ☐ 9 ☐ 10
No Pain — Extreme Pain

4. **while sitting or lying down?**

☐ 0 ☐ 1 ☐ 2 ☐ 3 ☐ 4 ☐ 5 ☐ 6 ☐ 7 ☐ 8 ☐ 9 ☐ 10
No Pain — Extreme Pain

5. **while standing?**

☐ 0 ☐ 1 ☐ 2 ☐ 3 ☐ 4 ☐ 5 ☐ 6 ☐ 7 ☐ 8 ☐ 9 ☐ 10
No Pain — Extreme Pain

Section B: **STIFFNESS**

Think about the stiffness (not pain) you felt during the past week caused by your joints. Stiffness is a sensation of **decreased** ease in moving your joint. For each of the following questions, select the number between "0" and "10" that best describes the amount of **stiffness** you have experienced by putting an X in the box next to that number.

6. **How severe has your stiffness been after you first woke up in the morning?**

☐ 0 ☐ 1 ☐ 2 ☐ 3 ☐ 4 ☐ 5 ☐ 6 ☐ 7 ☐ 8 ☐ 9 ☐ 10
No Stiffness — Extreme Stiffness

7. **How severe has your stiffness been after sitting or lying down while resting later in the day?**

☐ 0 ☐ 1 ☐ 2 ☐ 3 ☐ 4 ☐ 5 ☐ 6 ☐ 7 ☐ 8 ☐ 9 ☐ 10
No Stiffness — Extreme Stiffness

continued on next page

DRAFT

SWOG
S1200 WESTERN ONTARIO AND MCMASTER UNIVERSITIES OSTEOARTHRITIS (WOMAC) INDEX (VERSION 3.1)

SWOG Patient ID ☐☐☐☐☐☐ **SWOG Study No.** S 1 2 0 0 **Registration Step** 1

Patient Initials ____________ (L, F M) **Date of Completion:** ☐☐ / ☐☐ / ☐☐☐☐

Planned Assessment: ☐ Baseline ☐ 6-week visit ☐ 12-week visit ☐ 24-week visit ☐ 52-week visit

...Continued from Page 1

Section C: DIFFICULTY PERFORMING DAILY ACTIVITIES

Think about the difficulty you had doing the following daily physical activities during the past week caused by your joints. By this we mean **your ability to move around and take care of yourself**. For each of the following questions, select the number between "0" and "10" that best describes the amount of **difficulty** you have experienced by putting an X in the box next to that number.

Question: **How much difficulty have you had...**

8. when going down the stairs?
☐ 0 ☐ 1 ☐ 2 ☐ 3 ☐ 4 ☐ 5 ☐ 6 ☐ 7 ☐ 8 ☐ 9 ☐ 10
No Difficulty — Extreme Difficulty

9. when going up the stairs?
☐ 0 ☐ 1 ☐ 2 ☐ 3 ☐ 4 ☐ 5 ☐ 6 ☐ 7 ☐ 8 ☐ 9 ☐ 10
No Difficulty — Extreme Difficulty

10. when getting up from a sitting position?
☐ 0 ☐ 1 ☐ 2 ☐ 3 ☐ 4 ☐ 5 ☐ 6 ☐ 7 ☐ 8 ☐ 9 ☐ 10
No Difficulty — Extreme Difficulty

11. while standing?
☐ 0 ☐ 1 ☐ 2 ☐ 3 ☐ 4 ☐ 5 ☐ 6 ☐ 7 ☐ 8 ☐ 9 ☐ 10
No Difficulty — Extreme Difficulty

12. when bending to the floor?
☐ 0 ☐ 1 ☐ 2 ☐ 3 ☐ 4 ☐ 5 ☐ 6 ☐ 7 ☐ 8 ☐ 9 ☐ 10
No Difficulty — Extreme Difficulty

13. when walking on a flat surface?
☐ 0 ☐ 1 ☐ 2 ☐ 3 ☐ 4 ☐ 5 ☐ 6 ☐ 7 ☐ 8 ☐ 9 ☐ 10
No Difficulty — Extreme Difficulty

14. getting in or out of a car, or getting on or off a bus?
☐ 0 ☐ 1 ☐ 2 ☐ 3 ☐ 4 ☐ 5 ☐ 6 ☐ 7 ☐ 8 ☐ 9 ☐ 10
No Difficulty — Extreme Difficulty

15. while going shopping?
☐ 0 ☐ 1 ☐ 2 ☐ 3 ☐ 4 ☐ 5 ☐ 6 ☐ 7 ☐ 8 ☐ 9 ☐ 10
No Difficulty — Extreme Difficulty

16. when putting on your socks or panty hose or stockings?
☐ 0 ☐ 1 ☐ 2 ☐ 3 ☐ 4 ☐ 5 ☐ 6 ☐ 7 ☐ 8 ☐ 9 ☐ 10
No Difficulty — Extreme Difficulty

continued on next page

SWOG
S1200 WESTERN ONTARIO AND MCMASTER UNIVERSITIES OSTEOARTHRITIS (WOMAC) INDEX (VERSION 3.1)

SWOG Patient ID ☐☐☐☐☐☐ **SWOG Study No.** S 1 2 0 0 **Registration Step** 1

Patient Initials ____________ (L, F M) **Date of Completion:** ☐☐ / ☐☐ / ☐☐☐☐

Planned Assessment: ☐ Baseline ☐ 6-week visit ☐ 12-week visit ☐ 24-week visit ☐ 52-week visit

...Continued from Page 2

17. when getting out of bed?

☐ 0 ☐ 1 ☐ 2 ☐ 3 ☐ 4 ☐ 5 ☐ 6 ☐ 7 ☐ 8 ☐ 9 ☐ 10

No Difficulty — Extreme Difficulty

18. when taking off your socks or panty hose or stockings?

☐ 0 ☐ 1 ☐ 2 ☐ 3 ☐ 4 ☐ 5 ☐ 6 ☐ 7 ☐ 8 ☐ 9 ☐ 10

No Difficulty — Extreme Difficulty

19. while lying in bed?

☐ 0 ☐ 1 ☐ 2 ☐ 3 ☐ 4 ☐ 5 ☐ 6 ☐ 7 ☐ 8 ☐ 9 ☐ 10

No Difficulty — Extreme Difficulty

20. when getting in or out of the bathtub?

☐ 0 ☐ 1 ☐ 2 ☐ 3 ☐ 4 ☐ 5 ☐ 6 ☐ 7 ☐ 8 ☐ 9 ☐ 10

No Difficulty — Extreme Difficulty

21. while sitting?

☐ 0 ☐ 1 ☐ 2 ☐ 3 ☐ 4 ☐ 5 ☐ 6 ☐ 7 ☐ 8 ☐ 9 ☐ 10

No Difficulty — Extreme Difficulty

22. when getting on or off the toilet?

☐ 0 ☐ 1 ☐ 2 ☐ 3 ☐ 4 ☐ 5 ☐ 6 ☐ 7 ☐ 8 ☐ 9 ☐ 10

No Difficulty — Extreme Difficulty

23. while doing heavy household chores?

☐ 0 ☐ 1 ☐ 2 ☐ 3 ☐ 4 ☐ 5 ☐ 6 ☐ 7 ☐ 8 ☐ 9 ☐ 10

No Difficulty — Extreme Difficulty

24. while doing light household chores?

☐ 0 ☐ 1 ☐ 2 ☐ 3 ☐ 4 ☐ 5 ☐ 6 ☐ 7 ☐ 8 ☐ 9 ☐ 10

No Difficulty — Extreme Difficulty

Bellamy N. WOMAC: The WOMAC Knee and Hip Osteoarthritis Indices: Development, validation, globalization and influence on the development of the AUSCAN Hand Osteoarthritis Indices. Clin Exp Rheumatol 2005;23:(Suppl 39): 148-153./Bellamy N. WOMAC Osteoarthritis Index: User Guide. Queensland: CONROD, The University of Queensland; 2002./Theiler R, Spielberger J, Bischoff HA, et al. Clinical evaluation of the WOMAC 3.0 OA Index in numeric rating scale format using a computerized touch screen version. OsteoArthritis and Cartilage. 2002;10:479-481.

DRAFT

SWOG
S1200 MODIFIED SCORE FOR THE ASSESSMENT AND QUANTIFICATION OF CHRONIC RHEUMATOID AFFECTIONS OF THE HANDS (M-SACRAH)

SWOG Patient ID ☐☐☐☐☐☐ **SWOG Study No.** S 1 2 0 0 **Registration Step** 1

Patient Initials ____________ (L, F M) **Date of Completion:** ☐☐ / ☐☐ / ☐☐☐☐

Planned Assessment: ☐ Baseline ☐ 6-week visit ☐ 12-week visit ☐ 24-week visit ☐ 52-week visit

Institution/Affiliate ________________________________ Physician ________________________________

Instructions: You will be asked to complete this form at time of registration and at 6 weeks, 12 weeks, 24 weeks , and 52 weeks post-registration. All dates are **MONTH, DAY, YEAR**. Thank you for your participation on this study.

DIFFICULTIES IN COPING WITH ACTIVITIES OF DAILY LIFE

Please think of the difficulties you had during the past week in performing the following activities of daily life in view of your **finger joint conditions**. The objective is to **move freely** and to be able to **take care of yourself**. For each question below, please put an ☒ in the box that describes how difficult each activity was for you.

QUESTION: How difficult was it for you to:

1. **Unlock your door with a key?**

☐ 0 ☐ 1 ☐ 2 ☐ 3 ☐ 4 ☐ 5 ☐ 6 ☐ 7 ☐ 8 ☐ 9 ☐ 10
Not Difficult — Extremely Difficult

2. **Button and unbutton your shirt/blouse?**

☐ 0 ☐ 1 ☐ 2 ☐ 3 ☐ 4 ☐ 5 ☐ 6 ☐ 7 ☐ 8 ☐ 9 ☐ 10
Not Difficult — Extremely Difficult

3. **Turn a faucet?**

☐ 0 ☐ 1 ☐ 2 ☐ 3 ☐ 4 ☐ 5 ☐ 6 ☐ 7 ☐ 8 ☐ 9 ☐ 10
Not Difficult — Extremely Difficult

4. **Do up or undo a zipper?**

☐ 0 ☐ 1 ☐ 2 ☐ 3 ☐ 4 ☐ 5 ☐ 6 ☐ 7 ☐ 8 ☐ 9 ☐ 10
Not Difficult — Extremely Difficult

5. **Tie your shoelaces?**

☐ 0 ☐ 1 ☐ 2 ☐ 3 ☐ 4 ☐ 5 ☐ 6 ☐ 7 ☐ 8 ☐ 9 ☐ 10
Not Difficult — Extremely Difficult

6. **Unscrew the toothpaste cap?**

☐ 0 ☐ 1 ☐ 2 ☐ 3 ☐ 4 ☐ 5 ☐ 6 ☐ 7 ☐ 8 ☐ 9 ☐ 10
Not Difficult — Extremely Difficult

7. **Turn the pages of the newspaper?**

☐ 0 ☐ 1 ☐ 2 ☐ 3 ☐ 4 ☐ 5 ☐ 6 ☐ 7 ☐ 8 ☐ 9 ☐ 10
Not Difficult — Extremely Difficult

8. **Write?**

☐ 0 ☐ 1 ☐ 2 ☐ 3 ☐ 4 ☐ 5 ☐ 6 ☐ 7 ☐ 8 ☐ 9 ☐ 10
Not Difficult — Extremely Difficult

continued on next page

DRAFT

SWOG
S1200 MODIFIED SCORE FOR THE ASSESSMENT AND QUANTIFICATION OF CHRONIC RHEUMATOID AFFECTIONS OF THE HANDS (M-SACRAH)

SWOG Patient ID ☐☐☐☐☐☐ **SWOG Study No.** S 1 2 0 0 **Registration Step** 1

Patient Initials ____________ (L, F M) **Date of Completion:** ☐☐ / ☐☐ / ☐☐☐☐

Planned Assessment: ☐ Baseline ☐ 6-week visit ☐ 12-week visit ☐ 24-week visit ☐ 52-week visit

...Continued from Page 1

STIFFNESS

Please, assess the **stiffness** (not the pain) you had during the past week caused by your **finger joint condition**. Stiffness is normally defined by a **reduction in the movability of joints**. For each question below, please put an ☒ in the box that describes the severity of your joint stiffness.

9. How severe was your joint stiffness immediately after waking up first thing in the morning?

☐ 0 ☐ 1 ☐ 2 ☐ 3 ☐ 4 ☐ 5 ☐ 6 ☐ 7 ☐ 8 ☐ 9 ☐ 10

Not Stiff — Unbearably Stiff

10. How severe was your joint stiffness later in the day, after a period of resting time?

☐ 0 ☐ 1 ☐ 2 ☐ 3 ☐ 4 ☐ 5 ☐ 6 ☐ 7 ☐ 8 ☐ 9 ☐ 10

Not Stiff — Unbearably Stiff

PAIN

Please, assess the **pain** you had during the past week caused by your **finger joint condition**. For each question below, please put an ☒ in the box that describes the severity of your joint pain.

QUESTION: How severe was your pain:

11. During intensive work?

☐ 0 ☐ 1 ☐ 2 ☐ 3 ☐ 4 ☐ 5 ☐ 6 ☐ 7 ☐ 8 ☐ 9 ☐ 10

No Pain — Unbearable Pain

12. At times of inactivity?

☐ 0 ☐ 1 ☐ 2 ☐ 3 ☐ 4 ☐ 5 ☐ 6 ☐ 7 ☐ 8 ☐ 9 ☐ 10

No Pain — Unbearable Pain

Sautner J, Andel I, Rintelen B, Leeb BF: M-SACRAH. A modified, shortened version of SACRAH (Score for the Assessment and Quantification of Chronic Rheumatoid Affections of the Hands); Rheumatology (Oxford). 2004 Nov;43(11):1409-13.

DRAFT

SWOG
S1200 PROMIS PAIN IMPACT - SHORT FORM (PROMIS PI-SF)

SWOG Patient ID ☐☐☐☐☐☐ **SWOG Study No.** S 1 2 0 0 **Registration Step** 1

Patient Initials ____________ (L, F M) **Date of Completion:** ☐☐ / ☐☐ / ☐☐☐☐

Planned Assessment: ☐ Baseline ☐ 6-week visit ☐ 12-week visit ☐ 24-week visit ☐ 52-week visit

Institution/Affiliate ________________________________ Physician ________________________________

Instructions: You will be asked to complete this form at time of registration and at 6 weeks, 12 weeks, 24 weeks , and 52 weeks post-registration. All dates are **MONTH, DAY, YEAR**. Thank you for your participation on this study.

By selecting one (1) box per line, please indicate your joint **pain** interference during the **past week**. Please select a box by marking an X in it.

	Not at all	A little bit	Somewhat	Quite a bit	Very much
1) How much did pain interfere with your enjoyment of life?	☐	☐	☐	☐	☐
2) How much did pain interfere with your ability to concentrate?	☐	☐	☐	☐	☐
3) How much did pain interfere with your day to day activities?	☐	☐	☐	☐	☐
4) How much did pain interfere with your enjoyment of recreational activities?	☐	☐	☐	☐	☐
5) How much did pain interfere with doing tasks away from home (*e.g.*, getting groceries, running errands)?	☐	☐	☐	☐	☐
	Never	Rarely	Sometimes	Often	Always
6) How often did pain keep you from socializing with others?	☐	☐	☐	☐	☐

(Needs a reference)

DRAFT

SWOG
S1200 FACT - ES TRIAL OUTCOME INDEX (VERSION 4)

SWOG Patient ID ☐☐☐☐☐☐ **SWOG Study No.** S 1 2 0 0 **Registration Step** 1

Patient Initials ________ (L, F M) **Date Form Completed:** ☐☐ / ☐☐ / ☐☐☐☐

Planned Assessment: ☐ Baseline ☐ 6-week visit ☐ 12-week visit ☐ 24-week visit ☐ 52-week visit

Institution / Affiliate ________ Physician ________

Instructions: You will be asked to complete this form at time of registration and at 6 weeks, 12 weeks, 24 weeks, and 52 weeks post-registration. All dates are **MONTH, DAY, YEAR**. Thank you for your participation on this study.

Below is a list of statements that other people with your illness have said are important.

By selecting one (1) box per line, please indicate how true each statement has been for you during the past 7 days. Please select a box by marking an X in it.

**PHYSICAL WELL-BEING**	**Not at all**	**A little bit**	**Some-what**	**Quite a bit**	**Very much**
1. I have a lack of energy	☐	☐	☐	☐	☐
2. I have nausea	☐	☐	☐	☐	☐
3. Because of my physical condition, I have trouble meeting the needs of my family	☐	☐	☐	☐	☐
4. I have pain	☐	☐	☐	☐	☐
5. I am bothered by side effects of treatment	☐	☐	☐	☐	☐
6. I feel ill	☐	☐	☐	☐	☐
7. I am forced to spend time in bed	☐	☐	☐	☐	☐

continued on next page

US English
Copyright 1987, 1997
3/20/03 version

SWOG
S1200 FACT - ES TRIAL OUTCOME INDEX (VERSION 4)

SWOG Patient ID ☐☐☐☐☐☐ **SWOG Study No.** S 1 2 0 0 **Registration Step** 1

Patient Initials ______________ (L, F M)

Planned Assessment: ☐ Baseline ☐ 6-week visit ☐ 12-week visit ☐ 24-week visit ☐ 52-week visit

...Continued from Page 1

By selecting one (1) box per line, please indicate how true each statement has been for you during the past 7 days. Please select a box by marking an X in it.

FUNCTIONAL WELL-BEING	Not at all	A little bit	Some-what	Quite a bit	Very much
1. I am able to work (include work at home)	☐	☐	☐	☐	☐
2. My work (include work at home) is fulfilling	☐	☐	☐	☐	☐
3. I am able to enjoy life	☐	☐	☐	☐	☐
4. I have accepted my illness	☐	☐	☐	☐	☐
5. I am sleeping well	☐	☐	☐	☐	☐
6. I am enjoying the things I usually do for fun	☐	☐	☐	☐	☐
7. I am content with the quality of my life right now	☐	☐	☐	☐	☐

continued on next page

Copyright 1987, 1997

SWOG
S1200 FACT - ES TRIAL OUTCOME INDEX (VERSION 4)

SWOG Patient ID ☐☐☐☐☐☐ **SWOG Study No.** S 1 2 0 0 **Registration Step** 1

Patient Initials ____________ (L, F M)

Planned Assessment: ☐ Baseline ☐ 6-week visit ☐ 12-week visit ☐ 24-week visit ☐ 52-week visit

...Continued from Page 2

By selecting one (1) box per line, please indicate how true each statement has been for you during the past 7 days. Please select a box by marking an X in it.

**ADDITIONAL CONCERNS**	**Not at all**	**A little bit**	**Some-what**	**Quite a bit**	**Very much**
1. I have hot flashes	☐	☐	☐	☐	☐
2. I have cold sweats	☐	☐	☐	☐	☐
3. I have night sweats	☐	☐	☐	☐	☐
4. I have vaginal discharge	☐	☐	☐	☐	☐
5. I have vaginal itching/irritation	☐	☐	☐	☐	☐
6. I have vaginal bleeding or spotting	☐	☐	☐	☐	☐
7. I have vaginal dryness	☐	☐	☐	☐	☐
8. I have pain or discomfort with intercourse	☐	☐	☐	☐	☐
9. I have lost interest in sex	☐	☐	☐	☐	☐
10. I have gained weight	☐	☐	☐	☐	☐
11. I feel lightheaded (dizzy)	☐	☐	☐	☐	☐
12. I have been vomiting	☐	☐	☐	☐	☐
13. I have diarrhea	☐	☐	☐	☐	☐
14. I get headaches	☐	☐	☐	☐	☐
15. I feel bloated	☐	☐	☐	☐	☐
16. I have breast sensitivity/tenderness	☐	☐	☐	☐	☐
17. I have mood swings	☐	☐	☐	☐	☐
18. I am irritable	☐	☐	☐	☐	☐

Thank you for completing this questionnaire.

Cella DF, Tulsky DS, Gray G, et al. The Functional Assessment of Cancer Therapy scale: development and validation of the general measure. J Clin Oncol 1993;11:570-579/Fallowfield LJ, Leaity SK, Howell A, et al. Assessment of quality of life in women undergoing hormonal therapy for breast cancer: validation of an endocrine symptom subscale for the FACT-B. Breast Cancer Treat Res 1999;55:189-199.

US English
Copyright 1987, 1997
3/20/03 version

DRAFT

SWOG
S1200 AROMATASE INHIBITOR USAGE FORM

SWOG Patient ID ☐☐☐☐☐☐ **SWOG Study No.** S 1 2 0 0 **Registration Step** 1

Patient Initials __________ (L, F M)

Time Period: ☐ 12-week visit ☐ 24-week visit ☐ 52-week visit

Assessment Period Start Date: ☐☐ / ☐☐ / ☐☐☐☐

Assessment Period End Date: ☐☐ / ☐☐ / ☐☐☐☐

Institution/Affiliate ________________________ Physician ________________________

Instructions: Document AI usage for the reporting period marked above. Please submit this form within 7 days after each assessment. All dates are **MONTH, DAY, YEAR**. Explain any blank dates or fields in the **Comments** section. Place an ☒ in appropriate boxes.

The patient should complete Pages 2-5 of this form.

STATUS

Date of Last Contact or Death: ☐☐ / ☐☐ / ☐☐☐☐ **Vital Status:** ☐ Alive ☐ Dead *(submit Notice of Death)*

Has the patient had a cancer recurrence or new cancer diagnosed?

☐ No ☐ Yes *(submit S1200 Off Protocol Notice)*

Has the patient discontinued all AI therapy, without the intent of switching to a different AI?

☐ No ☐ Yes

Comments:

SWOG
S1200 AROMATASE INHIBITOR USAGE FORM

SWOG Patient ID ☐☐☐☐☐☐ **SWOG Study No.** S 1 2 0 0 **Registration Step** 1

Patient Initials __________ (L, F M)

Time Period: ☐ 12-week visit ☐ 24-week visit ☐ 52-week visit

Please answer the following questions about your aromatase inhibitor (hormonal therapy) use. Place an X in the appropriate boxes.

I. CURRENT AROMATASE INHIBITOR (HORMONAL THERAPY) USE

1. Which of the following aromatase inhibitor therapies are you CURRENTLY taking?

- ☐ Anastrozole (Arimadex)
- ☐ Letrozole (Femara)
- ☐ Exemestane (Aromasin)
- ☐ None, I stopped taking this kind of medication

2. When did you take your last pill for this therapy? ☐☐ / ☐☐ / ☐☐☐☐

3. How many pills have you missed in the past week for this therapy? ☐☐ *(Please provide one number, not a range.)*

continued on next page

SWOG Patient ID ☐☐☐☐☐☐ SWOG Study No. S 1 2 0 0 Registration Step 1

Patient Initials __________ (L, F M)

Time Period: ☐ 12-week visit ☐ 24-week visit ☐ 52-week visit

CURRENT AROMATASE INHIBITOR (HORMONAL THERAPY) USE, *continued*

4. Have you stopped taking this therapy?

☐ No **(Go to Part II on Page 4)**

☐ Yes→ Please indicate why you stopped *(Mark all that apply)*

- ☐ Cancer recurred
- ☐ Cost of the medication
- ☐ Lost or changed health insurance
- ☐ Doctor recommendation
- ☐ Participant choice
- ☐ Side effects → Indicate which kind of side effects *(Mark all that apply)*
  - ☐ Bone and/or joint pain
  - ☐ Osteoporosis
  - ☐ Thinning of bones
  - ☐ Nausea
  - ☐ Vomiting
  - ☐ Hot flashes
  - ☐ Weakness
  - ☐ Fatigue/tiredness
  - ☐ Headache
  - ☐ Insomnia/trouble sleeping
  - ☐ Dizziness
  - ☐ Drowsiness
  - ☐ High cholesterol
  - ☐ Weight gain
- ☐ Other, specify:______________________________

Thank you for completing this questionnaire

SWOG Patient ID ☐☐☐☐☐☐ SWOG Study No. S 1 2 0 0 Registration Step 1

Patient Initials _________ (L, F M)

Time Period: ☐ 12-week visit ☐ 24-week visit ☐ 52-week visit

II. PRIOR AROMATASE INHIBITOR (HORMONAL THERAPY) USE

5. Since your last visit, did you switch the kind of aromatase inhibitor therapy that you are taking from one kind to another?

☐ No **(Go to Part III on Page 5)**

☐ Yes → Why did you switch from this therapy? *(Mark all that apply)*

- ☐ Cost of the medication
- ☐ Lost or changed health insurance
- ☐ Doctor recommendation
- ☐ Participant choice
- ☐ Side effects → Indicate which kind of side effects *(Mark all that apply)*
  - ☐ Bone and/or joint pain
  - ☐ Osteoporosis
  - ☐ Thinning of bones
  - ☐ Nausea
  - ☐ Vomiting
  - ☐ Hot flashes
  - ☐ Weakness
  - ☐ Fatigue/tiredness
  - ☐ Headache
  - ☐ Insomnia/trouble sleeping
  - ☐ Dizziness
  - ☐ Drowsiness
  - ☐ High cholesterol
  - ☐ Weight gain
- ☐ Other, specify:______________________________

continued on next page

SWOG Patient ID ☐☐☐☐☐☐ **SWOG Study No.** S 1 2 0 0 **Registration Step** 1

Patient Initials __________ (L, F M)

Time Period: ☐ 12-week visit ☐ 24-week visit ☐ 52-week visit

III. MEDICATION ADHERENCE QUESTIONNAIRE (MAQ)

6. **Do you ever forget to take your aromatase inhibitor (hormonal therapy)?**........ ☐ Yes ☐ No
7. **Are you careless at times about taking your aromatase inhibitor (hormonal therapy)?**........ ☐ Yes ☐ No
8. **When you feel better, do you sometimes stop taking your aromatase inhibitor (hormonal therapy)?**........ ☐ Yes ☐ No
9. **Sometimes, if you feel worse after taking your aromatase inhibitor (hormonal therapy), do you stop taking it?**........ ☐ Yes ☐ No

Medication Adherence Questionnaire (MAQ) Morisky, Green and Levine. Medical Care 1986; 24(1): 67-74.

Thank you for completing this questionnaire

DRAFT

SWOG
S1200 FUNCTIONAL TESTING

SWOG Patient ID ☐☐☐☐☐☐ **SWOG Study No.** S 1 2 0 0 **Registration Step** 1

Patient Initials ____________ (L, F M) **Assessment Date:** ☐☐ / ☐☐ / ☐☐☐☐

Planned Assessment: ☐ Baseline ☐ 6-week visit ☐ 12-week visit ☐ 24-week visit ☐ 52-week visit

Institution/Affiliate ________________________________ Physician ______________________________

Instructions: Submit this form at time of registration and at 6 weeks, 12 weeks, 24 weeks , and 52 weeks post-registration. All dates are **MONTH, DAY, YEAR**. Explain any blank fields or blank dates in the **Comments** section. Place an ☒ in appropriate boxes. Circle **AMENDED** items in red and write **AMENDED** across top of form.

1) Grip strength (kPa): *Between the partially flexed fingers and the palm while the thumb applies counter pressure*

	Right	Left
1st Trial	☐☐.☐ kPa	☐☐.☐ kPa
2nd Trial	☐☐.☐ kPa	☐☐.☐ kPa
3rd Trial	☐☐.☐ kPa	☐☐.☐ kPa
Maximum value	☐☐.☐ kPa	☐☐.☐ kPa

NOTE: Ask patient to make 3 maximal voluntary contractions, with 1 minute between each. Give verbal encouragement and record each contraction (in kPa). If these readings are not within <10% of each other, then do further contractions, with 1 minute between each, until they are repeatable. Then record the maximum contraction.

2) 'Timed Get Up and Go' (seconds): ☐☐☐.☐

Comments:

DRAFT

SWOG
S1200 ADHERENCE TO ACUPUNCTURE

SWOG Patient ID ☐☐☐☐☐☐ **SWOG Study No.** S 1 2 0 0 **Registration Step** 1

Planned assessment: ☐ Registration to 1 week ☐ 1-3 weeks ☐ 3-6 weeks ☐ 6-12 weeks

Patient Initials __________ (L, F M)

Institution/Affiliate ________________________________ Physician ______________________________

Instructions: Submit this form after 1, 3, 6, and 12 weeks post-registration. All dates are **MONTH, DAY, YEAR**. Explain any blank dates or fields in the **Comments** section. Place an X in appropriate boxes. Circle **AMENDED** items in red and write **AMENDED** across top of form.

First Acupuncture treatment taken this period: ☐☐ / ☐☐ / ☐☐☐☐

Last Acupuncture treatment taken this period: ☐☐ / ☐☐ / ☐☐☐☐

Maximum number of expected Acupuncture treatments during this period: ☐☐

Number of completed Acupuncture treatments during this period: ☐☐

Number of scheduled Acupuncture treatments that were cancelled during this period: ☐☐

Was Acupuncture interrupted during this period? ☐ Yes ☐ No ☐ N/A

If Yes, specify date stopped: ☐☐ / ☐☐ / ☐☐☐☐

Specify reason stopped: ____

Was Acupuncture restarted? ☐ Yes ☐ No ☐ N/A

If Yes, specify date restarted: ☐☐ / ☐☐ / ☐☐☐☐

Specify reason restarted: ____

Did the patient receive all required Acupuncture treatments during this period? ☐ Yes ☐ No ☐ N/A

If No, specify reason: ☐ Improvement in symptoms ☐ Scheduling difficulties ☐ Other (explain below)

Comments:

DRAFT

SWOG
S1200 ADVERSE EVENT SUMMARY FORM

SWOG Patient ID ☐☐☐☐☐☐ **SWOG Study No.** S1200 **Registration Step** 1

Time Period: ☐ Registration to 1-week visit ☐ 1-week visit to 3-week visit ☐ 3-week visit to 6-week visit
☐ 6-week visit to 12-week visit ☐ 12-week visit to 24-week visit ☐ 24-week visit to 52-week visit

Patient Initials __________ (L, F M)

Institution/Affiliate ________________________________ Physician ____________________________

Instructions: Please submit this form at 1 week, 3 weeks, 6 weeks, 12 weeks, 24 weeks, and 52 weeks post-registration. **Document adverse events that occur within the time period marked above. Only document adverse events related to blinded protocol treatment (acupuncture/sham acupuncture) and joint pain/stiffness due to aromatase inhibitor treatment. Do not document other toxicities related to the aromatase inhibitor treatment.** Document the worst Grade seen during the reporting period. Do not code a condition existing prior to registration as an adverse event unless it worsens. Category lists may not include all adverse events from that category. Record any observed adverse events not listed on the blank lines at the end. All dates are **MONTH, DAY, YEAR**. Explain any blank dates or fields in the **Comments** section. Place an **X** in appropriate boxes.

ADVERSE EVENTS

Reporting period start date: __ __ / __ __ / __ __ __ __ *(Day 1 of this Cycle)*

Reporting period end date: __ __ / __ __ / __ __ __ __ *(Day one of next cycle. If final cycle, date of first visit or contact after resolution of acute adverse events.)*

Were adverse events assessed during this time period?
☐ No ☐ Yes, but no reportable adverse events occurred
☐ Yes, and reportable adverse events occurred (report below)

Date of Most Recent Adverse Event Assessment: __ __ / __ __ / __ __ __ __

CTC Adverse Event Term	CTCAE (4.0) Grade (1 - 5)	CTC Adverse Event Attribution Code*	Hospitalization (at least 24 hours)	CTC Adverse Event Term	CTCAE (4.0) Grade (1 - 5)	CTC Adverse Event Attribution Code*	Hospitalization (at least 24 hours)
Arthralgia	☐	☐	☐	Syncope	☐	☐	☐
Bruising	☐	☐	☐				
Infections and infestations - Other, specify	☐	☐	☐	**CTC Adverse Event Term, Other** *(specify using CTCAE 4.0 terminology)*			
Joint range of motion decreased	☐	☐	☐	____________	☐	☐	☐
Presyncope	☐	☐	☐	____________	☐	☐	☐
Skin infection	☐	☐	☐	____________	☐	☐	☐

* **Attribution codes:** 1-unrelated 2-unlikely 3-possible 4-probable 5-definite

Comments:

DRAFT

SWOG
S1200 SUPPLEMENTAL AGENTS REPORTING FORM

SWOG Patient ID ☐☐☐☐☐☐ **SWOG Study No.** S 1 2 0 0 **Registration Step** 1

Patient Initials ________ (L, F M)

Reporting period *(select one)*: ☐ Baseline ☐ Registration to 1-week visit ☐ 1-week visit to 3-week visit ☐ 3-week visit to 6-week visit ☐ 6-week visit to 12-week visit ☐ 12-week visit to 16-week contact ☐ 16-week contact to 20-week contact ☐ 20-week contact to 24-week visit ☐ 24-week visit to 52-week visit

Institution / Affiliate ________________ Physician ________________

Instructions: Please complete this form at baseline, 1 week, 3 weeks, 6 weeks, 12 weeks, 16 weeks, 20 weeks, 24 weeks and 52 weeks post-registration. Document supplemental agent usage information during the reporting period marked above. NOTE: For the prestudy reporting period, use the date of signed informed consent for reporting period start date and the date of registration for reporting period stop date. All dates are **MONTH, DAY, YEAR**. Explain any blank fields or blank dates in the **Comments** section. Place an X in appropriate boxes.

Reporting period start date: ☐☐ / ☐☐ / ☐☐☐☐

Reporting period stop date: ☐☐ / ☐☐ / ☐☐☐☐

Pain Medications:

Please list all pain medications taken during the past week for joint pain or musculoskeletal pain. Also indicate dosage per pill and frequency using the frequency codes below. If "other", specify type and dose units. If more than two "other" medications were taken, explain in comments section. If medications were not taken in pill form, explain in comments.

	Dosage (per pill)	Frequency
☐ Tylenol (acetaminophen)	☐☐☐ mg	☐
☐ Ibuprofen (e.g. Motrin)	☐☐☐ mg	☐
☐ Other NSAID, specify: ____________	☐☐☐☐ mg	☐
☐ Narcotics (specify in comments)	☐☐☐☐ mg	☐
☐ Other, specify: ____________	☐☐☐☐ mg	☐

Frequency codes:

1 = ≤ 1 pill per week
2 = 2-3 pills per week
3 = 4-6 pills per week
4 = 1 pill per day
5 = > 1 pill per day

continued on next page

DRAFT

SWOG
S1200 SUPPLEMENTAL AGENTS REPORTING FORM

SWOG Patient ID ☐☐☐☐☐☐ **SWOG Study No.** S 1 2 0 0 **Registration Step** 1

Patient Initials__________ (L, F M)

Reporting period *(select one):* ☐ Baseline ☐ Registration to 1-week visit ☐ 1-week visit to 3-week visit ☐ 3-week visit to 6-week visit ☐ 6-week visit to 12-week visit ☐ 12-week visit to 16-week contact ☐ 16-week contact to 20-week contact ☐ 20-week contact to 24-week visit ☐ 24-week visit to 52-week visit

Other Medications:

Please list any of the following other medications taken during the reporting period. Indicate dosage and frequency using the frequency codes below.

	Dosage	Frequency
☐ Statins (e.g., Lipitor)	☐☐☐☐	☐
☐ Aspirin	☐☐☐☐	☐
☐ Bisphosphonates, specify: ______________	☐☐☐☐	☐

Frequency codes:
1 = ≤ once per week
2 = 2-3 times per week
3 = 4-6 times per week
4 = once a day
5 = more than once a day

Steroids:

Please list all steroids taken during the reporting period. Indicate dosage and frequency using the frequency codes below.

	Dosage	Frequency
☐ Steroid pills<br>Type: ______________	☐☐☐☐	☐
☐ Steroid injection<br>Type: ______________	☐☐☐☐	☐
☐ Steroid cream<br>Type: ______________		☐

Frequency codes:
1 = ≤ once per week
2 = 2-3 times per week
3 = 4-6 times per week
4 = once a day
5 = more than once a day

Has the patient used therapeutic massage for joint symptoms since the last assessment? ☐ Yes ☐ No

Has the patient used any dietary supplement for joint symptoms since the last assessment? ☐ Yes ☐ No

Comments:

DRAFT

SWOG
S1200 ASSESSMENT OF BLINDING

SWOG Patient ID ☐☐☐☐☐☐ **SWOG Study No.** S 1 2 0 0 **Registration Step** 1

Patient Initials __________ (L, F M) **Date of Completion:** ☐☐ / ☐☐ / ☐☐☐☐

Planned assessment: ☐ 6-week visit ☐ 12-week visit ☐ 24-week visit ☐ 52-week visit

Instructions: You will be asked to complete this form at 6 weeks, 12 weeks, 24 weeks and 52 weeks post-registration. Thank you for your participation on this study.

1. Do you think you were receiving true acupuncture during your study participation?

☐ Yes ☐ No ☐ Not applicable (Waitlist control arm)

2. Did you get any acupuncture treatments outside of this clinical study?

☐ Yes ☐ No

If Yes, how many acupuncture treatments did you receive in the past 6 months outside of this clinical study? ☐☐

SWOG
S1200 OFF PROTOCOL NOTICE

SWOG Patient ID ☐☐☐☐☐☐ **SWOG Study No.** S 1 2 0 0 **Registration Step** 1

Patient Initials ____________ (L, F M)

Institution / Affiliate ________________________________ Physician ________________________________

Instructions: Submit this form within 2 weeks after completion (or discontinuation) of study intervention.
All dates are **MONTH, DAY, YEAR**. Explain any blank fields or blank dates in the **Comments** section.
Place an **X** in appropriate boxes.

Off Protocol Reason *(select one):*

☐ Completion of 12 weeks of study intervention

☐ Medically required, specify: ________________________________

☐ Patient refused, logistics (e.g., transportation) or scheduling conflicts, specify: ________________

☐ Patient refused, other reason, specify: ________________________________

☐ Evidence of any cancer, specify: ________________________________

☐ Death (submit Notice of Death form)

☐ Other, specify: ________________________________

Off Protocol Date

Date of completion, evidence of cancer, death or decision to discontinue therapy: ☐☐ / ☐☐ / ☐☐☐☐

Date of Last Contact or Death: ☐☐ / ☐☐ / ☐☐☐☐

Vital Status: ☐ Alive ☐ Dead (submit Notice of Death form)

Comments:

SWOG
FOLLOW UP FORM

SWOG Patient ID ☐☐☐☐☐☐ **SWOG Study No.** S☐☐☐☐ **Registration Step** ☐

Patient Initials ___________ (L, F M)

Institution / Affiliate ______________________________ Physician ______________________________

Participating Group: Group Name/Study No./Patient ID ______________ / ______________ / ______________

Instructions: Please submit at each follow up after completion of treatment until relapse or progression, at time of relapse or progression, and at protocol-specified intervals after relapse or progression. Also submit at time of diagnosis of second primary. All dates are **MONTH, DAY, YEAR**. Answer all questions and explain any blank fields or blank dates in the **Comments** section. Place an X in appropriate boxes. Circle AMENDED items in red.

VITAL STATUS

Vital Status: ☐ Alive ☐ Dead Date of last contact or death: ☐☐ / ☐☐ / ☐☐☐☐

If vital status is Dead, complete and submit Notice of Death form.

DISEASE FOLLOW UP STATUS

Has the patient had a documented clinical assessment for this cancer (since submission of the previous follow-up form)?

☐ No ☐ Yes If Yes, Date of Last Clinical Assessment: ☐☐ / ☐☐ / ☐☐☐☐

NOTICE OF FIRST RELAPSE OR PROGRESSION

Has the patient developed a first relapse or progression that has not been previously reported?

☐ No ☐ Yes If Yes, Date of Relapse or Progression: ☐☐ / ☐☐ / ☐☐☐☐

Site(s) of Relapse or Progression: ______________________________

NOTICE OF NEW PRIMARY

Has a new primary cancer or MDS (myelodysplastic syndrome) been diagnosed that has not been previously reported?

☐ No ☐ Yes If Yes, Date of Diagnosis: ☐☐ / ☐☐ / ☐☐☐☐

New Primary Site: ______________________________

NON-PROTOCOL TREATMENT

Has the patient received any non-protocol cancer therapy (prior to progression/relapse) not previously reported?

☐ No ☐ Yes If Yes, Date of First Non-Protocol Therapy: ☐☐ / ☐☐ / ☐☐☐☐

Agent Name(s): ______________________________

LONG TERM ADVERSE EVENT

Has the patient experienced (prior to treatment for progression or relapse or a second primary, and prior to non-protocol treatment) any severe (grade ≥ 3) long term toxicity that has not been previously reported?

☐ No ☐ Yes If Yes, Adverse Events and Grades: ______________________________

Comments:

SWOG
NOTICE OF DEATH

SWOG Patient ID ☐☐☐☐☐☐ **Most Recent SWOG Study No.** S☐☐☐☐

Patient Initials ____________ (L, F M)

Institution / Affiliate ________________________ Physician ________________________

Participating Group: Group Name/Study No./Patient ID ____________ / ____________ / ____________

Instructions: Answer all questions and explain any blank fields or blank dates in the **Comments** section.
Place an X in appropriate boxes. Circle **AMENDED** items in red.

Date of Death: ☐☐ / ☐☐ / ☐☐☐☐ (month / day / year)

CAUSES OF DEATH

Any cancer (select one):

☐ No ☐ Primary Cause ☐ Contributory ☐ Possible ☐ Unknown

If cancer was the primary cause or if cancer possibly or definitely contributed to death, and the patient had had multiple tumor types, specify those which were causes of death:

☐ Cancer of most recent SWOG study, specify cancer: ______________________

☐ Cancer of other SWOG study, specify cancer: ______________________

☐ Other cancer, specify: ______________________

Toxicity from disease related treatment (select one):

☐ No ☐ Primary Cause ☐ Contributory ☐ Possible ☐ Unknown

If Primary Cause, Contributory or Possible, specify treatment and toxicity:

Non-cancer and non-treatment related causes (select one):

☐ No ☐ Primary Cause ☐ Contributory ☐ Possible ☐ Unknown

If Primary Cause, Contributory or Possible, specify:

Autopsy? ☐ No ☐ Yes ☐ Unknown

Source(s) of death information:

☐ Autopsy report
☐ Medical record / Death certificate
☐ Physician
☐ Relative or friend
☐ Other, specify: ______________________

Comments:

19.0 APPENDIX

19.1 Acupuncture Description and Side Effects

19.2 Patient-Completed Questionnaires

19.3 Measurement of Blood and Urine Biomarkers

19.4 Training of Study Acupuncturists to the Study Protocol

19.5 CRA Functional Testing Training

19.6 Emergency Unblinding Guidelines

SWOG Authorized Document

19.1 Acupuncture Description and Side Effects

Description of True Acupuncture

The true acupuncture protocol and procedures employed will be devised with adherence to the Standards for Reporting of Controlled Trials in Acupuncture (STRICTA) recommendations. The acupuncture protocol was selected based on a standard Traditional Chinese Medicine (TCM) point prescription and informal practitioner query. A manualized protocol (*i.e.,* a protocol that is standardized but allows the Study Acupuncturist to modify treatments based on the patient's current joint symptoms) has been developed. The manualized protocol was selected as opposed to a fixed acupuncture point protocol to facilitate the systematic delivery of standardized acupuncture treatments and because it was shown to be effective in other acupuncture trials. *(79,80,96,143,150)* Patients will be scheduled for twice weekly 45-minute acupuncture sessions (30-minute acupuncture, 15-minute set-up) for 6 weeks (8-12 sessions) followed by 6 weekly treatments (4-6 sessions) for maintenance (12-18 sessions total over 12 weeks). The true acupuncture protocol includes standardized body and auricular treatments (Table 1). This point prescription is based on a standard TCM point prescription to treat "Bi Syndrome" and the National Acupuncture Detoxification Association (NADA) protocol applied to one ear to relieve pain and decrease stress. At every acupuncture visit, the patients assigned to true acupuncture will receive the full body acupuncture prescription and the auricular acupuncture-NADA protocol in one ear (to be alternated at each visit). (*151)* In addition, each session will include a joint-specific point prescription tailored to up to four of the patient's most painful joints, including knees, fingers, lumbar area, shoulders, hips, and wrists. *(79, 144)* The anatomic site selection may vary between visits depending on the patient's current symptoms.

Acupuncture needles used will be Mac™ single-use, sterile, and disposable. The full body acupuncture needles will be 1 inch, 1.5 inches or 3 inches and 34-gauge (manufactured by Tian Jin Haing Lin Sou Won Medical Instrument Co., Ltd; distributed by Mac Co., Roslyn Heights, New York, USA) and auricular needles will be 15mm and 38-gauge (Seirin-America, Inc., MA). The needling protocol will consist of first swabbing all points with alcohol and needling auricular points, then needling full-body points. The needles will be inserted to the proper needling depth as determined by standard point locations and to elicit a *de qi* sensation at all standardized full body acupuncture points. *(152)* The needles will remain *in situ* for 20-25 minutes during which the Study Acupuncturist will return to stimulate the needles once, utilizing even needle technique in order to re-elicit the *de qi* sensation. If needed, to access joint-specific points that are not accessible in the supine position, needles may be inserted after the first set, at the proper depth and angle, eliciting a *de qi* sensation, and retained for 10 minutes. No electrical stimulation will be used. One licensed Study Acupuncturist will perform all treatments. In addition, up to 2 licensed acupuncturists will be trained in administering the protocol in the event of schedule conflicts. At 6, 12, 24, and 52 weeks, to assess the adequacy of blinding, patients will give an opinion about their treatment assignment. After the 24-week visit, patients will receive a voucher for 10 free acupuncture sessions to be administered by the study acupuncturist(s) used off-study at her convenience. Any additional acupuncture treatments received off-study will be recorded at 24 and 52 weeks. All of this information will be recorded on the **S1200** Assessment of Blinding Form (Form #20574).

SWOG Authorized Document

Table 1. True Acupuncture Points

Point Name	Location
**Full body acupuncture points (given at each visit)**	
SJ 5-*wai guan* (TB 5)	2 cun* proximal to the dorsal crease of the wrist, on the line connecting SJ-4 (located between the 4th and 5th metacarpal bones, in the depression proximal to the 4th metocarpophalangeal joint) and the tip of the olecranon, between the radius and the ulna
GB 41-*zulin qi*	Posterior to the fourth metatarsalphalangeal joint, in the depression lateral to the tendon of m. extensor digiti minimi of the foot
GB 34-*yang ling quan*	In the depression anterior and inferior to the head of the fibula
LI 4-*he gu*	On the dorsum of the hand, between the 1st and 2nd metacarpal bones, approximately in the middle of the 2nd metacarpal bone on the radial side
ST-41-*jie xi*	On the dorsum of the foot, at the midpoint of the transverse crease of the ankle joint, in the depression between the tendons of m. extensor digitorum longus and hallicus longus, approximately at the level of the tip of the external maleolus
KD 3-*tai xi*	In the depression between the tip of the external maleolus and the anterior side of the tendon achilles
**Auricular acupuncture points (given at each visit in one ear and alternating ears with each treatment)**	
Shen men	At bifurcating point between superior and inferior antihelix cru, and lateral 1/3 of the triangular fossa
Kidney	On the lower border of the inferior antihelix crus, directly above the small intestine point
Liver	At the posterior aspect of the stomach and duodenum points
Upper lung	Directly above the heart point in the central depression of the cavum conchae
Sympathetic	At the end of the inferior antihelix crux
**Joint-specific point protocols (tailored to up to 3 of the patient's most painful areas with each treatment)**	
Knee:	
SP-9	On the lower border of the medial condyle of the tibia in the depression posterior and inferior to the medial condyle of the tibia
SP-10	With knee flexed, 2 cun above the superior medial border of the patella on the bulge of the medial portion of quadriceps femoris (vastus medialis)
ST-34	With knee flexed, 2 cun above the superior lateral border of the patella on the line connecting with the anterior superior iliac spine (ASIS)
Fingers:	
SI-3	When a loose fist is made, the point on the ulnar end of the crease in front of the metacarpalphalageal joint of the little finger, at the junction of the two skins
Ba xie	On the dorsum of the hand, at the junction of the two skins, eight in all, making a loose fist to locate the points
LI-3	When a loose fist is made, the point on the radial side of the index finger, in the depression proximal to the head of the 2nd metacarpal bone

Joint-specific point protocols (tailored to up to 3 of the patient's most painful areas with each treatment) (contd.)	
Lumbar:	
Du-3	Below the spinous process of the fourth lumbar vertebrae, at the level with the crista iliac
Du-8	Below the spinous process of the ninth thoracic vertebrae
UB-23	1.5 cun lateral to Du-4 (below the spinous process of the second lumbar vertebrae) at the level of the lower border of the spinous process of the second lumbar vertebrae
Shoulder:	
LI-15	Antero-inferior to the acromion, on the upper portion of m. deltoideus
SJ-14	On the shoulder, posterior to LI-15, in the depression inferior and posterior to the acromium
SI-10	Directly above the posterior end of the axillary fold, in the depression inferior to the scapular spine
Hip:	
GB-30	At the junction of the lateral 1/3 and medial 2/3 of the distance between the prominence of the greater trochanter and the hiatus of the sacrum
GB-39	3 cun above the tip of the external malleolus on the posterior border of the fibula
Wrist:	
SI-5	At the ulnar end of the transverse crease on the dorsal aspect of the wrist, in the depression between the styloid process and the ulnar and triquetral bone
SJ-4	On the transverse crease of the dorsum of the wrist, in the depression lateral to the tendon of fm. extensor digitorum communis
LI-5	When the thumb is tilted upward, the point in the depression between the tendons of m. extensor pollicis longus and brevis on the radial side of the wrist

* A cun is a measurement used in finding acupuncture points. As each person has different body dimensions, a cun is defined according to the person whose body is to be treated. It is equivalent to the width of the distal phalanx of the person's thumb, in the middle, at the crease.

Description of Sham Acupuncture

The sham acupuncture intervention, which is a control for acupoint specificity, will use superficial needle insertion at body locations not recognized as true acupoints. Similar to the true acupuncture arm, patients will be scheduled for twice weekly 45-minute acupuncture sessions (30-minute sham acupuncture, 15-minute set-up) for 6 weeks (8-12 sessions) followed by 6 weekly treatments (4-6 sessions) for maintenance (12-18 sessions total over 12 weeks). The sham acupuncture protocol includes full body and joint-specific treatments and an auricular sham intervention (Table 2). At every acupuncture visit, the patients assigned to sham acupuncture will receive the full body sham and auricular sham acupuncture prescriptions. In addition, each session will include a joint-specific sham point prescription tailored to up to four of the patient's most painful joints, including knees, fingers, lumbar area, shoulders, hips, and wrists. The anatomic site selection may vary between visits depending on the patient's current symptoms.

Acupuncture needles used will be Mac™ single-use, sterile, and disposable with plastic guide tube. The sham acupuncture needles will be 0.5 inch 34-gauge (manufactured by Tian Jinhaing Lin Sou Won Medical Instrument Co., Ltd; distributed by Mac Co., Roslyn Heights, New York, USA). The needling protocol will consist of first swabbing all points with alcohol and needling full body and joint-specific sham points, inserting the needle by tapping in just to the upper level of the guide tube, at a transverse angle. The auricular

SWOG Authorized Document

sham procedure will include application of 3 adhesives to selected points on the ear (Table 2). The auricular adhesives are latex-free and will be loosely attached and pressed to the sham points on the helix of the auricle. The needles and auricular adhesives will remain in situ for 20-25 minutes during which the Study Acupuncturist will return to check the patient and lightly touch each needle handle and auricular adhesive in order to mimic the true intervention. No electrical stimulation will be used. The same Study Acupuncturists will administer both the true and sham acupuncture interventions. At 24 weeks, to assess the adequacy of blinding, patients will give an opinion about their treatment assignment. After the 24-week visit, patients will receive a voucher for 10 free true acupuncture sessions to be administered by the study acupuncturist(s) and used off-study at her convenience. Any additional acupuncture treatments received off study will be recorded at 24 and 52 weeks. All of this information will be recorded on the **S1200** Assessment of Blinding and Additional Acupuncture Form (Form #20574).

Table 2. Sham Acupuncture Points

Point Name	Location
**Full body sham acupuncture points (given at each visit)**	
Sham 1	On the lateral side of the left forearm, near the elbow, 3 cun below the olecranon, 0.5 cun toward the anterior of the small intestine meridian
Sham 2	On the lateral side of the right forearm, near the elbow, 3 cun below the olecranon, 0.5 cun toward the anterior of the small intestine meridian
Sham 3	At the lower border of the medial condyle of the left tibia, 1 cun anterior and superior to *xi guan* (Liv 7) of the liver meridian
Sham 4	At the lower border of the medial condyle of the right tibia, 1 cun anterior and superior to *xi guan* (Liv 7) point of the liver meridian
**Auricular (A) sham acupuncture points (given at each visit in one ear and alternating ears with each treatment)**	
A Sham 1	On the helix of the auricle between helix point #5 and #6
A Sham 2	On the helix of the auricle between helix point #4 and #3
A Sham 3	On the helix of the auricle between the ear apex and helix point #1
**Joint-specific sham point protocols (tailored to up to 3 of the patient's most painful areas with each treatment)**	
Knee	2 cun above sham 3 or 4, respectively
Fingers/ Wrist	On the lateral side of the left and right forearm, near the elbow, 5 cun below the olecranon, 0.5 cun toward the anterior of the small intestine meridian
Lumbar	On the back at the level of thoracic vertebra 8, 5 cun from the center of the spine, 2 cun from the outer channel of the urinary bladder meridian (needled bilaterally)
Shoulder	On the lateral side of the left and right arm, above the elbow, 3 cun above the olecranon, 0.5 cun toward the anterior of the small intestine meridian
Hip	On the thigh, approximately 4 cun above the patella, 1 cun away from the anterior of the gall bladder meridian

Description of Waitlist Control

Patients randomized to the waitlist control group will undergo the scheduled evaluations at baseline, Week 2, Week 4, Week 6, Week 12, Week 24, and Week 52. Patients will be allowed to take acetaminophen and NSAIDs as needed for their joint symptoms, which will be documented as analgesic use at scheduled time points and captured on the **S1200** Supplemental Agents Reporting Form. They must agree not to join any other clinical trial for the treatment of AI-related arthralgias while participating in this study. After the 24-week visit, patients will receive a voucher for 10 free acupuncture sessions to be administered by the study acupuncturist(s) and used off-study at her convenience. Any additional acupuncture treatments received off-study will be recorded at 24 and 52 weeks. All of this information will be recorded on the **S1200** Assessment of Blinding and Additional Acupuncture Form (Form #20574).

SWOG Authorized Document

19.2 Patient-Completed Questionnaires

Joint symptoms (Brief Pain Inventory-Short Form [BPI-SF], Western Ontario and McMaster Universities Osteoarthritis [WOMAC] index for the hips and knees, Modified-Score for the Assessment and Quantification of Chronic Rheumatoid Affections of the Hands [M-SACRAH], and PROMIS Pain Impact-Short Form [PROMIS PI-SF]) and quality of life (Functional Assessment of Cancer Therapy-Endocrine Subscale [FACT-ES] will be collected from self-administered questionnaires given at baseline, 6 weeks, 12 weeks, 24 weeks, and 52 weeks. *(137,139, 140,155)*

The BPI-SF is a 14-item questionnaire which asks patients to rate pain over the prior week and the degree to which it interferes with activities on a 0 to 10 scale. *(137)* Severity is measured as average pain, pain right now, worst pain, and least pain. The BPI-SF responds to both behavioral and pharmacological pain interventions and has been well-validated. Note that we consider a reduction of two or more points on the BPI-SF worst pain item (#2) to correspond to a clinically meaningful decrease in pain.

The WOMAC index Version 3.1 is a validated questionnaire for assessing osteoarthritis of the knees or hips and consists of 24 questions related to three subscales: pain (0-50), stiffness (0-20), and physical function (0-170). *(139)* Each question is answered by the patients along a standardized 10-point scale with terminal descriptors. The WOMAC (V3.1) osteoarthritis index has been tested extensively for validity, reliability, and responsiveness for measuring changes in symptoms after different interventions. Although originally developed as a standardized assessment to evaluate osteoarthritis, the WOMAC has been used as a generalized symptom scale to evaluate musculoskeletal pain from conditions such as rheumatoid arthritis, total knee replacements, femoral shaft fracture, and osteonecrosis of the femoral head. *(155-159)* The M-SACRAH consists of three domains assessing pain, stiffness, and functional status in patients suffering from hand osteoarthritis and rheumatoid arthritis, answered on 10-point scale. *(140)*

The Functional Assessment of Cancer Therapy-General (FACT-G) consists of four subscales including Physical, Social/Family, Emotional, and Functional Well-Being. We will focus on just Physical and Functional Well-Being for this trial. We will also use the Endocrine Subscale (ES) to monitor endocrine side effects caused by AIs, such as hot flashes and vaginal symptoms.*(155)* ACT scales have five response levels (“not at all” to “very much”), where higher scores reflect better well-being and fewer symptom problems.

SWOG Authorized Document

19.3 Measurement of Blood and Urine Biomarkers

a. Blood Analyses

Patients will provide blood specimens at baseline, 6 weeks, 12 weeks, and 24 weeks for the measurement of serum hormone biomarkers (estradiol, FSH, LH) and inflammatory biomarkers (IL-6, IL-12, TNFα, CRP). Blood (~20 cc in two 10-ml red-top tubes) will be collected by standard venipuncture technique. Blood in the red top tubes is allowed to clot for 30 minutes to 1 hour (maximum) at room temperature, and centrifuged at 3,000 x g for 20 minutes immediately after clotting, or refrigerated or placed on ice for no more than 6 hours, and centrifuged at 3,000 x g for 20 minutes. Using a pipette, serum is then transferred to eight 1.8-ml Cryovials (1 ml each) and any extra serum should be aliquoted in a separate Cryovial.

Radioimmunoassays (RIAs) will be used to measure serum estradiol, FSH, and LH. Serum IL-6, IL-12, and TNFα will be determined by enzyme-linked immunosorbent assay (ELISA) using a commercially available kit (R&D Systems Inc., Minneapolis, MN). Serum hsCRP will be measured on the COBAS INTEGRA 400 plus system (Roche Diagnostic, Indianapolis, IN). All serum assays will be performed according to the manufacturer's instructions and conducted at the Irving Center for Clinical Research (ICCR) at CUMC by Dr. S. Cremers.

b. Urine Analysis

At baseline, 6 weeks, 12 weeks, 24 weeks, and 52 weeks, approximately 10 ml of urine will be collected. Using a pipette, urine is then transferred to five 1.8-ml Cryovials (1.5 ml each) and any extra urine should be aliquoted in a separate Cryovial. The urine specimens will be sent in bulk shipment at the end of study on dry ice to the Sports Medicine Research and Testing Laboratory in Salt Lake City. The urine assays will be performed using high performance liquid chromatography (HPLC) tandem mass spectrometry.

c. Genotyping Analysis

At baseline, patients will provide additional blood specimens for plasma/buffy coat to be used for genotyping analyses. Approximately 10 ml of blood will be drawn in a 10-ml EDTA purple-top tube (after the serum sample so that the plasma can be mixed without delay). Just prior to collection of the specimen, it is important to rotate the 10-ml purple-top tube to allow the EDTA solution to coat the inside of the tube. Following collection of the blood, the sample must be inverted gently 10-15 times to mix well.

Genomic DNA will be extracted from the buffy coat (gray layer of WBC) using standard RNase/proteinase K technique. All samples will be genotyped using the ABI PRISM 7900HT Sequence Detection System (Applied Biosystems, Foster City, CA) in a 96-well format. The fluorogenic 5' nuclease or TaqMan assay will be used to determine genotype in the *CYP19A1* aromatase gene. Genotyping will be done by laboratory personnel blinded to treatment assignment. All genotyping studies will be conducted in the laboratory of Dr. R. Santella (CUMC).

SWOG Authorized Document

19.4 Training of Study Acupuncturists to the Study Protocol

A web-based site initiation visit and formal training of the Study Acupuncturists and Clinical Research Associates (CRAs) administering the functional testing at each site will be performed at the time of study activation. At least two acupuncturists licensed with the appropriate certifications for the state at each site will be trained and certified annually in administering the protocol and will perform all treatments. Each site already has experience in conducting acupuncture clinical trials.

CUMC-supplied study materials, including single-use, sterile, and disposable acupuncture needles, acupuncture tables, a training manual and online training module of the point protocols for both the true and sham acupuncture interventions and a demonstration of the grip strength and 'Timed Get Up and Go' (TGUG) testing, will be provided to each participating site. In addition, sites will be supplied with standardized dynamometers for grip strength testing and stop watches for the 'Timed Get Up and Go' test.

The acupuncture training manual will consist of the following:

- Written point protocols and anatomic renderings of all true and sham acupuncture points
- Written description about basic clean needle technique and administration of true and sham acupuncture interventions
- Written explanation for filling out case report forms done by the Study Acupuncturist

The acupuncture online training module will consist of the following:

- Video recording of the true and sham acupuncture points on a human volunteer
- Video recording of true acupuncture needling intervention in 1-2 selected body points and the auricular points
- Video recording of the sham acupuncture needling intervention in 1-2 selected body points and auricular points (taping)

SWOG Authorized Document

19.5 CRA Functional Testing Training

The functional testing online training module will consist of the following:

- Video recording of how to perform the dynamometer measurements for grip strength testing
- Video recording of the components of the 'Timed Get Up and Go' test including how to use the stopwatch and a recording of a human volunteer enacting the test

SWOG Authorized Document

19.6 Emergency Unblinding Guidelines

a. **General Considerations**

In this intervention trial, **S1200** participants AND study staff are blinded to two of the three treatment assignments, True Acupuncture or Sham Acupuncture (double blind conditions). Guidelines and Procedures for unblinding in emergency situations are given below (see Sections 19.6c and d).

In general, participants SHOULD NOT be unblinded to the treatment assignment unless a condition exists where the knowledge of the participant's treatment assignment would directly influence or affect her immediate care, or if an emergency situation arises.

Emergency unblinding of all **S1200** treatment assignments will be done by the Washington Poison Center (WPC) with medical advice from a panel of two resource physicians appointed for this study. Calls for information about the treatment assignment, or the study in general, should be directed to the SWOG Statistical Center.

b. **Guidelines for Discontinuation of Study Intervention**

(Unblinding is NOT appropriate when acupuncture is discontinued.) The following events MAY require PERMANENT discontinuation of Coded acupuncture:

- Evidence of new cancer or cancer recurrence at any time.
- Unacceptable toxicity (as defined in Section 8.0)
- A participant who becomes pregnant while on treatment will be removed from protocol treatment
- Cumulative delay ≥ 30 consecutive days after study registration
- Completion of 12 weeks of study intervention (acupuncture).

Permanent discontinuation of the Coded intervention for any reason must be documented on the **S1200** Off Protocol Form (Form #9707).

The following events MAY require TEMPORARY discontinuation of Coded Intervention:

- Treatment of and/or hospitalization for a medical problem;
- Assessment of symptoms or side effects potentially related to Coded Intervention.

Temporary discontinuation of the Coded Intervention for any reason must be documented on the **S1200** Off Protocol Form (Form #9707).

Refer to Section 7.0 (Procedures and Study Plan) and Section 8.0 (Safety Monitoring) of this protocol for details on discontinuation of study intervention.

SWOG Authorized Document

c. **Guidelines for Emergency Unblinding of Treatment Assignment**

The following event MAY require emergency unblinding of treatment assignment:

- A compelling medical need as determined by a physician, e.g., occurrence of a severe or life-threatening reaction, inclusive of an adverse drug reaction, which may have been attributable to treatment assignment, or existence of a condition where the knowledge of the participant's treatment assignment would directly influence or affect her immediate care;

d. **Procedure for Emergency Unblinding**

The procedure for unblinding the treatment assignment for a participant is as follows:

- All unblinding must be done by the registering physician or designee.

- Call the Washington Poison Control (WPC) collect at 206/526-2121or at 800/732-6985 if calling from within Washington State. The WPC is accessible 24 hours per day, 365 days per year for unblinding calls. Informational calls should be directed to the Data Operations Center in Seattle during standard business hours.

- Provide the WPC with the following information:

  Study number: **S1200**
  SWOG patient number
  Participant name
  Name and telephone number of the caller
  Reason unblinding is required

- Unblinding for a "compelling medical need" must be authorized by a physician designated as a resource physician for this protocol.

The treating physician (or designee) would provide the WPC with the information needed to determine if unblinding is required for the participant. The WPC would contact the resource physician, provide the required information, and obtain the authorization to unblind, if necessary. Based on the decision of the resource physician, the WPC would call the treating physician with either the unblinded treatment assignment or a treatment recommendation from the resource physician.

If a resource physician cannot be reached by the WPC, treatment of the participant should proceed as if the patient received True Acupuncture.

Unblinding of treatment assignment for any reason must be documented on the **S1200** Off Protocol Form (Form #9707). All unblinded patients are taken off treatment and followed per the requirements of SWOG.

Any questions regarding unblinding may be directed to one of the following resource physicians:

SWOG Authorized Document

Dawn Hershman, M.D., M.S. (Medical Oncology)
Columbia University
161 Fort Washington Avenue
10-1068
New York, NY 10032
Phone:212/305-1945
Fax:212/305-0178
E-mail:dlh23@columbia.edu

Katherine D. Crew, MD, MS (Medical Oncology)
Columbia University
161 Fort Washington Avenue
10-1072
New York, NY 10032
Phone:212/305-1732
FAX:212/305-0178
E-mail:kd59@columbia.edu

SWOG Authorized Document
