# Supplementary material for: Comparison of Acupuncture vs Sham Acupuncture or Waiting List Control in the Treatment of Aromatase Inhibitor–Related Joint Pain: A Randomized Clinical Trial
Source: JAMA Netw Open. 2022 Nov 11;5(11):e2241720. doi: 10.1001/jamanetworkopen.2022.41720 (PMC9652759; doi:10.1001/jamanetworkopen.2022.41720)
Supplement: Supplement 2. — eFigure. CONSORT Diagram Showing the Disposition of Patients With Respect to the Examination of the Primary Endpoint, the 6-Week Breast Pain Index-Short Form (BPI-SF) Worst Pain Score eTable 1. Observed and Fitted Group Mean Results for Brief Pain Inventory and PROMIS Pain Intensity Scores at Weeks 6, 12, 24, and 52 in Each Group eTable 2. Determination of Best Model Fit for Longitudinal Data Analyses eTable 3. Grip Strength, Maximal Contraction Over Left and Right Hand, by Randomized Arm and Time Point eTable 4. Grip Strength, Change in Maximal Contraction Between Follow-up and Baseline by Randomized Arm and Time Point eTable 5. Timed Get Up and Go, by Randomized Arm and Time Point eTable 6. Timed Get Up and Go, Change Between Follow-up and Baseline, by Randomized Arm and Time Point [file jamanetwopen-e2241720-s002.pdf]

Supplementary Online Content

Hershman DL, Unger JM, Greenlee H, et al. Comparison of acupuncture vs sham acupuncture or waiting list control in the treatment of aromatase inhibitor–related joint pain: a randomized clinical trial. *JAMA Netw Open*. 2022;5(11):e2241720. doi:10.1001/jamanetworkopen.2022.41720

eFigure. CONSORT Diagram Showing the Disposition of Patients With Respect to the Examination of the Primary Endpoint, the 6-Week Breast Pain Index-Short Form (BPI-SF) Worst Pain Score

eTable 1. Observed and Fitted Group Mean Results for Brief Pain Inventory and PROMIS Pain Intensity Scores at Weeks 6, 12, 24, and 52 in Each Group

eTable 2. Determination of Best Model Fit for Longitudinal Data Analyses

eTable 3. Grip Strength, Maximal Contraction Over Left and Right Hand, by Randomized Arm and Time Point

eTable 4. Grip Strength, Change in Maximal Contraction Between Follow-up and Baseline by Randomized Arm and Time Point

eTable 5. Timed Get Up and Go, by Randomized Arm and Time Point

eTable 6. Timed Get Up and Go, Change Between Follow-up and Baseline, by Randomized Arm and Time Point

This supplementary material has been provided by the authors to give readers additional information about their work.

© 2022 Hershman DL et al. *JAMA Network Open.*

eFigure. CONSORT Diagram Showing the Disposition of Patients With Respect to the Examination of the Primary Endpoint, the 6-Week Breast Pain Index-Short Form (BPI-SF) Worst Pain Score*

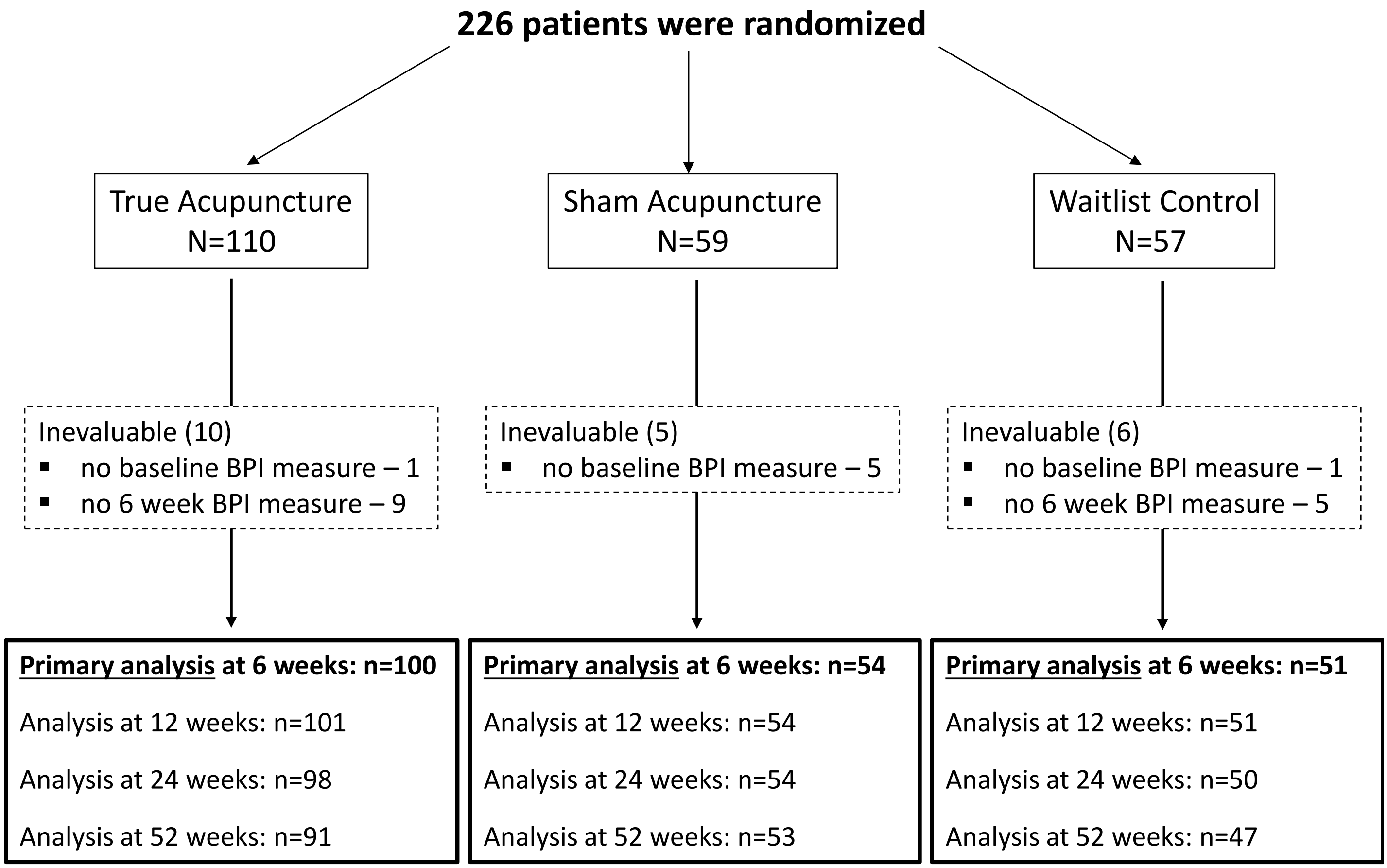

*About 890 patients approached to participate in study

© 2022 Hershman DL et al. *JAMA Network Open.*

eTable 1. Observed and Fitted Group Mean Results for Brief Pain Inventory and PROMIS Pain Intensity Scores at Weeks 6, 12, 24, and 52 in Each Group

	True Acupuncture		Compared to Control				
FU Wk	N	Observed, Baseline[1] v FU (Mean, 95% CI)	Vs. Control Arm	N	Observed, Baseline[1] v FU (Mean, 95% CI)	Model-Fitted Difference[2]	P-value[2]
Worst Pain							
6	100	6.84 (6.54-7.14) v 4.79 (4.36-5.22)	Sham	54	6.41 (5.98-6.82) v 5.48 (4.78-6.18)	0.96 (0.23,1.68)	.01
			Waitlist	51	6.41 (5.98-6.84) v 5.49 (4.81-6.17)	0.98 (0.24,1.72)	.01
12	101	6.82 (6.52-7.12) v 4.53 (4.05-5.01)	Sham	54	6.41 (5.98-6.84) v 5.04 (4.33-5.74)	0.72 (-0.05,1.48)	.07
			Waitlist	51	6.47 (6.06-6.89) v 6.29 (5.73-6.86)	1.97 (1.19,2.75)	<.001
24	98	6.79 (6.49-7.10) v 4.65 (4.12-5.18)	Sham	54	6.39 (5.97-6.81) v 5.00 (4.39-5.61)	0.51 (-0.30,1.33)	.22
			Waitlist	50	6.46 (6.03-6.89) v 5.72 (5.04-6.39)	1.20 (0.36,2.04)	<.001
52	91	6.77 (6.45-7.08) v 4.05 (3.50-4.61)	Sham	53	6.38 (5.95-6.80) v 4.92 (4.28-5.57)	1.08 (0.24,1.91)	.01
			Waitlist	47	6.40 (5.95-6.86) v 4.85 (4.09-5.61)	0.99 (0.12,1.86)	.03
Average Pain							
6	100	4.90 (4.57-5.23) v 3.50 (3.12-3.88)	Sham	54	4.54 (4.06-5.02) v 3.93 (3.40-3.45)	0.62 (0.07,1.17)	.03
			Waitlist	51	4.76 (4.34-5.18) v 4.14 (3.65-3.68)	0.72 (0.16,1.29)	.01
12	101	4.87 (4.54-5.20) v 3.00 (2.61-3.38)	Sham	53	4.43 (3.98-4.89) v 3.62 (3.06-4.18)	0.81 (0.19,1.43))	.01
			Waitlist	51	4.76 (4.34-5.18) v 4.33 (3.80-4.87)	1.39 (0.77,2.01)	<.001
24	98	4.82 (4.49-5.14) v 3.27 (2.86-3.67)	Sham	54	4.52 (4.04-4.99) v 3.52 (3.02-4.02)	0.37 (-0.28,1.02)	.26
			Waitlist	50	4.80 (4.38-5.22) v 3.92 (3.29-4.55)	0.65 (-0.01,1.32)	.05
52	91	4.82 (4.47-5.17) v 3.06 (2.10-3.52)	Sham	53	4.55 (4.06-5.03) v 3.23 (2.74-3.71)	0.29 (-0.36,0.95)	.37
			Waitlist	47	4.81 (4.36-5.25) v 3.08 (2.46-3.71)	0.02 (-0.66,0.70)	.96
Pain Interference							
6	100	4.24 (3.86-4.62) v 2.55 (2.11-2.98)	Sham	54	3.85 (3.34-4.37) v 3.03 (2.44-3.62)	0.73 (0.11,-1.35)	.02
			Waitlist	51	4.01(3.54-4.48) v 3.07 (2.47-3.67)	0.65 (0.02,1.29)	.04
12	101	4.24 (3.86-4.62) v 2.44 (2.00-2.88)	Sham	54	3.85 (3.34-4.37) v 2.40 (1.84-2.96)	0.21 (-0.40,0.81)	.50
			Waitlist	51	4.01 (3.54-4.48) v 3.31 (2.69-3.94)	1.00 (0.38,1.61)	.002
24	98	4.16 (3.77-4.56) v 2.43 (1.98-2.89)	Sham	54	3.63 (3.15-4.11) v 2.33 (1.77-2.89)	0.23 (-0.41,0.86)	.48
			Waitlist	50	4.03 (3.52-4.54) v 3.18 (2.55-3.82)	0.84 (0.19,1.49)	.01
52	91	4.08 (3.67-4.49) v 2.16 (1.69-2.63)	Sham	53	3.76 (3.23-4.28) v 2.54 (2.01-3.06)	0.58 (0.00,1.16)	.05
			Waitlist	46	3.91 (3.38-3.33) v 2.38 (1.75-3.00)	0.33 (-0.28,0.93)	.29
Pain Severity							
6	100	4.71 (4.41-5.01) v 3.21 (2.85-3.57)	Sham	54	4.75 (5.32-5.19) v 3.75 (3.21-5.28)	0.55 (0.00,1.11)	.05
			Waitlist	51	4.64 (4.25-5.03) v 3.82 (3.26-4.37)	0.72 (0.15,1.29)	.01
12	101	4.71 (4.41-5.01) v 2.89 (2.52-3.26)	Sham	54	4.75 (5.32-5.19) v 3.41 (2.86-3.95)	0.54 (-0.04,1.12)	.07
			Waitlist	51	4.64 (4.25-5.03) v 4.25 (3.72-4.78)	1.44 (0.85,2.04)	<.001
24	98	4.59 (4.28-4.90) v 3.09 (2.97-3.50)	Sham	54	4.61 (4.16-5.05) v 3.41 (2.91-3.91)	0.33 (-0.30,0.96)	.31
			Waitlist	50	4.53 (4.13-4.92) v 3.89 (3.30-4.49)	0.83 (0.19,1.48)	.01

© 2022 Hershman DL et al. *JAMA Network Open.*

52	91	4.59 (4.27-4.92) v 2.82 (2.38-3.25)	Sham	53	4.62 (4.16-5.07) v 3.17 (2.69-3.65)	0.33 (-0.28,0.95)	.29
			Waitlist	47	4.53 (4.11-4.95) v 3.07 (2.45-3.68)	0.28 (-0.36,0.92)	.39
Worst Stiffness							
6	100	6.65 (6.26-7.04) v 4.46 (3.94-4.98)	Sham	54	6.59 (6.03-7.16) v 5.50 (4.86-6.14)	1.00 (0.21,1.78)	.01
			Waitlist	51	6.68 (6.26-7.10) v 5.53 (4.91-6.14)	1.11 (0.31,1.90)	.007
12	100	6.65 (6.26-7.04) v 4.35 (3.82-4.88)	Sham	54	6.59 (6.03-7.16) v 5.07 (4.47-5.68)	0.73 (-0.02,1.49)	.06
			Waitlist	51	6.68 (6.26-7.10) v 6.12 (5.59-6.64)	1.80 (1.03,2.57)	<.001
24	98	6.61 (6.19-7.04) v 4.51 (4.02-5.00)	Sham	54	6.52 (6.00-7.04) v 4.92 (4.31-5.32)	0.46 (-0.31,1.23)	.24
			Waitlist	50	6.62 (6.19-7.04) v 5.56 (4.88-6.23)	1.05 (0.25,1.84)	.01
52	91	6.58 (6.13-7.03) v 4.33 (3.78-4.87)	Sham	53	6.68 (6.19-7.17) v 4.72 (3.98-5.46)	0.34 (-0.47,1.15)	.41
			Waitlist	46	6.67 (6.21-7.13) v 4.70 (3.99-5.40)	0.35 (-0.51,1.20)	.42
PROMIS Pain Intensity							
6	101	61.32 (60.33-62.31) v 55.93 (54.53-57.34)	Sham	54	59.71 (58.21-61.32) v 57.63 (56.04-59.21)	2.75 (0.86,4.64)	.005
			Waitlist	51	59.08 (57.61-60.56) v 56.68 (54.96-58.40)	2.24 (0.30,4.18)	.02
12	102	61.30 (60.31-62.-28) v 54.72 (53.25-56.19)	Sham	54	59.71 (58.21-61.21) v 56.19 (54.47-57.91)	2.58 (0.52,4.65)	.01
			Waitlist	51	59.08 (57.61-60.56) v 57.41 (55.43-59.40)	4.25 (2.13,6.37)	<.001
24	99	61.30 (60.27-62.34) v 55.40 (53.84-56.96)	Sham	54	59.54 (58.10-60.98) v 54.98 (53.08-56.89)	0.92 (-1.36,3.19)	.43
			Waitlist	50	59.16 (57.67-60.98) v 56.53 (54.16-58.90)	2.75 (0.40,5.09)	.02
52	93	61.36 (60.35-62.37) v 54.16 (52.45-55.86)	Sham	53	59.84 (58.34-61.35) v 55.23 (53.27-57.20)	2.35 (0.07,4.63)	.04
			Waitlist	48	59.19 (57.63-60.75) v 53.57 (51.16-55.98)	1.28 (-1.09,3.66)	.29

1 – Among patients with follow-up scores
2 –From multivariable linear regression adjusting for the baseline score and the stratification factor.

© 2022 Hershman DL et al. *JAMA Network Open.*

eTable 2. Determination of Best Model Fit for Longitudinal Data Analyses

	Row 1 = Fit statistic (-2 Log Likelihood), model degrees of freedom<br>Row 2 = Difference in fit statistic compared to specified nested model, difference in degrees of freedom, p-value by chi-square<br>Row 3 = Difference in fit statistic compared to alternative specified nested model, difference in degrees of freedom, p-value by chi-square				
	Function for Time in Model#				
Domain	Linear Time,<br>No Interaction	Linear Time,<br>Interaction with Treatment	Quadratic Time,<br>No Interaction	Quadratic Time,<br>Interaction with Treatment	Best Model
BPI Worst Pain	5022.2, 8	5020.1, 10<br>Vs linear time: 2.1, 2, p=.35	5021.0, 9<br>Vs linear time: 1.2, 1, p=.27	5015.6, 13<br>Vs linear time, no interaction: 6.6, 5, p=.25<br>Vs linear time, interaction with treatment: 4.5, 3, p=.21<br>Vs quadratic time, no interaction: 5.4, 4, p=.25	Linear Time, No Interaction
BPI Average Pain	3603.3, 8	3602.2, 10<br>Vs linear time: 1.1, 2, p=.58	3603.2, 9<br>Vs linear time: 0.1, 1, p=.75	3601.1, 13<br>Vs linear time, no interaction: 2.2, 5, p=.82<br>Vs linear time, interaction with treatment: 1.1, 3, p=.78<br>Vs quadratic time, no interaction: 2.1, 4, p=.72	Linear Time, No Interaction
BPI Pain Severity	3524.2, 8	3523.8, 10<br>Vs linear time: 0.4, 2, p=.82	3524.0, 9<br>Vs linear time: 0.2, 1, p=.66	3521.1, 13<br>Vs linear time, no interaction: 3.1, 5, p=.69<br>Vs linear time, interaction with treatment: 2.7, 3, p=.44<br>Vs quadratic time, no interaction: 2.9, 4, p=.58	Linear Time, No Interaction
BPI Pain Interference	3601.3, 8	3599.3, 10<br>Vs linear time: 2.0, 2, p=.37	3601.3, 9<br>Vs linear time: 0.0, 1, p=1.0	3596.3, 13<br>Vs linear time, no interaction: 5.0, 5, p=.42<br>Vs linear time, interaction with treatment: 3.0, 3, p=.39<br>Vs quadratic time, no interaction: 5.0, 4, p=.29	Linear Time, No Interaction
BPI Worst Stiffness	4172.8, 8	4170.7, 10<br>Vs linear time: 2.1, 2, p=.35	4172.7, 9<br>Vs linear time: 0.1, 1, p=.75	4167.2, 13<br>Vs linear time, no interaction: 5.6, 5, p=.35<br>Vs linear time, interaction with treatment: 3.5, 3, p=.32<br>Vs quadratic time, no interaction: 5.5, 4, p=.24	Linear Time, No Interaction
BPI PROMIS PI-SF	5722.3, 8	5717.2, 10<br>Vs linear time: 5.1, 2, p=.08	5721.7, 9<br>Vs linear time: 0.6, 1, p=.44	5716.3, 13<br>Vs linear time, no interaction: 6.0, 5, p=.31<br>Vs linear time, interaction with treatment: 0.9, 3, p=.83<br>Vs quadratic time, no interaction: 5.4, 4, p=.25	Linear Time, No Interaction

BPI = brief pain inventor; PROMIS PI-SF = Patient-Reported Outcome Measurement Information System Pain Interference – Short Form

Changes over all assessment times in the BPI worst pain endpoint were analyzed using linear mixed models, with individual patients treated as random effects. An unstructured correlation matrix was used. Covariates for longitudinal modeling included intervention arm (using indicator variables), assessment time, the baseline score, and the randomization stratification variables (site type, categorized as Columbia versus Kaiser NCORP versus West Michigan NCORP versus other sites, with Kaiser NCORP was the reference group). Additionally, the interactions of treatment with time and treatment with quadratic time were examined. Since models were nested, we compared log-likelihood values using a Chi-squared tests with appropriate degrees of freedom to determine which model provided a best data fit; if multiple models were identified, preference was given to the higher order model. The best model ended up being linear time with no interaction.

Model parameterizations:

- Linear, no interaction = intercept, baseline BPI worst pain score, waitlist control intervention (1=yes, 0=no), sham control intervention (1=year, 0=no), time, and the stratification factors (3 indicator variables for site type)
- Linear, interaction = intercept, baseline BPI worst pain score, waitlist control intervention (1=yes, 0=no), sham control intervention (1=year, 0=no), time, waitlist control intervention*time, sham control intervention*time, and the stratification factors (3 indicator variables for site type)
- Square, no interaction = intercept, baseline BPI worst pain score, waitlist control intervention (1=yes, 0=no), sham control intervention (1=year, 0=no), time, time*time, and the stratification factors (3 indicator variables for site type)
- Square, interaction = intercept, baseline BPI worst pain score, waitlist control intervention (1=yes, 0=no), sham control intervention (1=year, 0=no), time, time*time, waitlist control intervention*time, waitlist control intervention* time*time, sham control intervention*time, sham control intervention*time*time, and the stratification factors (3 indicator variables for site type)

© 2022 Hershman DL et al. *JAMA Network Open.*

eTable 3. Grip Strength, Maximal Contraction Over Left and Right Hand, by Randomized Arm and Time Point

Time Point	True Acupuncture<br>Mean (SD)<br>Median<br>N	Sham Acupuncture<br>Mean (SD)<br>Median<br>N	Waitlist Control<br>Mean (SD)<br>Median<br>N
Baseline	22.8 (10.0)<br>21.5<br>N=110	25.5 (15.1)<br>21.0<br>N=58	24.1 (12.1)<br>22.6<br>N=55
6 weeks	24.1 (9.5)<br>23.5<br>N=100	26.0 (16.4)<br>22.7<br>N=53	24.6 (10.3)<br>23.7<br>N=49
12 weeks	23.8 (9.8)<br>23.7<br>N=100	24.0 (13.8)<br>21.5<br>N=53	24.3 (10.6)<br>24.1<br>N=50
24 weeks	24.3 (9.2)<br>23.3<br>N=94	24.1 (11.7)<br>23.6<br>N=53	24.8 (10.7)<br>24.0<br>N=49
52 weeks	23.8 (8.8)<br>23.9<br>N=90	24.6 (8.1)<br>24.4<br>N=50	26.2 (11.0)<br>24.6<br>N=47

© 2022 Hershman DL et al. *JAMA Network Open.*

eTable 4. Grip Strength, Change in Maximal Contraction Between Follow-up and Baseline by Randomized Arm and Time Point

Time Point	True Acupuncture<br>Mean (SD)<br>Median<br>N	Sham Acupuncture<br>Mean (SD)<br>Median<br>N	Waitlist Control<br>Mean (SD)<br>Median<br>N	P value (ANOVA), difference in mean change from baseline
6 weeks	1.1 (6.3)<br>0.9<br>N=100	0.3 (11.2)<br>1.5<br>N=53	-0.2 (8.2)<br>0.6<br>N=49	0.67
12 weeks	0.9 (5.8)<br>1.3<br>N=100	-1.5 (11.3)<br>0.8<br>N=53	-0.4 (8.1)<br>0.8<br>N=50	0.20
24 weeks	1.1 (7.4)<br>1.6<br>N=94	-1.5 (12.2)<br>1.4<br>N=53	0.2 (9.0)<br>1.2<br>N=49	0.28
52 weeks	0.4 (8.7)<br>1.3<br>N=90	-1.4 (14.7)<br>1.4<br>N=50	1.2 (9.9)<br>1.6<br>N=47	0.50

© 2022 Hershman DL et al. *JAMA Network Open.*

eTable 5. Timed Get Up and Go, by Randomized Arm and Time Point

Time Point	True Acupuncture<br>Mean (SD)<br>Median<br>N	Sham Acupuncture<br>Mean (SD)<br>Median<br>N	Waitlist Control<br>Mean (SD)<br>Median<br>N
Baseline	13.2 (13.9)<br>10.6<br>N=110	11.9 (9.4)<br>10.1<br>N=58	10.8 (4.0)<br>10.0<br>N=55
6 weeks	10.4 (6.6)<br>9.5<br>N=100	10.8 (8.4)<br>9.2<br>N=53	10.0 (3.9)<br>9.0<br>N=49
12 weeks	12.1 (13.8)<br>9.1<br>N=100	10.8 (10.6)<br>9.0<br>N=53	9.7 (3.7)<br>9.0<br>N=49
24 weeks	11.5 (16.0)<br>8.9<br>N=95	10.5 (8.7)<br>8.9<br>N=53	9.5 (2.9)<br>8.4<br>N=49
52 weeks	9.5 (3.9)<br>8.7<br>N=90	9.2 (2.6)<br>8.5<br>N=50	8.9 (2.2)<br>8.6<br>N=47

© 2022 Hershman DL et al. *JAMA Network Open.*

eTable 6. Timed Get Up and Go, Change Between Follow-up and Baseline, by Randomized Arm and Time Point

Time Point	True Acupuncture<br>Mean (SD)<br>Median<br>N	Sham Acupuncture<br>Mean (SD)<br>Median<br>N	Waitlist Control<br>Mean (SD)<br>Median<br>N	P value (ANOVA), difference in mean change from baseline
6 weeks	-2.6 (11.8)<br>-1.0<br>N=100	-1.2 (3.0)<br>-0.7<br>N=53	-0.6 (3.3)<br>-0.4<br>N=49	0.35
12 weeks	-1.1 (7.6)<br>-1.1<br>N=100	-1.1 (14.2)<br>-0.8<br>N=53	-0.9 (3.5)<br>-0.4<br>N=49	0.99
24 weeks	-1.6 (11.1)<br>-1.3<br>N=95	-1.4 (3.1)<br>-1.1<br>N=53	-1.1 (3.4)<br>-0.7<br>N=49	0.94
52 weeks	-3.7 (14.8)<br>-1.4<br>N=90	-2.8 (9.9)<br>-0.9<br>N=50	-1.4 (3.5)<br>-0.6<br>N=47	0.57

© 2022 Hershman DL et al. *JAMA Network Open.*
