# Supplementary material for: Comparison of Acupuncture vs Sham Acupuncture or Waiting List Control in the Treatment of Aromatase Inhibitor–Related Joint Pain: A Randomized Clinical Trial
Source: JAMA Netw Open. 2022 Nov 11;5(11):e2241720. doi: 10.1001/jamanetworkopen.2022.41720 (PMC9652759; doi:10.1001/jamanetworkopen.2022.41720)
Supplement: Supplement 3. — Data Sharing Statement [file jamanetwopen-e2241720-s003.pdf]

Data Sharing Statement

Hershman. Comparison of Acupuncture vs Sham Acupuncture or Waiting List Control in the Treatment of Aromatase Inhibitor-Related Joint Pain. *JAMA Netw Open*. Published November 11, 2022. doi:10.1001/jamanetworkopen.2022.41720

Data
Data available: Yes
Data types: Deidentified participant data
How to access data: SWOG Statistical and Data Mangaement Center
When available: With publication

Supporting Documents
Document types: None

Additional Information
Who can access the data: researchers whose proposed use of the data has been approved
Types of analyses: for any purpose
Mechanisms of data availability: after approval of a proposal and with a signed data access agreement
